# Multispectroscopic Investigation of the Organosilyl Ether *sec*-Butoxytrimethylsilane

**DOI:** 10.1021/acs.jpca.6c00891

**Published:** 2026-04-29

**Authors:** Freya E. L. Berggötz, Emilia J. Heikura, Andreas Hans, Denis Kargin, Arno Ehresmann, Rudolf Pietschnig, Melanie Schnell

**Affiliations:** † 28332 Deutsches Elektronen-Synchrotron DESY, Notkestr. 85, 22607 Hamburg, Germany; ‡ Institut für Experimentalphysik, Universität Hamburg, Luruper Chaussee 149, 22761 Hamburg, Germany; § Institut für Physik and Center for Interdisciplinary Nanostructure Science and Technology (CINSaT), 9178 Universität Kassel, Heinrich-Plett-Straße 40, 34132 Kassel, Germany; ∥ Institut für Chemie and Center for Interdisciplinary Nanostructure Science and Technology (CINSaT), Universität Kassel, Heinrich-Plett-Straße 40, 34132 Kassel, Germany; ⊥ Institut für Physikalische Chemie, Christian-Albrechts-Universität zu Kiel, Max-Eyth-Straße 1, 24118 Kiel, Germany

## Abstract

In a multispectroscopic approach, we investigated the tailored chiral organosilicon compound *sec*-butoxytrimethylsilane in the gas phase and report its racemic and enantiopure synthesis. We characterized the structural and conformational flexibility using chirped-pulse Fourier transform microwave spectroscopy in a supersonic jet and identified three different conformers from the analysis of the rotational spectrum supported by quantum-chemical calculations. For the lowest energy conformer, characteristic splitting patterns are observed, matching the internal rotation of two nonequivalent methyl rotors, which can be attributed to two out of the three methyl groups attached to the silicon atom. Complementary synchrotron-based photoelectron spectroscopy provided the Si 2p core-level binding energies, characterizing the electronic environment of silicon in this chiral organosilicon framework. These results establish a detailed picture of both the structural dynamics and the electronic properties of this flexible silicon-containing molecule, providing a foundation for future studies of structure–property relationships in chiral organosilicon systems.

## Graphical abstract

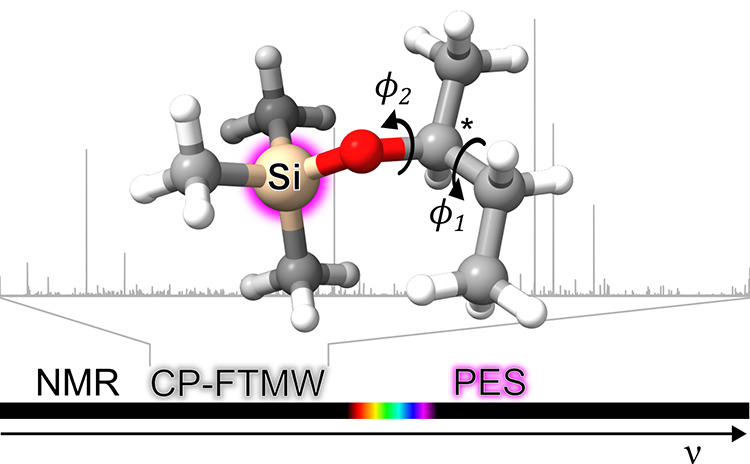

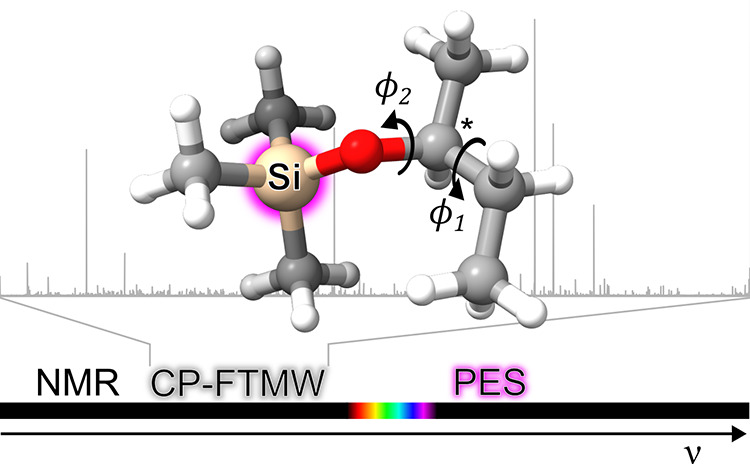

## Introduction

Over the past decades, organosilicon compounds have attracted growing attention across various fields of research owing to their versatile chemical behavior and potential as functional materials and reagents.^1^ Although silicon and carbon are neighboring group 14 elements, substituting carbon with silicon, the so-called carbon–silicon switch strategy, can profoundly alter physical and chemical properties of the molecule. These differences arise from the larger atomic radius, lower electronegativity, and distinct electronic configuration of silicon compared to carbon.^2,3^ Therefore, designing organosilicon compounds with tailored functionalities is of great interest, for example, in pharmaceutical chemistry, where silicon substitution can be used to fine-tune pharmacokinetic and physicochemical parameters.^4–7^

Further, chiral organosilicon compounds such as the silyl ether *sec*-butoxytrimethyl-silane (sBT-Si, Figure 1a), which is the focus of this study, could serve as model systems for investigating the influence of stereochemistry on key physicochemical properties. Chirality, a fundamental symmetry property, arises in molecules that exist as nonsuperimposable mirror images (enantiomers). A variety of modern techniques have been developed to investigate and control chiral molecules in the gas phase.^8^ They cover different regions of the electromagnetic spectrum, thus addressing different degrees of freedom of the molecule. One gas-phase technique for differentiating enantiomers and addressing the electronic structure of the molecules is photoelectron circular dichroism (PECD), which manifests as an asymmetry in the photoelectron angular distribution when using circularly polarized light.^9,10^

**Figure 1:**
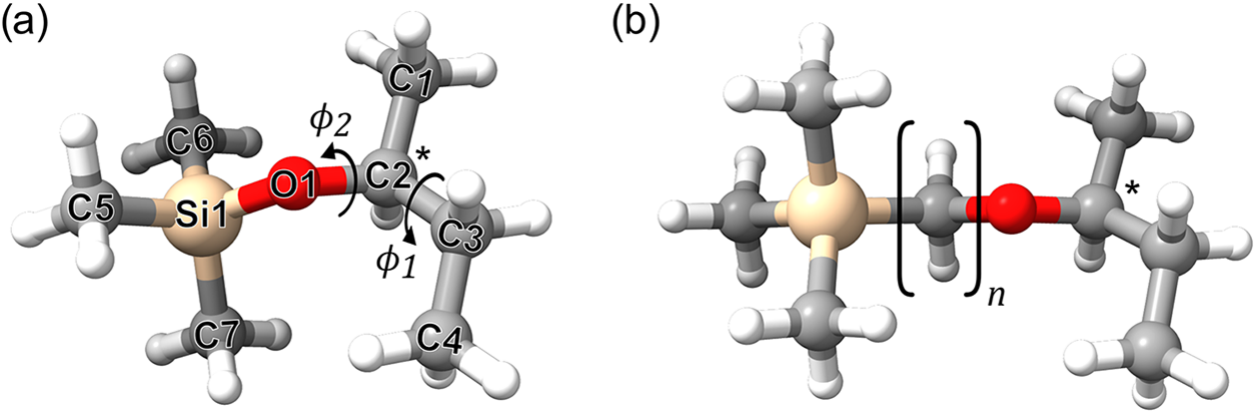
(a) Optimized molecular structure of (*S*)-*sec*-butoxytrimethylsilane, including the numbering of the non-hydrogen atoms. The stereogenic center is marked with an asterisk. The arrows indicate the two dihedral angles, ϕ_1_ and ϕ_2_, that are used to describe the conformational flexibility. (b) Silyl ether with adjustable distance between silicon and the stereogenic center by adding (CH_2_)_*n*_ groups as spacers.

With the inner-shell orbitals of Si (1s or 2p) and O (1s), sBT-Si offers different probing sites for photoionization at specific distances from the stereocenter. Additionally, the silyl ether can be chemically modified by inserting CH_2_ moieties between the O and Si atoms (Figure 1b). Thereby, the distance of silicon to the stereocenter can be gradually enlarged while the distance between oxygen and the stereocenter remains constant. This tunability makes sBT-Si an intriguing candidate for exploring PECD as a function of the distance between electron-emitter site and the stereocenter. Because the measured PECD signal is highly sensitive to molecular structure, the conformational flexibility of the compound has to be explored.

Microwave spectroscopy provides a powerful tool for characterizing molecular conformational landscapes, as the rotational spectrum of each individual molecular structure, including conformers, constitutes a unique fingerprint. In addition, the growing astrochemical interest in silicon-containing species motivates detailed rotational-spectroscopic investigations of such compounds to provide accurate line lists for radio-astronomy searches.^11,12^ Apart from obtaining structural information, microwave spectroscopy is highly sensitive to intra- and intermolecular large-amplitude motions (LAMs). The effect of silicon incorporation on such motions represents an additional point of interest, as it induces substantial changes in both steric effects and electronic energies within the substituted region. Analyzing LAMs, such as methyl internal rotation, offers valuable insight into the local chemical environment of the rotor.^13^ Here, bonding of a methyl group to silicon instead of carbon can significantly decrease the barrier of the internal motion.^14^ Inner-shell photoelectron spectroscopy using X-ray radiation provides additional information by probing the binding energies of core electrons and their sensitivity to the local chemical environment.^15^

In the present work, we combine complementary gas-phase spectroscopic techniques providing a comprehensive study of this flexible chiral organosilicon compound. We provide details on the racemic and enantiopure synthesis, the characterization of conformational flexibility and internal rotation using rotational spectroscopy supported by quantum-chemical calculations, and measurements of the silicon 2p binding energies using X-ray photoelectron spectroscopy.

## Experimental and Computational Methods

### Synthesis

Sample preparation and workup procedures, if not stated otherwise, were performed under inert conditions. Racemic *sec*-butoxytrimethylsilane was prepared according to a known literature procedure.^16^ Preparation using enantiopure *S*- or *R*-2-butanol as starting material was performed according to an adapted literature procedure.^17^ For long-term storage, the target compounds were stored under an inert argon atmosphere. *RS*-, *S*- and *R*-2-butanol (99% each) were obtained from BLD-Pharm, NaH was obtained from Merck and used as received. Chlorotrimethylsilane was obtained from Merck and hexamethyldisilazane was obtained from ABCR; both were distilled prior to usage. Acetonitrile was dried over P_4_O_10_, and diethyl ether was dried over NaK-alloy and distilled prior to usage. ^1^H and ^13^C NMR-data were recorded on Varian MR-400 MHz, Varian VNMRS-500 MHz or Jeol JNM-ECZL500 spectrometers at 25 °C. Chemical shifts were referenced to residual protic impurities in the solvent (^1^H) or the deuterio solvent itself (^13^C) and reported relative to SiMe_4_ = 0 ppm (^1^H, ^13^C). Spectroscopic assignment is in agreement with literature values.^18^ The purity of the substances was determined via integration in ^1^H NMR spectra (depiction in SI).

#### Procedure for Preparation of Racemic *sec*-Butoxytrimethylsilane

1.05 g (2.4 mmol) La­(NO_3_)_3_·6 H_2_O were dissolved in 10 mL acetonitrile, and 40 mL (32.40 g, 437.1 mmol) *R*/*S*-2-butanol were added at room temperature. Thereafter, 55 mL (42.60 g, 263.8 mmol) of hexamethyldisilazane were added rapidly, which led to a temperature increase of the mixture with reflux of the solvent, accompanied by gas evolution. After approximately 1.5 h gas evolution ceased, the mixture was subjected to aqueous workup to remove acetonitrile. The organic phase was extracted twice with 50 mL of water and dried over MgSO_4_. Distillation utilizing a vigreux column at atmospheric pressure, discarding fractions with boiling point below 107 °C, yielded 32.13 g of a clear, colorless liquid with approximately 90% purity. Subsequent redistillation with fractions investigated via 0D (pure sample without deuterated solvent) ^13^C NMR spectroscopy, combined and redistilled yielded 19.32 g (∼25 mL) of 96% pure liquid with a boiling point of 111–112 °C. Further consecutive redistillation finally yields 4.33 g (29.6 mmol, 7%) of >99% pure product as a colorless liquid with a sharp boiling point of 112 °C/101.325 kPa in agreement with the literature value 112.3 °C.^19^

#### General Procedure for Preparation of Enantiopure *sec*-Butoxytrimethyl-silanes

In a 250 mL Schlenk flask, 3.25 g (135.4 mmol) NaH was suspended in 100 mL of diethyl ether. Addition of 10.04 g (134.9 mmol) enantiopure 2-butanol ((*S*-) or (*R*-)) resulted in a gentle reflux and a colorless suspension. Reflux was maintained for approximately 2.5 h until gas evolution ceased. All volatile compounds were removed *in vacuo*. At 0 °C, 17.2 mL (134.9 mmol) of chlorotrimethylsilane were added and after complete addition, the mixture was stirred at 80 °C oil-bath temperature for 15 min and then at 140 °C for an hour. Thereafter, all volatile compounds were collected in a nitrogen cooled cold trap under reduced pressure. The crude product was transferred to a 50 mL Schlenk flask and subjected to fractional distillation at atmospheric pressure. Fractions with a boiling point below 111 °C were discarded. The fractions with a boiling point of 111–113 °C/101.325 kPa amounted to *S*: 15.11 g (103.2 mmol, 77% yield, 98% purity); *R*: 15.12 g (103.2 mmol, 77% yield, 98% purity).

### Chirped-Pulse Fourier Transform Microwave Spectroscopy

We recorded the broadband rotational spectrum of sBT-Si in the frequency range from 2 to 8 GHz using the COMPACT spectrometer,^20^ which is based on the chirped-pulse Fourier transform microwave (CP-FTMW) technique.^21,22^ A detailed description of the instrumentation and its modifications can be found elsewhere^20,23^ whereas the measurement procedure is summarized briefly below.

The compound is introduced into the vacuum chamber via a supersonic expansion providing low translational and internal temperatures. The liquid sample was placed in the reservoir of a pulsed nozzle (a modified General valve, Parker Series 9) without additional heating. By using neon as a carrier gas with a backing pressure of 2.1 bar, a rotational temperature of around *T*_*rot*_ = 0.5 K was obtained, which can be estimated by the intensity profile of the measured rotational spectrum.

The chirped microwave (MW) pulses with a duration of 4 μs were generated using an arbitrary waveform generator with a sampling rate of 24 GS/s and subsequently amplified using a 300 W traveling wave tube amplifier. After being broadcasted through a horn antenna in perpendicular direction to the pulsed molecular jet expansion, the chirped MW pulse polarizes the molecular ensemble creating a macroscopic dipole moment. The decay of this macroscopic dipole moment, the free induction decay (FID), is recorded for 40 μs as a function of time. A total of 1.6 million FIDs were collected, averaged in the time domain, and Fourier transformed with a Kaiser-Bessel window function^21,24^ to obtain the broadband rotational spectrum.

### Inner-Shell Photoelectron Spectroscopy

The Si 2p photoelectron spectrum of sBT-Si was obtained at the Pleiades beamline^25^ of the synchrotron Soleil (France). The beamline offers the required photon energies with variable circular polarization. The vapor pressure of sBT-Si was sufficient to produce a continuous molecular jet when expanding through a 0.5 mm pinhole into vacuum. The jet was skimmed using two consecutive skimmers of 1.5 mm diameter at distances of 11 and 14 cm from the interaction volume with the synchrotron beam. The energy of the ionizing photons was 110 eV. At an exit-slit width of the beamline monochromator of 50 μm, the photon bandwidth is approximately 20 meV. Photoelectron spectra were measured using a custom-built velocity map imaging (VMI) spectrometer^26,27^ operated with a position-sensitive detector based on microchannel plates (MCPs) and a delay line anode with 75 mm diameter active area (“RoentDek DLD75”). The sample was recycled using a closed-loop recycling system.^28^ The photoelectron kinetic energies were calibrated by photoionization of krypton and helium at several excitation photon energies. The resolution of the VMI spectrometer was estimated to be Δ*E* = 1 eV within the kinetic energy range of interest.

### Computational Methods

The experiments are supplemented by quantum-chemical calculations. The conformational space of sBT-Si was sampled at the extended tight-binding level GFN2-xTB using the program package CREST (conformer-rotamer ensemble sampling tool)^29^ resulting in a set of geometries that were further optimized at a higher level of theory. Geometry optimizations and conformational conversion barriers were performed with the program package ORCA 5.0 ^30,31^ using the exchange-correlation functional B3LYP^32^ (Becke, three-parameter, Lee–Yang–Parr) including the dispersion correction (D3) by Grimme^33,34^ and Becke-Johnson (BJ) damping^35^ as well as the basis set def2-TZVP with the auxiliary basis def2/J.^36,37^ Harmonic frequency calculations were performed to determine the zero-point energy (ZPE) corrected energies and to validate that the energy minima of the conformers are real. Predictions of the barriers for conformational conversion and methyl internal rotation were obtained by performing one-dimensional dihedral angle scans using the same functional and basis set. The interconversion of conformers was further explored with nudged elastic band (NEB) calculations.^38^

Using ORCA, the binding energy of the Si 2p orbital was calculated using the Δ-self-consistent-field (ΔSCF) method, which considers the energy difference between the neutral parent and the ionized species.^39^ For these calculations, the functional B3LYP in combination with the dispersion correction D3­(BJ) and the basis set cc-pCVTZ^40–42^ was used. The generalized mode following method^43^ was added to help with the convergence of the ΔSCF excited state solution.

## Results and Discussion

### Theoretical Results Describing the Conformational Landscape

The investigation of the conformational landscape using CREST and further structure optimization at the DFT level resulted in eight distinct structures with relative energies up to 10 kJ mol^–1^. The calculated vibrational modes confirmed that all eight structures are true minima on the potential energy surface. Table 1 summarizes their rotational constants, electric dipole moment components, and relative ZPE corrected energies calculated at the B3LYP-D3­(BJ)/def2-TZVP level of theory. The conformational flexibility of sBT-Si is governed primarily by two dihedral angles, defined as ϕ_1_ = ∠(C1–C2–C3–C4) and ϕ_2_ = ∠(Si1–O1–C2–C1), that describe the arrangement of the butyl chain and the orientation of the butyl moiety with respect to the rest of the molecule (Figure 1a). The values of both dihedral angles for the different conformers are also listed in Table 1. Structures are labeled according to the configurations of ϕ_1_ and ϕ_2_, with dihedral angles of ±180° ± 30°, ±60° ± 30°, and 120° ± 30° corresponding to the *anti* (a), *gauche+* (g+), *gauche*– (g−), and *anticlinal* (ac) conformations, respectively.

**Table 1:** Relative ZPE Corrected Energies, Δ*E*_0_, Rotational Constants, Dipole Moment Components μ_*i*_ with *i* = *a*,*b*,*c*, and Dihedral Angles ϕ_1_ and ϕ_2_ for the Conformers of sBT-Si within 10 kJ mol^–1^ Calculated at the B3LYP-D3­(BJ)/def2-TZVP Level of Theory^a^

	aac*	g–ac*	g+ac*	g+a	g–g+	g+g+	ag–1	ag–2
*A* (MHz)	1713.3	2059.1	1896.4	1826.0	2207.9	1961.2	1730.4	1683.1
*B* (MHz)	912.7	741.4	808.9	865.9	702.8	782.8	947.0	971.8
*C* (MHz)	753.8	679.8	738.3	772.5	658.9	729.8	790.0	801.3
|μ_*a*_|(D)	0.1	0.2	0.1	0.1	0.2	0.0	0.4	0.6
|μ_*b*_|(D)	0.2	0.2	0.3	0.1	0.5	0.8	0.6	0.2
|μ_*c*_|(D)	0.9	0.9	0.9	0.9	0.7	0.5	0.7	0.9
ϕ_1_ (°)	–178	–63	61	63	–65	60	–178	–170
ϕ_2_ (°)	105	118	119	159	72	75	–46	–71
Δ*E*_0_ (kJ mol^–1^)	0.0	3.4	4.2	5.9	6.3	6.9	7.7	10.0

a The conformers observed in the experimental spectrum are indicated with an asterisk.

The three lowest energy conformers, aac, g–ac, and g+ac, differ mainly in the orientation of the butyl chain (ϕ_1_), which adopts the three orientations *anti*, *gauche*–, and *gauche+* due to the four sp^3^-hybridized carbon atoms. The ϕ_2_ dihedral angle differs only slightly for these structures. Similar to *n*-butane,^44^ the *anti* orientation (conformer aac) is the lowest in energy, followed by the *gauche*– (conformer g–ac) and *gauche+* (conformer g+ac) orientation, with the latter two being almost isoenergetic. Starting from conformer aac, a relaxed one-dimensional scan of the potential energy surface was performed along the dihedral angle ϕ_1_, revealing the distinct minima of the three lowest energy conformers (Figure 2). The barriers between the minima are well above 1000 cm^–1^ and prohibit conformational relaxation during the supersonic expansion.^45^

**Figure 2:**
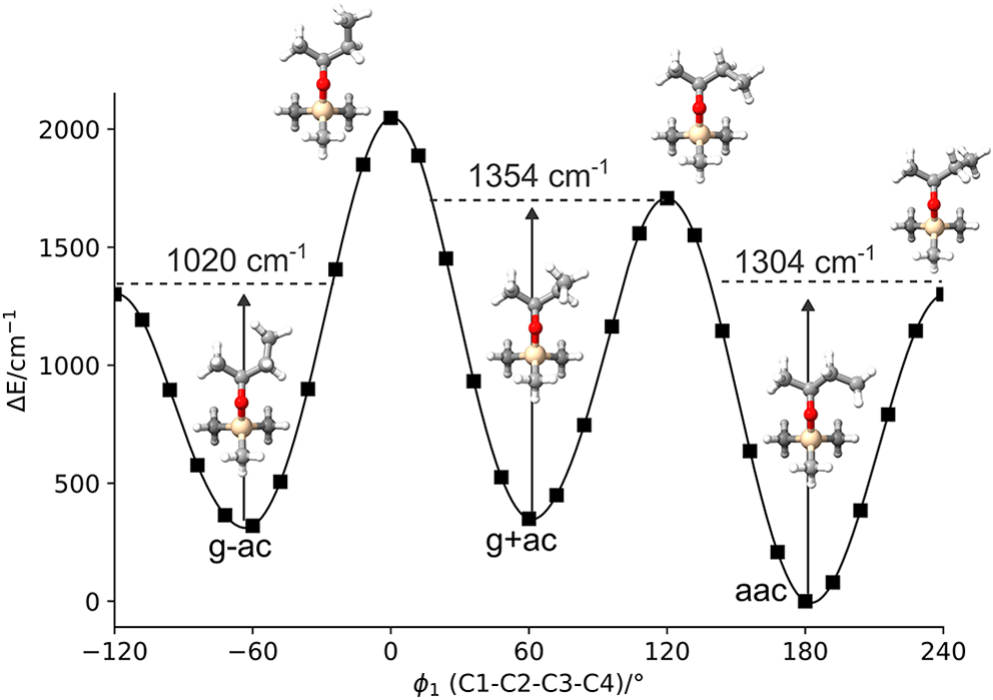
One-dimensional potential energy scan of the dihedral angle ϕ_1_ (the butyl group) calculated at the B3LYP-D3­(BJ)/def2-TZVP level of theory. An interpolation of the scan is shown that consists of 30 relaxed single point calculations with a step size of 12° (black markers). The barrier heights for interconversion (not ZPE-corrected) are noted, and the geometries of the minima and the transition states are depicted.

The other five geometries that are higher in energy, with their relative energies given in Table 1, correspond to *anti*, *gauche+*, or *gauche–* arrangements of the ϕ_1_ angle and different orientations of the ϕ_2_ angle. For these higher-energy conformers, relaxation barriers were predicted by performing NEB calculations. All possible pathways for each conformer relaxing to a lower energy configuration were calculated at the B3LYP-D3­(BJ)/def2-TZVP level of theory. The calculations reveal that the higher energy conformers can relax to one of the three lowest energy conformers by rotating around the ϕ_2_ dihedral angle, while the orientation of the butyl chain (ϕ_1_) is preserved (Table 2). Except for the interconversion of ag–1 to aac, all barriers are below 30 cm^–1^ and relaxation is possible in the course of the supersonic expansion. The low relaxation barriers were confirmed by higher-level single-point energy calculations at the DLPNO–CCSD­(T)/cc-pVTZ level of theory, based on the initial structures and the transition states obtained from the preceding NEB calculations.

**Table 2:** Overview of the Conformational Relaxation Barriers Δ*E* of the Five Conformers Higher in Energy within An Energy Window of 10 kJ mol^–1^ Computed at Two Different Levels of Theory

conformers	Δ*E* ^a^ (cm^–1^)	Δ*E* ^b^ (cm^–1^)
g+a → g+ac	29	4
g–g+ → g–ac	11	6
g+g+ → g+ac	9	11
ag–1 → aac	375	386
ag–2 → ag–1	24	66

a Barriers obtained from NEB calculations at the B3LYP-D3­(BJ)/def2-TZVP level of theory.

b Energy difference using single-point energy calculations at the DLPNO–CCSD­(T)/cc-pVTZ level of theory for the respective higher-energy conformer and the transition state, which was obtained from the NEB scan.

The relaxation barrier of ag–1 to aac with approximately 380 cm^–1^, which is close to the empiric limit of conformational conversion in a supersonic beam using neon (∼400 cm^–1^),^46^ might prohibit a full relaxation. However, due to a rather high relative energy of 7.7 kJ mol^–1^, the abundance of ag–1 can be expected to be low. Similarly, the highest energy conformer in the given range of 10 kJ mol^–1^, ag–2, could first relax to ag–1 with a low interconversion barrier and then further relax to aac. Thus, all the higher energy conformers within the given energy window can either relax during the supersonic expansion or are relatively high in energy and consequently low in abundance, and thereby expected not to be detectable.

### Chirped-Pulse Fourier Transform Microwave Spectroscopy

On the basis of the previous calculations, we put our initial main focus on the three lowest energy conformers, as discussed above. Starting with the theoretically predicted rotational parameters, rotational transitions belonging to the conformers aac, g–ac, and g+ac were identified in the spectrum and fit using a semirigid rotor Hamiltonian employing Watson’s A reduction^47^ and I^r^ representation using the program PGOPHER.^48^ The assignment of the aac conformer was straightforward due to its strong rotational transitions. Removing the lines belonging to conformer aac revealed less intense spectral patterns, and the conformers g–ac and g+ac could be identified. Their transitions were more than one order of magnitude weaker compared to the global minimum, and fewer lines could be assigned. The fitted rotational constants of the assigned species are presented in Table 3, and the simulated spectra based on the fitted parameters are illustrated in Figure 3. The calculated rotational constants (Table 1) are in good agreement with the values obtained experimentally. For all three conformers, *c*-type transitions dominate, in agreement with the predicted magnitude of the dipole-moment components, μ_*c*_ > μ_*b*_ ≈ μ_*a*_. We could not observe any higher energy conformers, which is consistent with the expected conformational relaxation as discussed above.

**Figure 3:**
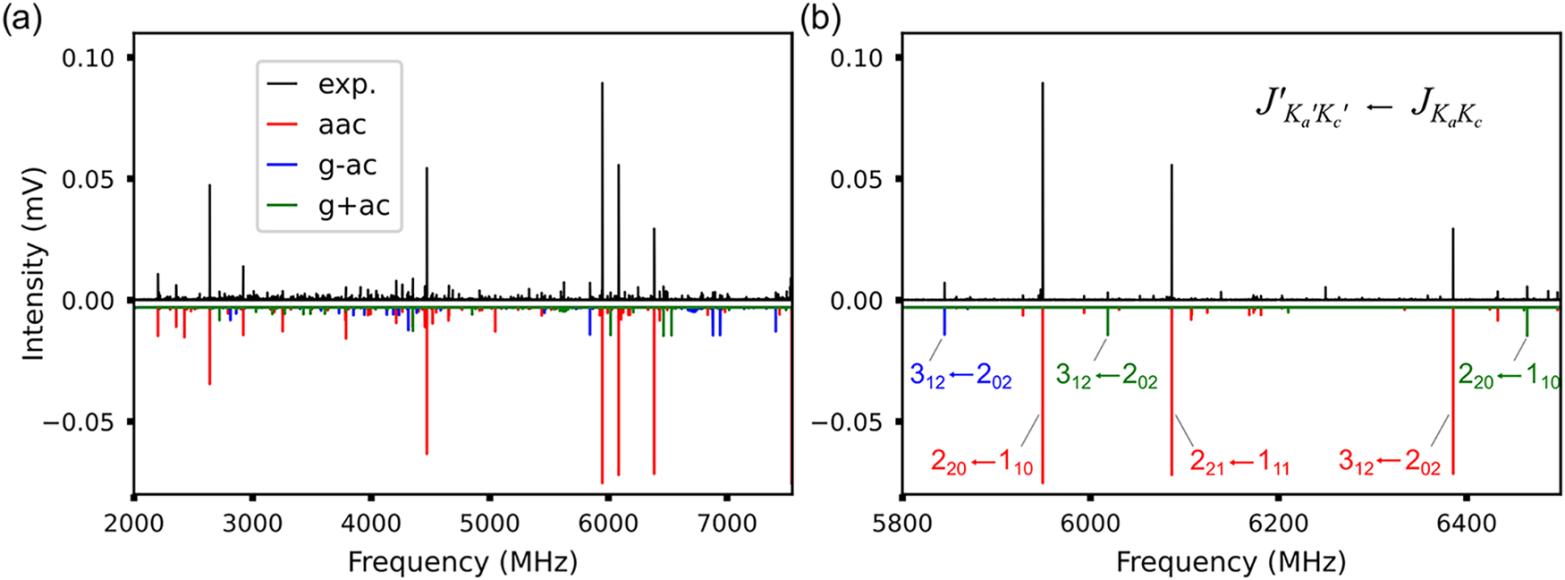
Experimental rotational spectrum of sBT-Si (upper trace, in black) and the simulated rotational spectra (lower trace) employing the fitted rotational parameters as given in Table 3 at the fitted rotational temperature of 0.426 K for the three assigned conformers. (a) Overview of the whole spectrum measured from 2 to 8 GHz. (b) Zoom-in of the spectrum, highlighting some transitions *J*_*K*_*a*_^′^ *K*_*c*_^′^_^′^ ← *J*_*K*_*a*_ *K*_*c*__ of the assignments.

**Table 3:** Experimentally Determined Rotational Constants and Centrifugal Distortion Constants of the Three Lowest Energy Conformers, aac, g–ac, and g+ac, Obtained from the Semi-Rigid Rotor Fits Performed with PGOPHER

parameters^a^	aac	g–ac	g+ac
*A* (MHz)	1723.79632(58)	2063.95086(85)	1905.6210(17)
*B* (MHz)	915.16202(33)	749.79286(50)	815.23370(94)
*C* (MHz)	756.84885(35)	687.53303(68)	744.20351(92)
Δ_*J*_ (kHz)	0.0524(61)	0.089(15)	1.73(19)
Δ_*K*_ (kHz)	–0.114(32)	-	-
Δ_*JK*_ (kHz)	0.293(22)	-	4.72(73)
δ_*J*_ (kHz)	0.0219(29)	-	-
δ_*K*_ (kHz)	–0.581(53)	-	-
N^b^ (*a*|*b*|*c*)	78 (10|19|49)	18 (5|0|13)	17 (0|3|14)
σ^c^ (kHz)	6.9	6.2	7.6

a Watson’s *A* reduction in *I* ^r^ representation was used.

b *N* is the total number of assigned transitions; *a*,*b*,*c* refer to the number of *a*-, *b*-, and *c*-type transitions, respectively.

c σ is the standard deviation of the fit.

Using the Fit Intensity option in PGOPHER, the rotational temperature and relative abundances were fitted by analyzing the observed line intensities, which are proportional to the square of the transition dipole moment. This procedure yields relative abundances of 1.000(80): 0.247(41): 0.183(35) for aac: g–ac: g+ac at a fitted rotational temperature of 0.426(50) K. These values are in convincing agreement with the Boltzmann populations derived from the calculated relative energies using a temperature of 298.15 K, reflecting the initial room temperature conditions of the sample, which predict relative populations of 1:0.25:0.18 for aac: g–ac: g+ac.

### Internal Rotation

The rotational spectrum of sBT-Si is further complicated due to splittings of the rotational transitions, which is caused by methyl internal rotation (Figure 4b) and is especially noticeable for the lowest energy conformer due to its strong rotational transitions. The three-fold internal rotation barriers of the five methyl groups were calculated for the aac conformer using one-dimensional dihedral scans of the respective torsional angles (Figure S7 in the Supporting Information). The methyl groups are labeled as Me*X* referring to the *X*th carbon atom. The methyl groups Me1 and Me4 at both ends of the butyl chain have calculated barriers of around 13 and 12 kJ mol^–1^ (Figure 4a), respectively. This is consistent with previous work involving methyl groups connected to sp^3^-hybridized carbon atoms. The splitting caused by such high barriers is expected to be below the resolution power of the COMPACT instrument. However, since the Si–C bond is longer compared to the C–C bond (that is 1.87–1.88 Å and 1.52 Å, respectively, according to the calculated structure in Figure 1), the methyl groups attached to the Si atom, Me5, Me6, and Me7, experience less steric hindrance, and their internal rotation barriers are within 5–7 kJ mol^–1^, which can cause resolvable splittings (Figure 4b). The barriers of Me6 and Me7 are predicted to be higher compared to Me5 due to weak interactions with Me1 and Me4, respectively. This can be revealed by analyzing the noncovalent interactions (NCI)^49^ within the molecule using the program Multiwfn.^50,51^ Figure 4a depicts the isosurface of the reduced density gradient at a value of 0.5 colored according to the electron density, multiplied with the sign of the second eigenvalue of its Hessian matrix, sign­(λ_2_) ρ. The green surfaces indicate weak van der Waals interactions between the methyl rotors.

**Figure 4:**
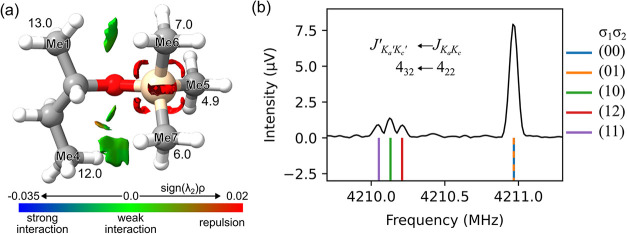
(a) NCI isosurface of the reduced density gradient at a value of 0.5 colored according to sign­(λ_2_) ρ and barriers to internal rotation *V*_3_ in kJ mol^–1^ calculated at the B3LYP-D3­(BJ)/def2-TZVP level of theory for the lowest energy conformer aac. (b) Top trace (black): experimentally observed splitting pattern of the rotational transition *J*_*K*_*a*_^′^ *K*_*c*_^′^_^′^ ← *J*_*K*_*a*_ *K*_*c*__= 4_32_ ← 4_22_; bottom trace: five different symmetry species employing the fitted rotational parameters as given in Table 4 considering the internal rotation of the two methyl rotors Me5 and Me6.

Since sBT-Si does not possess any symmetry element, the point group of the molecule is *C*_1_ and all methyl rotors are inequivalent. In the case of one methyl rotor, each rotational transition splits into the nondegenerate *A* state, which can be well predicted by a semirigid-rotor Hamiltonian excluding internal rotation, and the doubly degenerate *E* state. The *A* and *E* states can be labeled with the index σ = 0 and σ = 1,2, respectively. Considering the internal motion of three inequivalent methyl rotors, a total of 14 different symmetry species would be expected for each rotational transition.^52^ As visible in the experimental spectrum in Figure 4b, only four peaks can be resolved. This resembles splitting patterns observed in molecules with two methyl rotors consisting of a narrow doublet with the symmetry indices σ_1_σ_2_ = (00) and (01), and a triplet with (10), (11), and (12).^53^ Here, we consider two methyl rotors in our fit to effectively describe the observed splitting patterns. All three combinations—Me5-Me6, Me5-Me7, and Me6-Me7—were tested to assess which one best reproduces the observed pattern.

Using the program XIAM,^54^ we first reproduced the semirigid rotor fit by including the 78 observed rotational transitions, which correspond to the (00) species, and adopting the rotational constants and quartic centrifugal distortion constants obtained from PGOPHER (Table 3). The theoretical *V*_3,*i*_ barriers as well as the angles δ_*i*_ (angle between the internal rotation axis and the principal *a* axis) and ϵ_*i*_ (angle between the principal axis *b* and the projection of the internal rotation axis onto the *bc*-plane) for the *i*th methyl group of the pair were used to predict the other symmetry species. For all three fits, a total of 207 lines were included in a step-by-step procedure. Table 4 summarizes the obtained values for the rotational constants and quartic distortion constants, as well as the calculated angles ϵ_*i*_ and δ_*i*_, the internal rotation constant *F*_0_, and the fitted *V*_3,*i*_ barriers of the methyl rotors, respectively.

**Table 4:** Molecular Parameters of the Lowest Energy Conformer aac Obtained with XIAM for the Three Possible Methyl Rotor Combinations Me5-Me6, Me5-Me7, and Me6-Me7^a^

parameter	unit	Me5-Me6	Me5-Me7	Me6-Me7
*A*	MHz	1723.78920(45)	1723.7891(13)	1723.7887(22)
*B*	MHz	915.162270(32)	915.16157(92)	915.1585(15)
*C*	MHz	756.846266(30)	756.84644(86)	756.8491(14)
Δ_*J*_	kHz	0.0644(59)	0.083(17)	0.101(29)
Δ_*K*_	kHz	–0.114(26)	–0.216(75)	–0.44(12)
Δ_*JK*_	kHz	0.281(22)	0.337(63)	0.50(10)
δ_*J*_	kHz	0.0153(24)	0.0189(71)	0.023(12)
δ_*K*_	kHz	–0.520(56)	–0.66(16)	–0.99(27)
*V*_3,1_	kJ mol^–1^	5.54203(58)	5.5429(17)	5.1358(27)
ϵ_1_ ^b^	rad	[1.273]	[1.273]	[0.502]
δ_1_ ^b^	rad	[0.992]	[0.992]	[1.195]
*F*_0,1_ ^b^	GHz	[160.398]	[160.398]	[160.297]
*V*_3,2_	kJ mol^–1^	8.390(15)	5.453(39)	5.467(67)
ϵ_2_ ^b^	rad	[0.502]	[2.42]	[2.42]
δ_2_ ^b^	rad	[1.195]	[1.592]	[1.592]
*F*_0,2_ ^b^	GHz	[160.297]	[159.647]	[159.647]
no. of lines		207	207	207
σ_rms_ ^c^	kHz	9.5	27.5	45.7

a In *V*_3,*i*_, ϵ_*i*_, δ_*i*_, and *F*_0,*i*_, the subscript *i* = 1,2 refers to the first and second rotor of the pair, respectively.

b The angles ϵ and δ and the internal rotation constants *F*_0_ were fixed to calculated values, level of theory: B3LYP-D3­(BJ)/def2-TZVP.

c Standard deviation of the fit.

The fit using the Me5-Me6 combination yields the lowest standard deviation (9.5 kHz) and rotational as well as quartic centrifugal distortion constants that are in good agreement with those obtained from the semirigid rotor fit performed with PGOPHER, involving only the (00) lines (Table 3). A simulation based on the fitted parameters of this effective two-top model reproduces the observed splitting patterns (Figure 4b). Note that the barrier obtained for methyl rotor Me6 has lower accuracy compared to Me5. This is mainly due to the fact that the narrow doublet (00) and (01) with a predicted frequency difference of less than 20 kHz cannot be resolved in our experiment.^55^ According to the calculations, the barrier for Me6 is higher than that for Me7 (7.0 kJ mol^–1^ compared to 6.0 kJ mol^–1^, see Figure 4), however, this difference lies within the uncertainty of the calculation. A prediction of the spectrum including all three methyl rotors, using the *V*_3_ barriers obtained from the Me5-Me6 fit and an intermediate barrier for Me7 such that *V*_3_(Me5) < *V*_3_(Me7) < *V*_3_(Me6) (in line with the calculations, Figure 4a) indicates that the additional splitting introduced by Me7 would likely remain unresolved at the present experimental resolution - even for cases with lower *V*_3_ barrier for Me7 than for Me6 (Figure S8 in the Supporting Information).

The fits based on the Me5-Me7 and Me6-Me7 combinations result in standard deviations of 27.5 kHz and 45.7 kHz, respectively, and appear to be less suitable to describe the observed spectral features. The difference of the *V*_3_ barrier heights obtained for Me5 (5.56203(58) kJ mol^–1^) and Me6 (8.390(15) kJ mol^–1^) reflects the variation of the local steric environment of these methyl groups. Overall, the values are similar to those determined in other studies, e.g., for one methyl rotor in methylphenylsilane (6.7(3) kJ mol^–1^)^14^ or three methyl rotors in the *C*_3*v*_ symmetric (CH_3_)_3_SiCl (6.90(1) kJ mol^–1^).^56^

### Silicon 2p Photoelectron Spectrum

The measured photoelectron spectrum after background subtraction with an accumulation time of 30 min is presented in Figure 5. The binding energies for the two atomic fine-structure components of Si 2p_3/2_ and Si 2p_1/2_ are 99.4 and 99.8 eV, respectively.^57^ Since the energy resolution of our experiment is lower than the fine-structure splitting, a single peak was observed (Figure 5) from the molecular Si 2p orbitals. However, information on the fine-structure splitting was used to fit the photoelectron peak. The areas of the peaks (branching ratio Si 2p_1/2_/Si 2p_3/2_ = 1/2) and the energy splitting (0.4 eV) between the Si 2p_3/2_ and Si 2p_1/2_ components were fixed to perform the fit to determine the binding energies.^57^ In addition, the widths of the two peaks were assigned to be equal. The binding energy for Si 2p photoelectrons in sBT-Si given by the fit was determined to be 106.47 eV ± 0.22 eV. The energies for the two Si 2p_3/2_ and Si 2p_1/2_ fine-structure components given by the fit were correspondingly 106.78 eV ± 0.22 eV and 106.28 eV ± 0.22 eV.

Employing the ORCA program package, the Si 2p binding energies for the three lowest-energy conformers aac, g–ac, and g+ac of sBT-Si were calculated resulting in 106.1 eV for each conformer. These calculated values are in good agreement with the experimental results. Compared to atomic Si 2p, the chemical shift is about 7.0 eV to higher binding energies. A shift toward higher binding energies is expected since the high electronegativity of the binding partner oxygen typically leads to a depletion of electron density at silicon, which in turn increases the effective nuclear charge for the Si 2p photoelectrons along with a blueshift.

**Figure 5:**
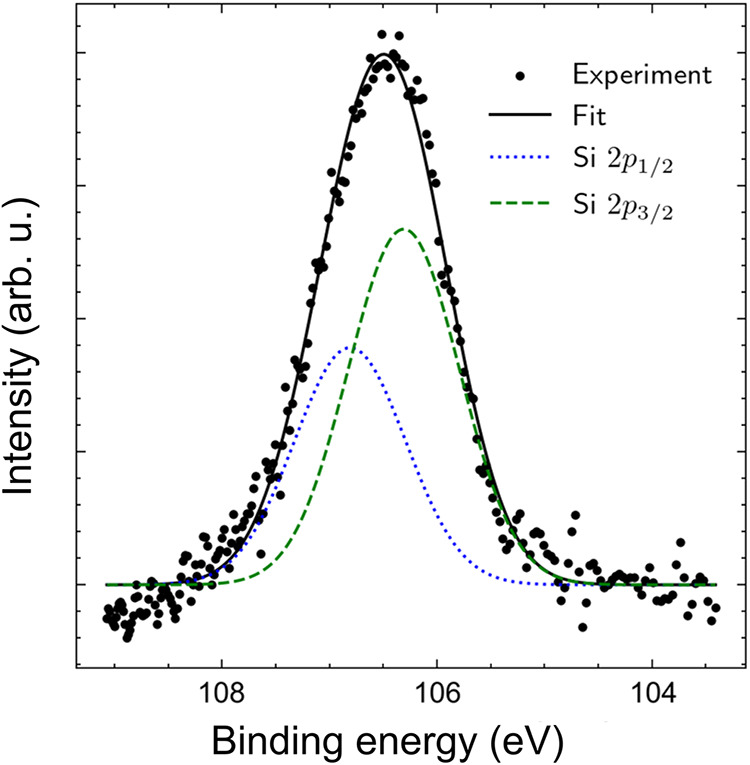
2p photoelectron spectrum of *sec*-butoxytrimethylsilane after ionization with 110 eV circularly polarized light.

## Conclusions

In this study, we characterized sBT-Si using a multispectroscopic approach focusing on microwave and photoelectron spectroscopy in the gas phase and additional NMR spectroscopy in the condensed phase. The molecular class of substituted silyl ethers is interesting for more detailed studies of molecular chirality using photoelectron circular dichroism because the distance between the stereogenic center and the PECD excitation site can be varied by adding CH_2_ moieties as spacers. However, the presence of C–C single bonds and CH_3_ groups results in high structural and conformational flexibility, which complicates the analysis.

We investigated the structural flexibility of sBT-Si using CP-FTMW spectroscopy and its Si 2p photoelectron spectrum employing the Pleiades beamline at Soleil. Supported by quantum-chemical calculations, we identified three conformers in the measured rotational spectrum and found the orientation of the butyl chain as the primary degree of freedom governing the conformational flexibility. The incorporation of silicon to form the Si­(CH_3_)_3_ moiety substantially reduces the internal rotation barriers of the methyl rotors compared to C­(CH_3_)_3_, giving rise to characteristic line splittings. The observed splitting patterns can be reproduced by an effective two-top model.

Complementary photoelectron spectroscopy with synchrotron radiation yields the binding energy of the Si 2p electron in a typical organosilicon environment featuring three carbon substituents and a single oxygen atom. Knowledge about both the conformational space and the electron binding energy is crucial information for potential future PECD measurements. While sBT-Si is suitable for gas-phase spectroscopic measurements, its conformational flexibility is expected to complicate PECD measurements. This complexity would increase further by inserting CH_2_ groups (see Figure 1b), suggesting that structurally more rigid chiral systems may be more advantageous for such investigations.

## Supplementary Material



## Notes

^1^H and ^1^3C NMR spectra for the racemic and enantiopure compounds; line lists of observed transition frequencies for all three assigned conformers, dihedral scans for the five methyl rotors of conformer aac; prediction for three methyl rotors using XIAM; comparison of the three combinations for the effective two-top fits; XIAM output files for the three different methyl rotor combinations (PDF)
