# Supplementary material for: Multispectroscopic Investigation of the Organosilyl Ether sec-Butoxytrimethylsilane
Source: J Phys Chem A. 2026 Apr 29;130(18):3592–600. doi: 10.1021/acs.jpca.6c00891 (PMC13158988; doi:10.1021/acs.jpca.6c00891)
Supplement: Supplementary file 1 [file jp6c00891_si_001.pdf]

Supporting information:

Multi-spectroscopic investigation of the organosilyl ether sec-butoxytrimethylsilane

Freya E. L. Berggötz,[†,‡] Emilia J. Heikura,[¶] Andreas Hans,[¶] Denis Kargin,[§] Arno Ehresmann,[*,¶] Rudolf Pietschnig,[§] and Melanie Schnell[*,†,‖]

†*Deutsches Elektronen-Synchrotron DESY, Notkestr. 85, 22607 Hamburg, Germany*

‡*Institut für Experimentalphysik, Universität Hamburg, Luruper Chaussee 149, 22761 Hamburg, Germany*

¶*Institut für Physik and Center for Interdisciplinary Nanostructure Science and Technology (CINSaT), Universität Kassel, Heinrich-Plett-Straße 40, 34132 Kassel, Germany*

§*Institut für Chemie and Center for Interdisciplinary Nanostructure Science and Technology (CINSaT), Universität Kassel, Heinrich-Plett-Straße 40, 34132 Kassel, Germany*

‖*Institut für Physikalische Chemie, Christian-Albrechts-Universität zu Kiel, Max-Eyth-Straße 1, 24118 Kiel, Germany*

E-mail: ehresmann@physik.uni-kassel.de; melanie.schnell@desy.de

Contents

1 Supplementary information for synthesis **S3**

2 Supplementary information for microwave spectroscopic part **S7**

1 Supplementary information for synthesis

^{1}H NMR (400 MHz, $CDCl_3$) δ (ppm): 3.67 (tq, J = 6.0, 6.2 Hz, 1H, CH), 1.53 – 1.31 (m, 2H, CH_2), 1.11 (d, J = 6.2 Hz, 3H, CH_3), 0.86 (t, J = 7.4 Hz, 3H, CH_3), 0.10 (s, 9H, $Si(CH_3)_3$). **^{13}C NMR** (101 MHz, $CDCl_3$) δ (ppm): 70.1 (CH), 32.5 (CH_2), 23.6 (CH_3), 10.4 (CH_3), 0.3 ($Si(CH_3)_3$).

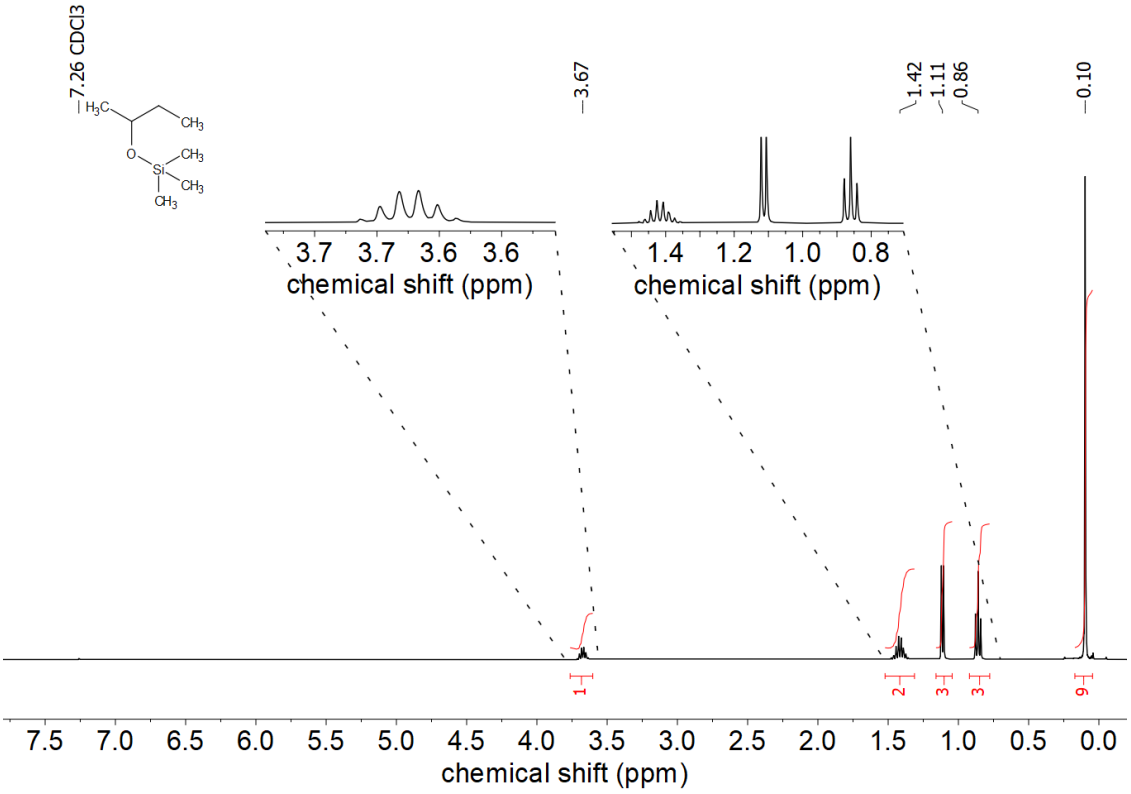

Figure S1: ^{1}H NMR ($CDCl_3$, 400 MHz) of *RS*-2-butoxytrimethylsilane with expansion of selected regions.

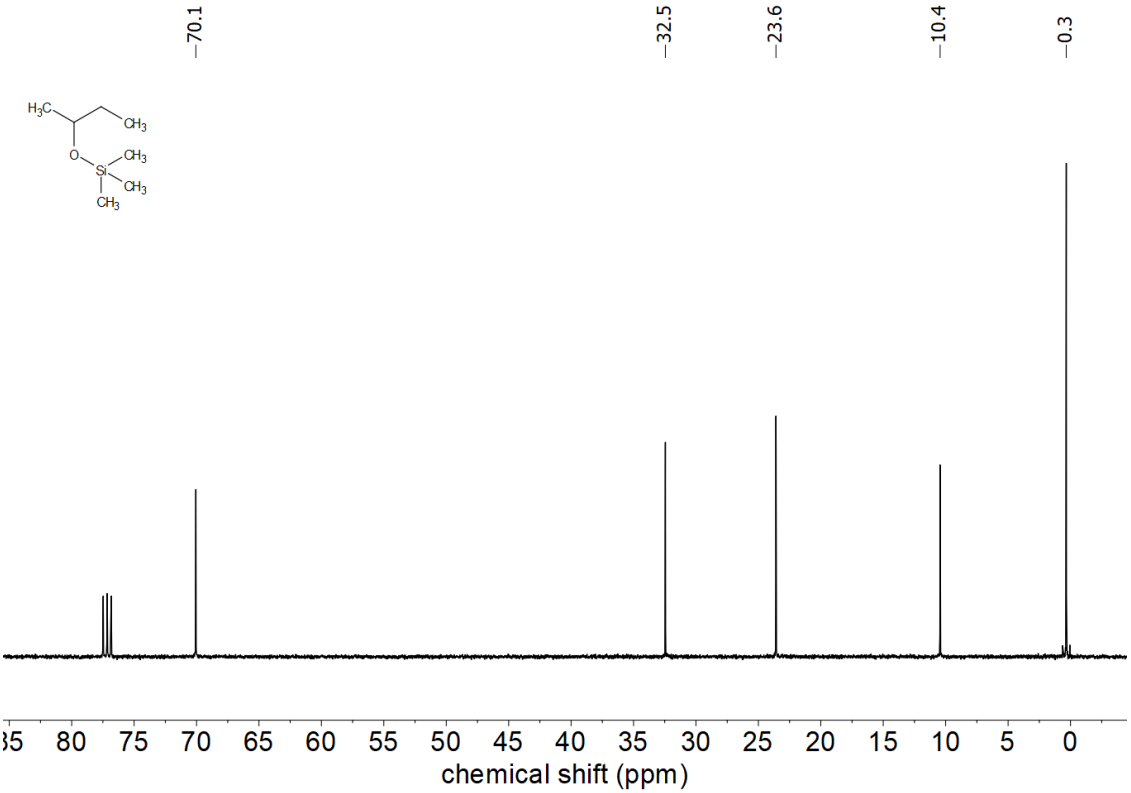

Figure S2: ^{13}C NMR ($CDCl_3$, 101 MHz) of *RS*-2-butoxytrimethylsilane

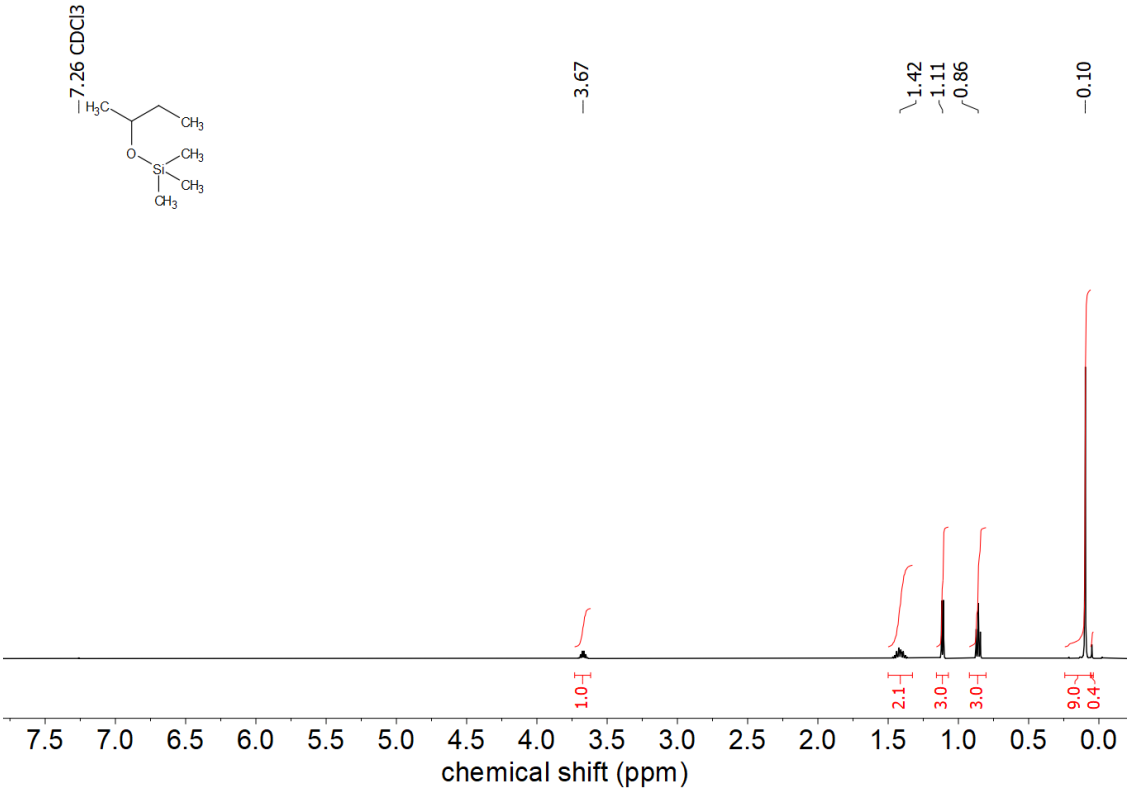

Figure S3: ^{1}H NMR ($CDCl_3$, 500 MHz) of *R*-2-butoxytrimethylsilane

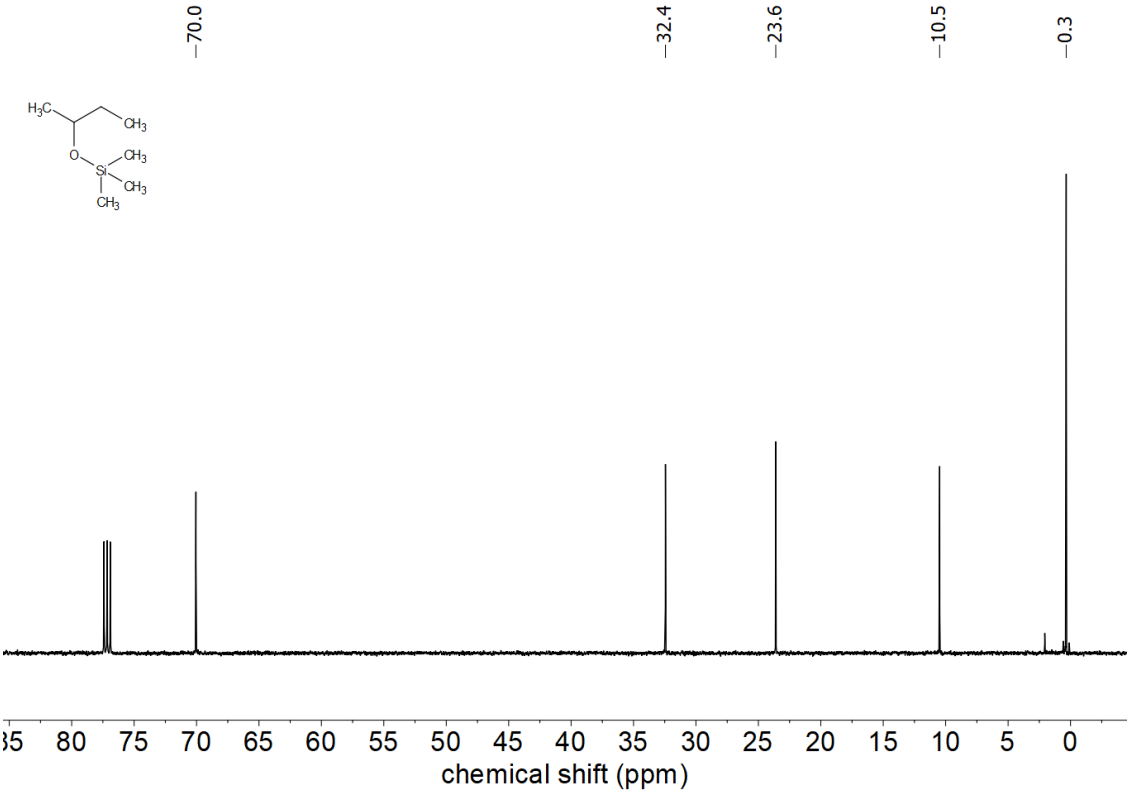

Figure S4: ^{13}C NMR ($CDCl_3$, 126 MHz) of *R*-2-butoxytrimethylsilane

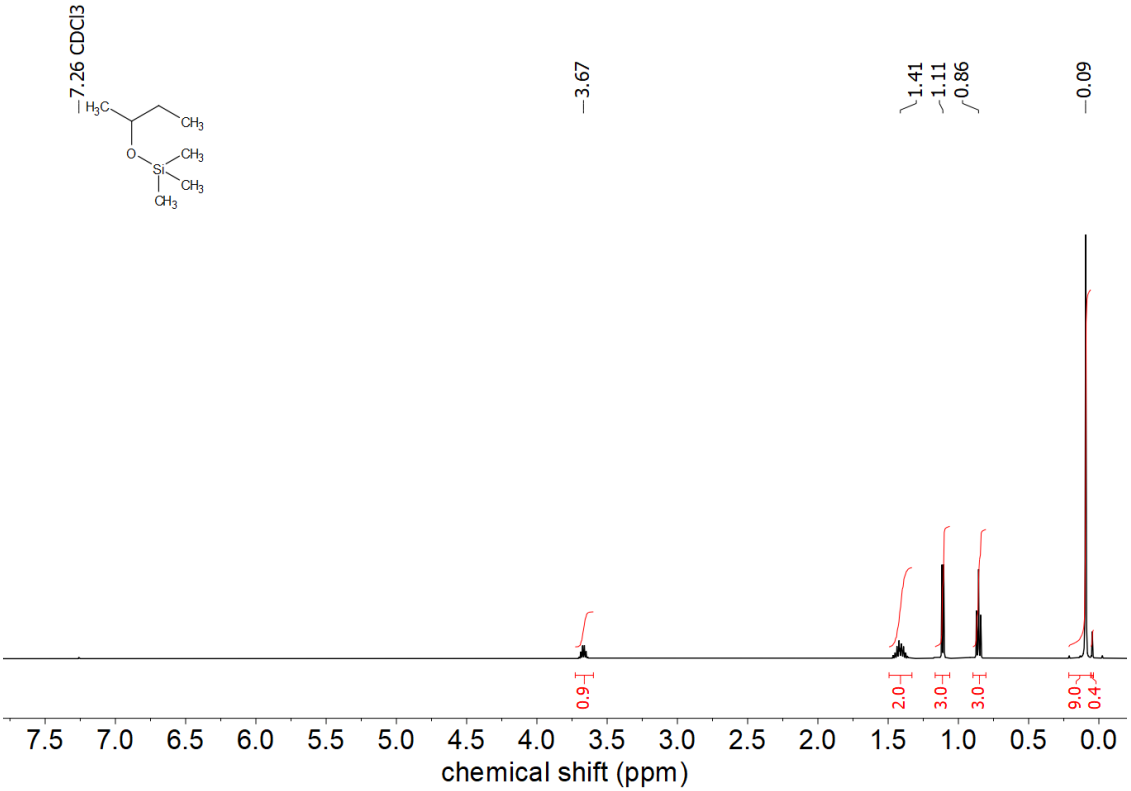

Figure S5: ^{1}H NMR ($CDCl_3$, 500 MHz) of *S*-2-butoxytrimethylsilane

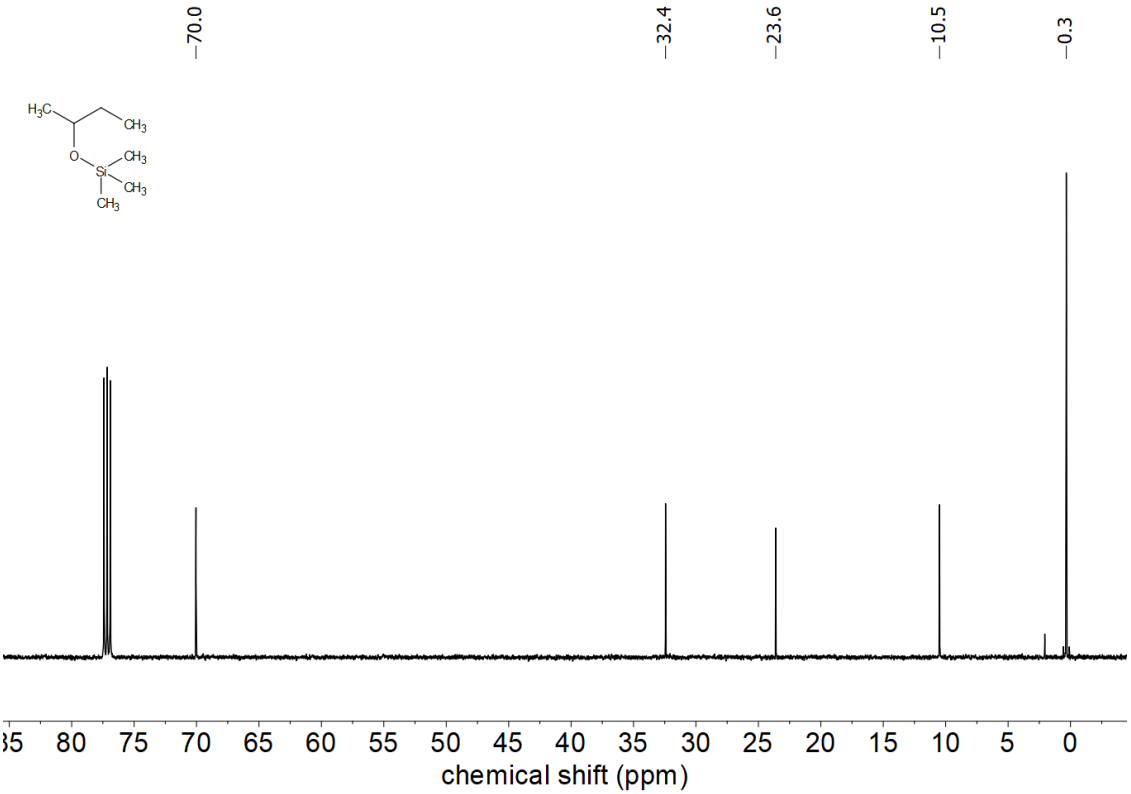

Figure S6: ^{13}C NMR ($CDCl_3$, 126 MHz) of *S*-2-butoxytrimethylsilane

2 Supplementary information for microwave spectroscopic part

Table S1: Observed and calculated frequencies (in MHz) as well as residuals (in kHz) for the assigned transitions $J'_{K'_a,K'_c} \leftarrow J_{K_a,K_c}$ of the conformer aac. Due to the strong amplification power of the 300 W traveling wavetube amplifier, rotational transitions above 8 GHz were observed.

J'	K'_a	K'_c	J	K_a	K_c	Observed (MHz)	Calculated (MHz)	Obs–Calc (kHz)
1	1	0	0	0	0	2638.9542	2638.9587	-4.5
2	0	2	1	1	0	2356.0138	2356.0225	-8.7
2	0	2	1	0	1	3322.9764	3322.9706	5.8
2	1	2	1	0	1	3994.3491	3994.3368	12.3
2	1	2	1	1	1	3185.7046	3185.7041	0.5
2	1	1	1	1	0	3502.3204	3502.3337	-13.3
2	1	1	1	0	1	4469.2796	4469.2817	-2.1
2	2	1	2	1	2	2900.8365	2900.8416	-5.1
2	2	1	1	1	1	6086.5465	6086.5456	0.9
2	2	1	2	1	1	2425.8875	2425.8967	-9.2
2	2	1	1	1	0	5928.2297	5928.2303	-0.6
2	2	0	1	1	0	5949.2836	5949.2798	3.8
2	2	0	1	1	1	6107.5921	6107.5951	-3
2	2	0	2	1	2	2921.89	2921.891	-1
3	0	3	2	1	2	4262.8215	4262.8205	1
3	0	3	2	0	2	4934.1912	4934.1868	4.4
3	0	3	2	1	1	3787.8602	3787.8756	-15.4
3	1	3	2	0	2	5437.5359	5437.5354	0.5

Continued on next page

Table S1 – continued from previous page

J'	K'_a	K'_c	J	K_a	K_c	Observed (MHz)	Calculated (MHz)	Obs–Calc (kHz)
3	1	3	2	1	2	4766.164	4766.1691	-5.1
3	1	2	2	1	1	5239.3422	5239.3428	-0.6
3	1	2	2	0	2	6385.655	6385.654	1
3	2	2	3	1	2	2202.5759	2202.5738	2.1
3	2	2	3	1	3	3150.6989	3150.6923	6.6
3	2	2	2	1	1	7441.9216	7441.9166	5
3	2	2	2	1	2	7916.8621	7916.8615	0.6
3	2	1	3	1	3	3253.591	3253.582	9
3	2	1	3	1	2	2305.4648	2305.4634	1.4
3	2	1	2	1	1	7544.8082	7544.8062	2
3	3	1	3	2	1	4348.4299	4348.4398	-9.9
3	3	1	2	2	1	9467.3521	9467.3494	2.7
3	3	0	3	2	2	4453.109	4453.0976	11.4
3	3	0	2	2	0	9448.0801	9448.068	12.1
4	0	4	3	0	3	6496.6792	6496.6857	-6.5
4	0	4	3	1	3	5993.3388	5993.3371	1.7
4	0	4	3	1	2	5045.226	5045.2185	7.5
4	1	4	3	0	3	6837.4477	6837.4365	11.2
4	1	3	3	0	3	8408.3211	8408.3227	-1.6
4	1	3	3	2	1	4651.4028	4651.3921	10.7
4	2	3	3	2	2	6671.5859	6671.5736	12.3
4	2	3	3	1	3	9822.2548	9822.2659	-11.1
4	2	3	4	1	4	3488.1686	3488.1781	-9.5
4	2	2	4	1	3	2211.3902	2211.4029	-12.7

Continued on next page

Table S1 – continued from previous page

J'	K'_a	K'_c	J	K_a	K_c	Observed (MHz)	Calculated (MHz)	Obs–Calc (kHz)
4	2	2	3	2	1	6862.7897	6862.7949	-5.2
4	2	2	3	1	2	9168.2483	9168.2583	-10
4	2	2	4	1	4	3782.2917	3782.2891	2.6
4	3	2	4	2	2	4210.9675	4210.9684	-0.9
4	3	2	4	2	3	4505.0758	4505.0794	-3.6
4	3	1	4	2	3	4517.3284	4517.3306	-2.2
4	4	0	4	3	2	6181.3903	6181.3856	4.7
5	0	5	4	1	4	7678.0019	7677.9965	5.4
5	1	5	4	1	4	7888.6505	7888.6539	-3.4
5	1	5	4	2	3	4400.4743	4400.4758	-1.5
5	1	4	4	2	2	6433.038	6433.0368	1.2
5	1	4	5	0	5	2537.3249	2537.3293	-4.4
5	2	4	4	1	4	11801.4752	11801.4798	-4.6
5	2	4	4	3	2	3808.2246	3808.2223	2.3
5	2	3	5	1	5	4547.2494	4547.2413	8.1
5	2	3	5	1	4	2220.5629	2220.5694	-6.5
5	3	3	5	2	3	3973.0628	3973.0608	2
5	3	2	5	2	4	4655.487	4655.4895	-2.5
5	4	2	5	3	2	6124.2308	6124.247	-16.2
5	4	1	5	3	3	6173.3755	6173.3596	15.9
6	1	6	5	2	4	5518.0986	5518.1021	-3.5
6	2	5	5	3	3	5330.9358	5330.9452	-9.4
6	2	4	6	1	5	2376.4521	2376.457	-4.9
6	3	4	6	2	4	3632.4008	3632.3977	3.1

Continued on next page

Table S1 – continued from previous page

J'	K'_a	K'_c	J	K_a	K_c	Observed (MHz)	Calculated (MHz)	Obs–Calc (kHz)
6	3	3	6	2	5	4913.0023	4912.9987	3.6
6	4	3	6	3	3	6030.5081	6030.5097	-1.6
6	4	2	6	3	4	6174.7857	6174.7846	1.1
7	3	5	7	2	5	3204.3512	3204.3525	-1.3
7	3	4	7	2	6	5344.4925	5344.4973	-4.8
7	4	4	7	3	4	5857.448	5857.4432	4.8
7	4	3	7	3	5	6203.7099	6203.7143	-4.4
8	4	4	8	3	6	6288.9748	6288.9818	-7
8	4	4	7	5	2	5660.1621	5660.158	4.1
9	3	7	9	2	7	2192.8346	2192.8326	2
9	4	5	9	3	7	6471.6828	6471.6697	13.1
10	4	7	10	3	8	6494.1341	6494.1392	-5.1

Table S2: Observed and calculated frequencies (in MHz) as well as residuals (in kHz) for the assigned transitions $J'_{K'_a,K'_c} \leftarrow J_{K_a,K_c}$ of the conformer g-ac.

J'	K'_a	K'_c	J	K_a	K_c	Observed (MHz)	Calculated (MHz)	Obs–Calc (kHz)
1	1	0	0	0	0	2813.7346	2813.7434	-8.8
2	0	2	1	0	1	2872.4918	2872.4887	3.1
2	1	1	1	0	1	4313.3229	4313.3266	-3.7
2	2	1	1	1	1	6941.6369	6941.6426	-5.7
2	2	0	2	1	2	4131.4091	4131.4137	-4.6
2	2	0	1	1	0	6881.5491	6881.5429	6.2
3	0	3	2	1	1	2862.5107	2862.5068	3.9
3	0	3	2	0	2	4303.3465	4303.3446	1.9
3	1	2	2	0	2	5844.8204	5844.8213	-0.9
3	1	2	2	1	1	4403.97	4403.9835	-13.5
4	0	4	3	1	2	4186.3536	4186.3576	-4
4	1	4	3	1	3	5620.5819	5620.578	3.9
4	1	3	3	0	3	7410.8195	7410.8129	6.6
4	1	3	3	1	2	5869.3424	5869.3363	6.1
4	2	3	4	1	3	3728.7221	3728.7224	-0.3
4	2	2	4	1	4	4383.2228	4383.222	0.8
5	2	4	5	1	4	3578.2308	3578.2257	5.1
6	0	6	5	1	4	6679.8728	6679.8754	-2.6

Table S3: Observed and calculated frequencies (in MHz) as well as residuals (in kHz) for the assigned transitions $J'_{K'_a,K'_c} \leftarrow J_{K_a,K_c}$ of the conformer g+ac.

J'	K'_a	K'_c	J	K_a	K_c	Observed (MHz)	Calculated (MHz)	Obs–Calc (kHz)
1	1	0	0	0	0	2720.8445	2720.8531	-8.6
2	1	2	1	0	1	4138.2265	4138.2232	3.3
2	1	1	1	0	1	4351.3037	4351.3137	-10
2	2	0	2	1	2	3487.5998	3487.6023	-2.5
2	2	0	1	1	0	6464.3984	6464.409	-10.6
3	0	3	2	1	2	3642.2131	3642.1967	16.4
3	0	3	2	1	1	3429.0994	3429.1062	-6.8
3	1	2	2	0	2	6018.4916	6018.4877	3.9
3	2	2	3	1	2	3166.7616	3166.7506	11
3	2	1	3	1	3	3609.5825	3609.5743	8.2
3	2	1	2	1	1	7966.1856	7966.1771	8.5
4	1	4	3	0	3	7016.711	7016.7088	2.2
4	1	3	3	0	3	7726.3089	7726.3158	-6.9
4	2	3	4	1	3	3029.0898	3029.0891	0.7
5	2	4	5	1	4	2859.7947	2859.8012	-6.5
6	1	5	5	2	3	6565.1706	6565.1713	-0.7
6	3	4	6	2	4	5464.545	5464.5455	-0.5

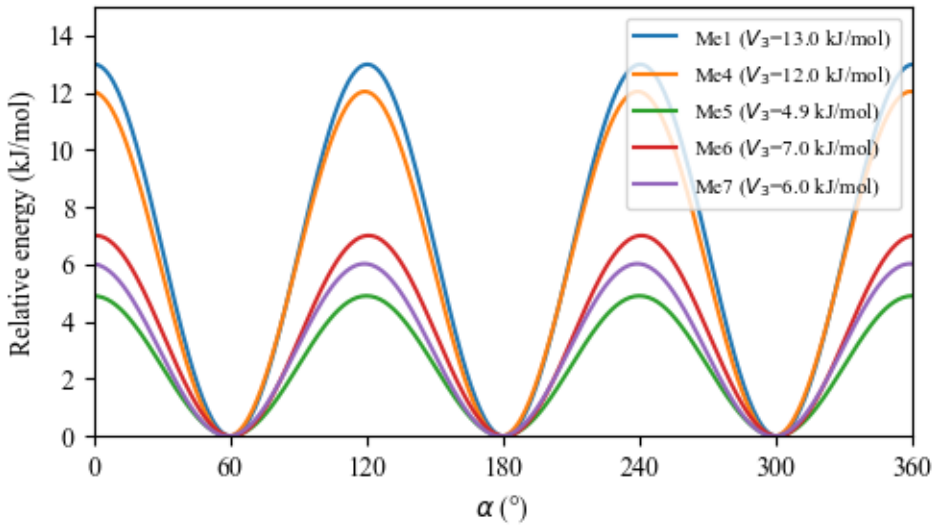

Figure S7: Scan of the torsional angle α (H-C-Si-O) to determine the internal rotational barriers V_3 for the different methyl rotors MeX. Due to the C_{3v} symmetry of a methyl rotor, the angle α was rotated for 120° in steps of 10° and the energy calculated at B3LYP D3(BJ)/def2-TZVP level of theory. The points were fit with a threefold torsional potential $V(\alpha) = \frac{V_3}{2} \cdot (1 - \cos(3 \cdot (\alpha - \phi)))$ and the values for V_3 obtained.

The prediction in Figure S8 is based on the experimentally determined V_3 barriers obtained from the Me5-Me6 fit (5.54 and 8.39 kJ mol^{-1}). Regarding Me7, although DFT calculations suggest a barrier of 6 kJ/mol, we found that this value did not accurately reproduce the observed splitting patterns. We therefore adopted a slightly higher barrier for Me7 while still maintaining the general DFT trend of $V_3(Me5) < V_3(Me7) < V_3(Me6)$, as this provided a better match with the experimental data. The 14 different states are labelled according to their symmetry indices $\sigma_5\sigma_7\sigma_6$ referring to the methyl rotor Me5, Me7, and Me6, respectively. All states with $\sigma_5 = 0$ are close to the transition of the semi-rigid rotor.

Figure S9 shows predictions based on the different effective two-top fit combinations exemplified for the transition $J'_{K'_aK'_c} \leftarrow J_{K_aK_c} = 3_{31} \leftarrow 2_{21}$. The combination Me5-Me6 can reproduce the observed splitting pattern the best, while for the combinations Me5-Me7 and Me6-Me7 the splitting of the triplet pattern seems too narrow.

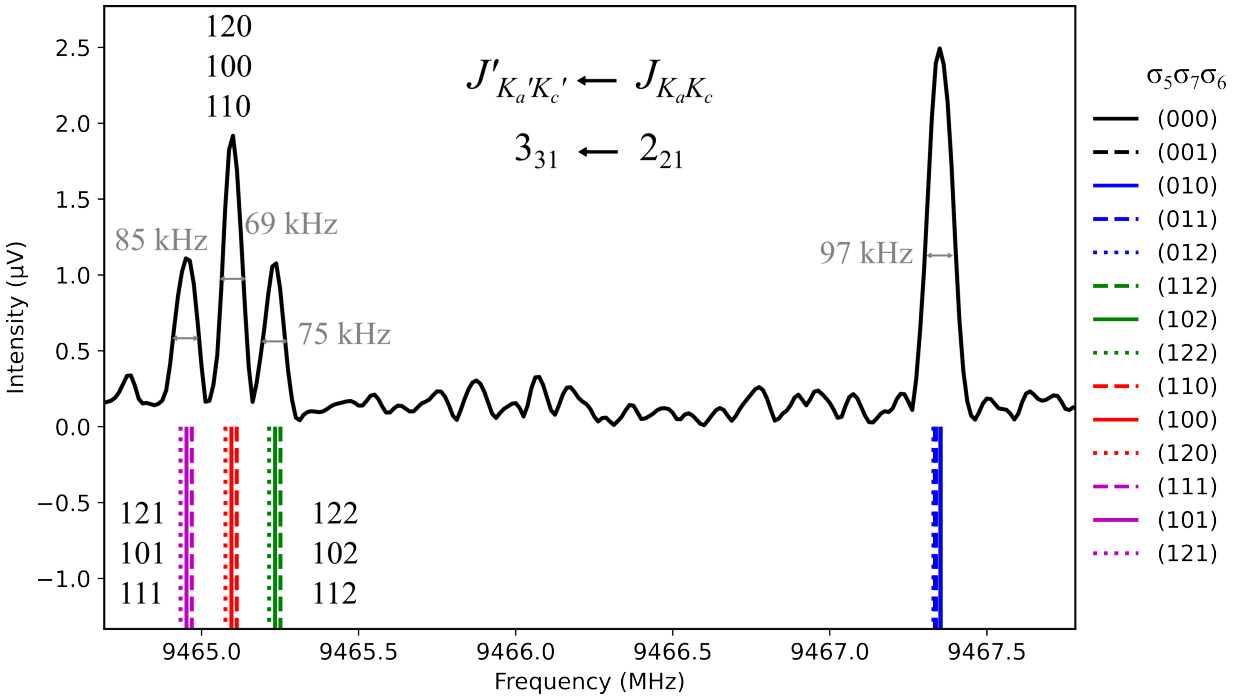

Figure S8: Prediction for three methyl rotors Me5-Me7-Me6 with the barriers 5.54, 7.39, and 8.39 kJ mol^{-1}, respectively, including 14 symmetry species labelled according to the symmetry indices $\sigma_5\sigma_7\sigma_6$ referring to rotor Me5, Me7, and Me6, respectively. The full width half maximum values for the lines have been added in kHz indicating larger line width. On the left, the symmetry labels have been added to the triplet pattern describing the species from left to right.

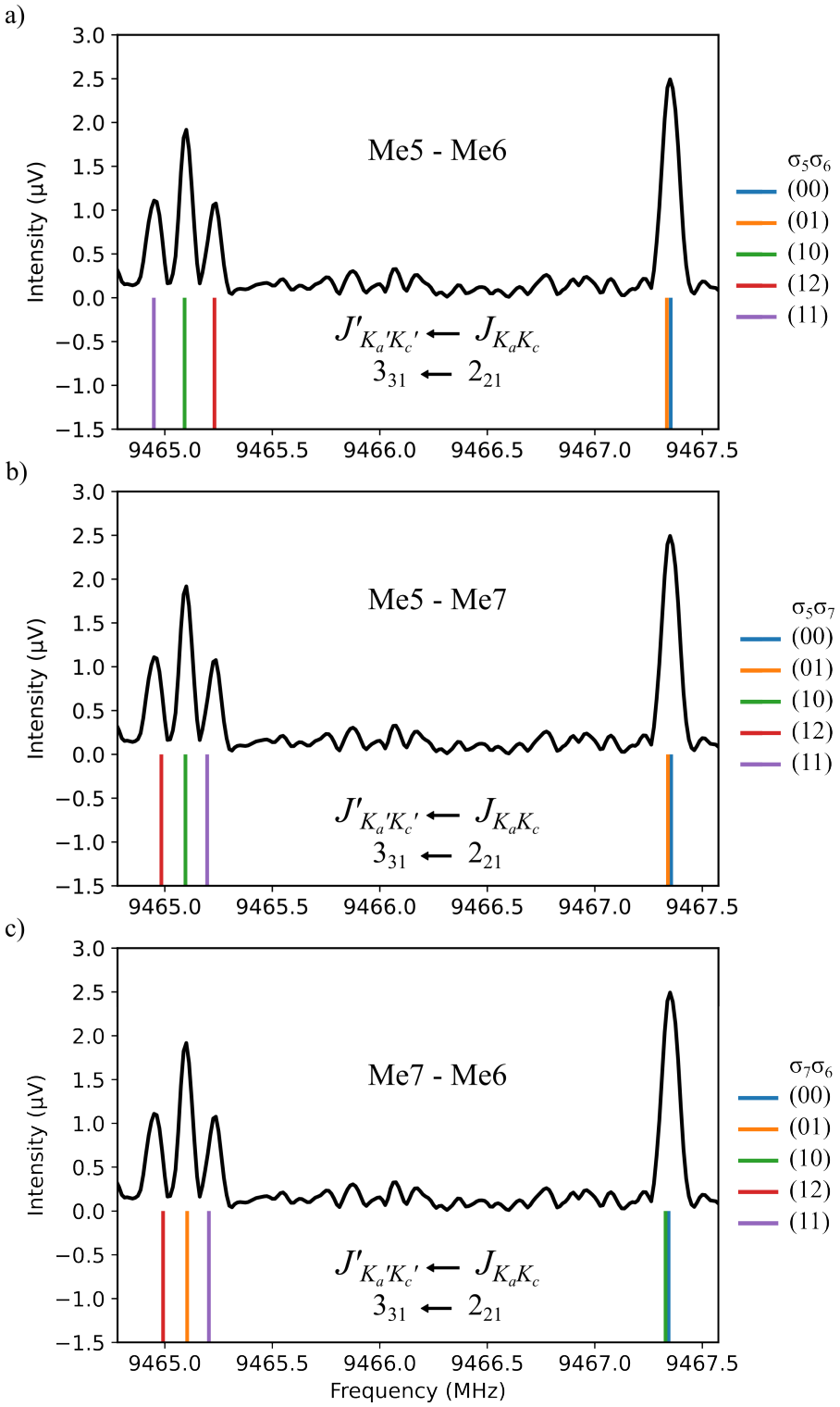

Figure S9: Predictions based on the effective two-top fits for the different methyl rotors combinations a) Me5-Me6 (5.54 kJ mol^{-1} and 8.39 kJ mol^{-1}, σ_{rms} = 9.5 kHz), b) Me5-Me7 (5.54 kJ mol^{-1} and 5.45 kJ mol^{-1}, σ_{rms} = 27.5 kHz), and c) Me7-Me6 (5.47 kJ mol^{-1} and 5.13 kJ mol^{-1}, σ_{rms} = 45.7 kHz).

```
 Rotational, Centrifugal Distortion, Internal Rotation Calculation (V2.5e)
                       Holger Hartwig 08-Nov-96 (hartwig@phc.uni-kiel.de)

 Please cite: H.Hartwig and H.Dreizler, Z.Naturforsch, 51a (1996) 923.

 Calculation date and time:
 Modified version to include Dc3- and Dc3K

 Type help now for the list of parameters :
sBT-Si Me5Me6

   nzyk       200   print        4   eval         0   dfreq        0
   orger        0   ints         0   maxm         8   woods       33
   ndata     5000   nfold        3   spin         0   ntop         2
   adjf        16   maxvm        0   aprint      10   xprint       4
   ncycl      200   svderr       0   fitscl       0   reduct       0
   rofit    .0000000D+00   eps      .1000000D-11   defer    .1000000D-04
   weigf    .0000000D+00   convg    .1000000D+01   lambda   .1000000D-04
   freq_l   .6000000D+01   freq_h   .4000000D+02   limit    .1000000D+00
   temp     .2730000D+03
 Using Watson A Reduction

 assumed sizeb           1
 \\ set (adj  or  8)
 \\ set (adj  or  1)
 \\ set (adj  or  2)
 \\ adj  1: adjust F according to rho, beta and gamma
 \\ adj  2: adjust F12 according to rho, beta and gamma
 \\ adj  8: adjust rho according to F0 = 1/(2 I_alpha)
 \\ adj 16: adjust beta and gamma  according delta + epsil
 new adj :  27
 BJ            0.836004161
 BK            0.887768168
 B-            0.079157659
 DJ            0.052360E-6
 DJK           0.292358E-6
 DK           -0.115072E-6
 dj            0.021923E-6
 dk           -0.581357E-6
\F12           0.810818494
\F           161.464798971                 161.299616186
 V1n          12395.069220                  17621.743930
\rho           0.007126178                   0.006458854
\beta          0.600691769                   0.913402443
\gamma         1.215427384                   0.426131191
 F0          160.398000000                 160.297000000
 epsil         1.273000000                   0.502000000
 delta         0.992000000                   1.195000000

 fit  0.1D+00 0.1D+01   BJ          1.00
 fit  0.1D+00 0.1D+01   BK          1.00
 fit  0.1D+00 0.1D+01   B-          1.00
 fit  0.1D+00 0.1D+01   V1n_1       1.00
 fit  0.1D+00 0.1D+01   DJ          1.00
 fit  0.1D+00 0.1D+01   DJK         1.00
 fit  0.1D+00 0.1D+01   DK          1.00
 fit  0.1D+00 0.1D+01   dj          1.00
 fit  0.1D+00 0.1D+01   dk          1.00
 fit  0.1D+00 0.1D+01   V1n_2       1.00

  S     0   0
  S     0   1
  S     1   0
  S    -1   1
  S     1   1

  V  0  0

   ndata  207   Data Points 207   Splittings   0
   Effective Data Points  92.0

  \\  Maximal K = J =  10
  \\ B=  1    adj= 27
```

```
 \\ (1) calculate torsional integrals
 \\ (32)use torsional integrals in rigid rotor H_rr
Sigma:0.103862D-02  Sigma/OldSigma:0.000000  conv: 1

      J K- K+  J K- K+    Sym   calc/GHz  diff/MHz     obs/GHz
  1:  9  3  7  9  2  7   S 1   2.1928139    0.0246   2.1928384   Err
  0.1D-04              -
  2:  9  3  7  9  2  7   S 2   2.1928349    0.0036   2.1928384   Err
  0.1D-04              -
  3:  9 K   3  9 K   0   S 3   2.1926258    0.0759   2.1927017   Err
  0.1D-04              -
  4:  9 K  -3  9 K   0   S 4   2.1926459    0.0559   2.1927017   Err
  0.1D-04              -
  5:  9 K   3  9 K   0   S 5   2.1926477    0.0540   2.1927017   Err
  0.1D-04              -
  6:  3  2  2  3  1  2   S 1   2.2025457    0.0276   2.2025733   Err
  0.1D-04              -
  7:  3  2  2  3  1  2   S 2   2.2025478    0.0255   2.2025733   Err
  0.1D-04              -
  8:  3 K   2  3 K  -1   S 3   2.2023386    0.1566   2.2024952   Err
  0.1D-04              -
  9:  3 K  -2  3 K   1   S 4   2.2023670    0.1282   2.2024952   Err
  0.1D-04              -
 10:  3 K   2  3 K  -1   S 5   2.2023144    0.1808   2.2024952   Err
 0.1D-04               -
 11:  4  2  2  4  1  3   S 1   2.2113733    0.0171   2.2113904   Err
 0.1D-04               -
 12:  5  2  3  5  1  4   S 1   2.2205383    0.0247   2.2205630   Err
 0.1D-04               -
 13:  3  2  1  3  1  2   S 1   2.3054330    0.0320   2.3054650   Err
 0.1D-04               -
 14:  2  0  2  1  1  0   S 1   2.3560472   -0.0337   2.3560135   Err
 0.1D-04               -
 15:  6  2  4  6  1  5   S 1   2.3764212    0.0309   2.3764520   Err
 0.1D-04               -
 16:  2  2  1  2  1  1   S 1   2.4258646    0.0236   2.4258882   Err
 0.1D-04               -
 17:  5  1  4  5  0  5   S 1   2.5372858    0.0397   2.5373256   Err
 0.1D-04               -
 18:  1  1  0  0  0  0   S 1   2.6389504    0.0038   2.6389542   Err
 0.1D-04               -
 19:  2  2  1  2  1  2   S 1   2.9008008    0.0357   2.9008365   Err
 0.1D-04               -
 20:  2  2  0  2  1  2   S 1   2.9218498    0.0396   2.9218894   Err
 0.1D-04               -
 21:  2  2  0  2  1  2   S 2   2.9218528    0.0367   2.9218894   Err
 0.1D-04               -
 22:  2 K  -2  2 K   1   S 3   2.9224917   -0.3979   2.9220938   Err
 0.1D-04               -
 23:  2 K   2  2 K  -1   S 4   2.9223714   -0.3350   2.9220364   Err
 0.1D-04               -
 24:  2 K  -2  2 K   1   S 5   2.9226159   -0.4905   2.9221255   Err
 0.1D-04               -
 25:  3  2  2  3  1  3   S 1   3.1506470    0.0490   3.1506960   Err
 0.1D-04               -
 26:  3  2  2  3  1  3   S 2   3.1506407    0.0554   3.1506960   Err
 0.1D-04               -
 27:  3 K   2  3 K   1   S 3   3.1504687    0.1601   3.1506288   Err
 0.1D-04               -
 28:  3 K  -2  3 K  -1   S 4   3.1504903    0.1385   3.1506288   Err
 0.1D-04               -
 29:  3 K   2  3 K   1   S 5   3.1504344    0.1944   3.1506288   Err
 0.1D-04               -
 30:  4  1  4  3  2  2   S 1   3.1834686   -0.0410   3.1834276   Err
 0.1D-04               -
 31:  2  1  2  1  1  1   S 1   3.1857176   -0.0123   3.1857052   Err
 0.1D-04               -
 32:  7  3  5  7  2  5   S 1   3.2043165    0.0342   3.2043507   Err
 0.1D-04               -
 33:  7  3  5  7  2  5   S 2   3.2043298    0.0209   3.2043507   Err
 0.1D-04               -
 34:  7 K   3  7 K  -2   S 3   3.2040624    0.1629   3.2042252   Err
 0.1D-04               -
```

```
113     35:  7 K  -3  7 K   2   S 4   3.2040927    0.1325   3.2042252   Err
        0.1D-04              -
114     36:  7 K   3  7 K  -2   S 5   3.2040586    0.1666   3.2042252   Err
        0.1D-04              -
115     37:  3  2  1  3  1  3   S 1   3.2535343    0.0361   3.2535704   Err
        0.1D-04              -
116     38:  3  2  1  3  1  3   S 2   3.2535287    0.0417   3.2535704   Err
        0.1D-04              -
117     39:  3 K  -2  3 K   1   S 3   3.2536439   -0.0310   3.2536129   Err
        0.1D-04              -
118     40:  3 K   2  3 K  -1   S 4   3.2536146   -0.0017   3.2536129   Err
        0.1D-04              -
119     41:  3 K  -2  3 K   1   S 5   3.2536620   -0.0491   3.2536129   Err
        0.1D-04              -
120     42:  2  0  2  1  0  1   S 1   3.3229816   -0.0149   3.3229667   Err
        0.1D-04              -
121     43:  4  2  3  4  1  4   S 1   3.4881265    0.0312   3.4881577   Err
        0.1D-04              -
122     44:  2  1  1  1  1  0   S 1   3.5023414   -0.0205   3.5023209   Err
        0.1D-04              -
123     45:  6  3  4  6  2  4   S 1   3.6323537    0.0469   3.6324006   Err
        0.1D-04              -
124     46:  6  3  4  6  2  4   S 2   3.6323616    0.0391   3.6324006   Err
        0.1D-04              -
125     47:  6 K   3  6 K  -2   S 3   3.6319794    0.2692   3.6322486   Err
        0.1D-04              -
126     48:  6 K  -3  6 K   2   S 4   3.6320309    0.2178   3.6322486   Err
        0.1D-04              -
127     49:  6 K   3  6 K  -2   S 5   3.6319437    0.3049   3.6322486   Err
        0.1D-04              -
128     50:  4  2  2  4  1  4   S 1   3.7822310    0.0608   3.7822917   Err
        0.1D-04              -
129     51:  3  0  3  2  1  1   S 1   3.7879103   -0.0150   3.7878952   Err
        0.1D-04              -
130     52:  3  0  3  2  1  1   S 2   3.7879075   -0.0123   3.7878952   Err
        0.1D-04              -
131     53:  3 K   0  2 K  -1   S 3   3.7878452    0.0005   3.7878458   Err
        0.1D-04              -
132     54:  3 K   0  2 K   1   S 4   3.7878482   -0.0024   3.7878458   Err
        0.1D-04              -
133     55:  3 K   0  2 K  -1   S 5   3.7878368    0.0090   3.7878458   Err
        0.1D-04              -
134     56:  5  2  4  4  3  2   S 1   3.8083119   -0.0871   3.8082249   Err
        0.1D-04              -
135     57:  5  3  3  5  2  3   S 1   3.9730101    0.0526   3.9730628   Err
        0.1D-04              -
136     58:  5  3  3  5  2  3   S 2   3.9730100    0.0528   3.9730628   Err
        0.1D-04              -
137     59:  5 K   3  5 K  -2   S 3   3.9722091    0.5686   3.9727778   Err
        0.1D-04              -
138     60:  5 K  -3  5 K   2   S 4   3.9723349    0.5006   3.9728356   Err
        0.1D-04              -
139     61:  5 K   3  5 K  -2   S 5   3.9720840    0.6615   3.9727455   Err
        0.1D-04              -
140     62:  2  1  2  1  0  1   S 1   3.9943396    0.0030   3.9943426   Err
        0.1D-04              -
141     63:  2  1  2  1  0  1   S 2   3.9943373    0.0053   3.9943426   Err
        0.1D-04              -
142     64:  2 K   1  1 K   0   S 3   3.9942951    0.0042   3.9942993   Err
        0.1D-04              -
143     65:  2 K  -1  1 K   0   S 4   3.9942927    0.0066   3.9942993   Err
        0.1D-04              -
144     66:  2 K   1  1 K   0   S 5   3.9942928    0.0065   3.9942993   Err
        0.1D-04              -
145     67:  4  3  2  4  2  2   S 1   4.2109128    0.0546   4.2109674   Err
        0.1D-04              -
146     68:  4  3  2  4  2  2   S 2   4.2108919    0.0756   4.2109674   Err
        0.1D-04              -
147     69:  4 K   3  4 K  -2   S 3   4.2085067    1.6208   4.2101275   Err
        0.1D-04              -
148     70:  4 K  -3  4 K   2   S 4   4.2088721    1.3390   4.2102111   Err
        0.1D-04              -
149     71:  4 K   3  4 K  -2   S 5   4.2081276    1.9174   4.2100449   Err
```

```
      0.1D-04             -
150   72:  3  0  3  2  1  2   S 1   4.2628465   -0.0248   4.2628217   Err
      0.1D-04             -
151   73:  3  3  1  3  2  1   S 1   4.3483812    0.0800   4.3484612   Err
      0.1D-04             -
152   74:  3  3  1  3  2  1   S 2   4.3482312    0.1920   4.3484232   Err
      0.1D-04             -
153   75:  3 K   3  3 K  -2   S 3   4.3432209    2.7223   4.3459431   Err
      0.1D-04             -
154   76:  3 K  -3  3 K   2   S 4   4.3437744    2.3363   4.3461107   Err
      0.1D-04             -
155   77:  3 K   3  3 K  -2   S 5   4.3426648    3.1023   4.3457671   Err
      0.1D-04             -
156   78:  3 K  -3  3 K  -2   S 3   4.3548562   -2.3289   4.3525273   Err
      0.1D-04             -
157   79:  3 K   3  3 K   2   S 4   4.3543531   -1.9806   4.3523726   Err
      0.1D-04             -
158   80:  3 K  -3  3 K  -2   S 5   4.3553589   -2.6824   4.3526765   Err
      0.1D-04             -
159   81:  5  1  5  4  2  3   S 1   4.4005627   -0.0885   4.4004742   Err
      0.1D-04             -
160   82:  3  3  0  3  2  2   S 1   4.4530366    0.0731   4.4531097   Err
      0.1D-04             -
161   83:  3  3  0  3  2  2   S 2   4.4531858   -0.0761   4.4531097   Err
      0.1D-04             -
162   84:  3 K  -3  3 K   2   S 3   4.4580314   -2.5206   4.4555108   Err
      0.1D-04             -
163   85:  3 K   3  3 K  -2   S 4   4.4574774   -2.1327   4.4553448   Err
      0.1D-04             -
164   86:  3 K  -3  3 K   2   S 5   4.4585864   -2.9116   4.4556749   Err
      0.1D-04             -
165   87:  2  1  1  1  0  1   S 1   4.4692759    0.0040   4.4692799   Err
      0.1D-04             -
166   88:  4  3  2  4  2  3   S 1   4.5050173    0.0580   4.5050753   Err
      0.1D-04             -
167   89:  4  3  2  4  2  3   S 2   4.5049924    0.0829   4.5050753   Err
      0.1D-04             -
168   90:  4 K   3  4 K   2   S 3   4.5027307    1.5515   4.5042822   Err
      0.1D-04             -
169   91:  4 K  -3  4 K  -2   S 4   4.5030750    1.2856   4.5043606   Err
      0.1D-04             -
170   92:  4 K   3  4 K   2   S 5   4.5023647    1.8420   4.5042067   Err
      0.1D-04             -
171   93:  4  3  1  4  2  3   S 1   4.5172681    0.0598   4.5173279   Err
      0.1D-04             -
172   94:  4  3  1  4  2  3   S 2   4.5172892    0.0388   4.5173279   Err
      0.1D-04             -
173   95:  4 K  -3  4 K   2   S 3   4.5195018   -1.4304   4.5180713   Err
      0.1D-04             -
174   96:  4 K   3  4 K  -2   S 4   4.5191371   -1.1502   4.5179869   Err
      0.1D-04             -
175   97:  4 K  -3  4 K   2   S 5   4.5198805   -1.7330   4.5181475   Err
      0.1D-04             -
176   98:  5  2  3  5  1  5   S 1   4.5471682    0.0808   4.5472490   Err
      0.1D-04             -
177   99:  4  1  3  3  2  1   S 1   4.6514390   -0.0284   4.6514106   Err
      0.1D-04             -
178  100:  4  1  3  3  2  1   S 2   4.6514256   -0.0150   4.6514106   Err
     0.1D-04              -
179  101:  4 K  -1  3 K  -2   S 3   4.6513245    0.0340   4.6513585   Err
     0.1D-04              -
180  102:  4 K   1  3 K   2   S 4   4.6513374    0.0211   4.6513585   Err
     0.1D-04              -
181  103:  4 K  -1  3 K  -2   S 5   4.6512848    0.0737   4.6513585   Err
     0.1D-04              -
182  104:  5  3  2  5  2  4   S 1   4.6554238    0.0628   4.6554866   Err
     0.1D-04              -
183  105:  5  3  2  5  2  4   S 2   4.6554248    0.0618   4.6554866   Err
     0.1D-04              -
184  106:  5 K  -3  5 K   2   S 3   4.6560462   -0.3807   4.6556654   Err
     0.1D-04              -
185  107:  5 K   3  5 K  -2   S 4   4.6559223   -0.3059   4.6556164   Err
     0.1D-04              -
```

```
186    108:  5 K  -3  5 K   2   S 5   4.6561711   -0.4697   4.6557014   Err
       0.1D-04                -
187    109:  3  1  3  2  1  2   S 1   4.7661896   -0.0040   4.7661856   Err
       0.1D-04                -
188    110:  3  1  3  2  1  2   S 2   4.7661856   -0.0000   4.7661856   Err
       0.1D-04                -
189    111:  3 K   1  2 K   1   S 3   4.7661664   -0.0152   4.7661512   Err
       0.1D-04                -
190    112:  3 K  -1  2 K  -1   S 4   4.7661596   -0.0084   4.7661512   Err
       0.1D-04                -
191    113:  3 K   1  2 K   1   S 5   4.7661653   -0.0141   4.7661512   Err
       0.1D-04                -
192    114:  6  3  3  6  2  5   S 1   4.9129269    0.0698   4.9129967   Err
       0.1D-04                -
193    115:  3  0  3  2  0  2   S 1   4.9342045   -0.0119   4.9341926   Err
       0.1D-04                -
194    116:  3  0  3  2  0  2   S 2   4.9341993   -0.0067   4.9341926   Err
       0.1D-04                -
195    117:  3 K   0  2 K   0   S 3   4.9341524    0.0043   4.9341567   Err
       0.1D-04                -
196    118:  3 K   0  2 K   0   S 4   4.9341497    0.0070   4.9341567   Err
       0.1D-04                -
197    119:  3 K   0  2 K   0   S 5   4.9341448    0.0119   4.9341567   Err
       0.1D-04                -
198    120:  4  0  4  3  1  2   S 1   5.0452667   -0.0302   5.0452365   Err
       0.1D-04                -
199    121:  3  1  2  2  1  1   S 1   5.2393547   -0.0129   5.2393418   Err
       0.1D-04                -
200    122:  6  2  5  5  3  3   S 1   5.3310435   -0.1217   5.3309218   Err
       0.1D-04                -
201    123:  7  3  4  7  2  6   S 1   5.3444156    0.0733   5.3444889   Err
       0.1D-04                -
202    124:  3  1  3  2  0  2   S 1   5.4375476    0.0049   5.4375524   Err
       0.1D-04                -
203    125:  3  1  3  2  0  2   S 2   5.4375452    0.0072   5.4375524   Err
       0.1D-04                -
204    126:  3 K   1  2 K   0   S 3   5.4375072    0.0044   5.4375116   Err
       0.1D-04                -
205    127:  3 K  -1  2 K   0   S 4   5.4375008    0.0108   5.4375116   Err
       0.1D-04                -
206    128:  3 K   1  2 K   0   S 5   5.4375088    0.0028   5.4375116   Err
       0.1D-04                -
207    129:  6  1  6  5  2  4   S 1   5.5182046   -0.1061   5.5180985   Err
       0.1D-04                -
208    130:  8  4  4  7  5  2   S 1   5.6603028   -0.1406   5.6601621   Err
       0.1D-04                -
209    131:  7  4  4  7  3  4   S 1   5.8573670    0.0814   5.8574485   Err
       0.1D-04                -
210    132:  7  4  4  7  3  4   S 2   5.8573468    0.1017   5.8574485   Err
       0.1D-04                -
211    133:  7 K   4  7 K  -3   S 3   5.8545330    1.9469   5.8564799   Err
       0.1D-04                -
212    134:  2  2  1  1  1  0   S 1   5.9282059    0.0241   5.9282300   Err
       0.1D-04                -
213    135:  2  2  0  1  1  0   S 1   5.9492549    0.0278   5.9492827   Err
       0.1D-04                -
214    136:  2  2  0  1  1  0   S 2   5.9492562    0.0266   5.9492827   Err
       0.1D-04                -
215    137:  2 K   2  1 K   1   S 4   5.9497217   -0.3101   5.9494115   Err
       0.1D-04                -
216    138:  2 K  -2  1 K  -1   S 5   5.9499563   -0.4772   5.9494790   Err
       0.1D-04                -
217    139:  4  0  4  3  1  3   S 1   5.9933680   -0.0286   5.9933394   Err
       0.1D-04                -
218    140:  6  4  3  6  3  3   S 1   6.0304293    0.0784   6.0305077   Err
       0.1D-04                -
219    141:  2  2  1  1  1  1   S 1   6.0865184    0.0275   6.0865458   Err
       0.1D-04                -
220    142:  2  2  1  1  1  1   S 2   6.0865069    0.0389   6.0865458   Err
       0.1D-04                -
221    143:  2 K  -2  1 K  -1   S 4   6.0859107    0.4219   6.0863326   Err
       0.1D-04                -
222    144:  2 K   2  1 K   1   S 5   6.0856758    0.5892   6.0862650   Err
```

```
      0.1D-04                -
223   145:  2  2  0  1  1  1   S 1   6.1075674    0.0218   6.1075891   Err
      0.1D-04                -
224   146:  5  4  2  5  3  2   S 1   6.1241643    0.0666   6.1242308   Err
      0.1D-04                -
225   147:  5  4  2  5  3  3   S 2   6.1718314    0.3808   6.1722122   Err
      0.1D-04                -
226   148:  4 K  -4  4 K  -3   S 3   6.1742842   -1.8001   6.1724841   Err
      0.1D-04                -
227   149:  4 K   4  4 K   3   S 4   6.1739242   -1.6060   6.1723183   Err
      0.1D-04                -
228   150:  4 K  -4  4 K  -3   S 5   6.1746247   -1.9898   6.1726350   Err
      0.1D-04                -
229   151:  5  4  1  5  3  3   S 1   6.1732755    0.0970   6.1733726   Err
      0.1D-04                -
230   152:  5  4  1  5  3  3   S 2   6.1736302   -0.2577   6.1733726   Err
      0.1D-04                -
231   153:  5 K  -4  5 K   3   S 3   6.1809448   -3.7369   6.1772079   Err
      0.1D-04                -
232   154:  5 K   4  5 K  -3   S 4   6.1801180   -3.1461   6.1769719   Err
      0.1D-04                -
233   155:  5 K  -4  5 K   3   S 5   6.1817809   -4.3146   6.1774663   Err
      0.1D-04                -
234   156:  6  4  2  6  3  4   S 1   6.1747003    0.0854   6.1747857   Err
      0.1D-04                -
235   157:  6  4  2  6  3  4   S 2   6.1747920   -0.0062   6.1747857   Err
      0.1D-04                -
236   158:  6 K  -4  6 K   3   S 3   6.1801858   -3.1034   6.1770824   Err
      0.1D-04                -
237   159:  6 K   4  6 K  -3   S 4   6.1794811   -2.5832   6.1768979   Err
      0.1D-04                -
238   160:  6 K  -4  6 K   3   S 5   6.1808989   -3.6257   6.1772732   Err
      0.1D-04                -
239   161:  4 K   4  4 K   3   S 3   6.1757168    2.0862   6.1778030   Err
      0.1D-04                -
240   162:  4 K  -4  4 K  -3   S 4   6.1760754    1.8710   6.1779464   Err
      0.1D-04                -
241   163:  4 K   4  4 K   3   S 5   6.1753746    2.2784   6.1776531   Err
      0.1D-04                -
242   164:  4  4  0  4  3  2   S 1   6.1813015    0.0886   6.1813901   Err
      0.1D-04                -
243   165:  4  4  0  4  3  2   S 2   6.1819786   -0.4203   6.1815584   Err
      0.1D-04                -
244   166:  7  4  3  7  3  5   S 1   6.2036290    0.0804   6.2037094   Err
      0.1D-04                -
245   167:  7  4  3  7  3  5   S 2   6.2036526    0.0568   6.2037094   Err
      0.1D-04                -
246   168:  7 K  -4  7 K   3   S 3   6.2062031   -1.6566   6.2045465   Err
      0.1D-04                -
247   169:  8  4  4  8  3  6   S 1   6.2888939    0.0810   6.2889749   Err
      0.1D-04                -
248   170:  3  1  2  2  0  2   S 1   6.3856489    0.0057   6.3856546   Err
      0.1D-04                -
249   171:  5  1  4  4  2  2   S 1   6.4330882   -0.0511   6.4330371   Err
      0.1D-04                -
250   172:  9  4  5  9  3  7   S 1   6.4715764    0.1069   6.4716833   Err
      0.1D-04                -
251   173: 10  4  7 10  3  8   S 1   6.4940461    0.0895   6.4941356   Err
      0.1D-04                -
252   174:  4  0  4  3  0  3   S 1   6.4967111   -0.0318   6.4966793   Err
      0.1D-04                -
253   175:  4  2  3  3  2  2   S 1   6.6715951   -0.0091   6.6715860   Err
      0.1D-04                -
254   176:  4  1  4  3  0  3   S 1   6.8374587   -0.0102   6.8374485   Err
      0.1D-04                -
255   177:  4  2  2  3  2  1   S 1   6.8628123   -0.0220   6.8627903   Err
      0.1D-04                -
256   178:  3  2  2  2  1  1   S 1   7.4419004    0.0230   7.4419234   Err
      0.1D-04                -
257   179:  3  2  2  2  1  1   S 2   7.4418941    0.0293   7.4419234   Err
      0.1D-04                -
258   180:  3 K   2  2 K  -1   S 3   7.4416687    0.1592   7.4418278   Err
      0.1D-04                -
```

```
259    181:  3 K  -2  2 K   1   S 4   7.4416897    0.1382   7.4418278   Err
       0.1D-04             -
260    182:  3 K   2  2 K  -1   S 5   7.4416352    0.1927   7.4418278   Err
       0.1D-04             -
261    183:  3  2  1  2  1  1   S 1   7.5447877    0.0206   7.5448083   Err
       0.1D-04             -
262    184:  5  0  5  4  1  4   S 1   7.6780333   -0.0318   7.6780016   Err
       0.1D-04             -
263    185:  5  0  5  4  1  4   S 2   7.6780234   -0.0218   7.6780016   Err
       0.1D-04             -
264    186:  5 K   0  4 K   1   S 3   7.6777700    0.1295   7.6778995   Err
       0.1D-04             -
265    187:  5 K   0  4 K  -1   S 4   7.6777933    0.1062   7.6778995   Err
       0.1D-04             -
266    188:  5 K   0  4 K   1   S 5   7.6777269    0.1726   7.6778995   Err
       0.1D-04             -
267    189:  5  1  5  4  1  4   S 1   7.8886892   -0.0390   7.8886502   Err
       0.1D-04             -
268    190:  3  2  2  2  1  2   S 1   7.9168366    0.0290   7.9168655   Err
       0.1D-04             -
269    191:  3  2  2  2  1  2   S 2   7.9168263    0.0393   7.9168655   Err
       0.1D-04             -
270    192:  3 K   2  2 K   1   S 3   7.9166351    0.1587   7.9167938   Err
       0.1D-04             -
271    193:  3 K  -2  2 K  -1   S 4   7.9166499    0.1439   7.9167938   Err
       0.1D-04             -
272    194:  3 K   2  2 K   1   S 5   7.9165997    0.1942   7.9167938   Err
       0.1D-04             -
273    195:  4  1  3  3  0  3   S 1   8.4083164    0.0051   8.4083214   Err
       0.1D-04             -
274    196:  4  2  2  3  1  2   S 1   9.1682453    0.0031   9.1682484   Err
       0.1D-04             -
275    197:  3  3  0  2  2  0   S 1   9.4480233    0.0600   9.4480833   Err
       0.1D-04             -
276    198:  3  3  0  2  2  0   S 2   9.4481593   -0.0760   9.4480833   Err
       0.1D-04             -
277    199:  3 K  -3  2 K  -2   S 3   9.4521748   -1.9517   9.4502232   Err
       0.1D-04             -
278    200:  3 K   3  2 K   2   S 4   9.4517559   -1.6823   9.4500736   Err
       0.1D-04             -
279    201:  3 K  -3  2 K  -2   S 5   9.4525702   -2.1994   9.4503708   Err
       0.1D-04             -
280    202:  3  3  1  2  2  1   S 1   9.4673043    0.0469   9.4673513   Err
       0.1D-04             -
281    203:  3  3  1  2  2  1   S 2   9.4671533    0.1979   9.4673513   Err
       0.1D-04             -
282    204:  3 K   3  2 K   2   S 3   9.4629392    2.1566   9.4650958   Err
       0.1D-04             -
283    205:  3 K  -3  2 K  -2   S 4   9.4633431    1.8867   9.4652299   Err
       0.1D-04             -
284    206:  3 K   3  2 K   2   S 5   9.4625288    2.4191   9.4649478   Err
       0.1D-04             -
285    207:  5  2  4  4  1  4   S 1  11.8014557    0.0314  11.8014871   Err
       0.1D-04             -
286    Maximum (obs-calc)/err in line 155   0.0043146
287
288    indep.par: 10 stepw:1.0000 lambda:0.400D-05 cond.no:0.188D+03
289
290    ------------------------------------------------------
291    Iteration :  1
292    Sigma:0.236618D-03  Sigma/OldSigma:0.227821  conv: 1
293
294                    Parameters     Change
295    BJ               0.836004248 { 0.000000087}
296    BK               0.887783352 { 0.000015184}
297    B-               0.079159483 { 0.000001824}
298    DJ               0.021434E-6 {-0.030926E-6}
299    DJK              0.648638E-6 { 0.356280E-6}
300    DK              -0.496838E-6 {-0.381766E-6}
301    dj               0.069738E-6 { 0.047815E-6}
302    dk              -1.653248E-6 {-1.071891E-6}
303   \F12              0.810821390 {     derived}
304   \F              161.464802619 {     derived}  161.299619232 {     derived}
```

```
 V1n          13458.009760 { 1062.940540}   19435.311014 { 1813.567084}
\rho           0.007126217 {     derived}    0.006458880 {     derived}
\beta          0.600686815 {     derived}    0.913398805 {     derived}
\gamma         1.215425954 {     derived}    0.426129541 {     derived}
 DJ     (1)    0.021434E-6  -0.030926E-6 144.289% Max. Change

 indep.par: 10 stepw:1.0000 lambda:0.160D-05 cond.no:0.187D+03

 ----------------------------------------------------
 Iteration :  2
 Sigma:0.227112D-04  Sigma/OldSigma:0.095982  conv: 1

                Parameters     Change
 BJ             0.836004255 { 0.000000007}
 BK             0.887784791 { 0.000001439}
 B-             0.079158100 {-0.000001384}
 DJ             0.060980E-6 { 0.039547E-6}
 DJK            0.305139E-6 {-0.343499E-6}
 DK            -0.144750E-6 { 0.352089E-6}
 dj             0.018891E-6 {-0.050847E-6}
 dk            -0.593780E-6 { 1.059469E-6}
\F12            0.810821914 {     derived}
\F            161.464803881 {     derived}  161.299618790 {     derived}
 V1n          13850.214355 {  392.204594}   20634.275719 { 1198.964705}
\rho           0.007126224 {     derived}    0.006458879 {     derived}
\beta          0.600687092 {     derived}    0.913397948 {     derived}
\gamma         1.215427044 {     derived}    0.426130799 {     derived}
 dj     (1)    0.018891E-6  -0.050847E-6 269.162% Max. Change

 indep.par: 10 stepw:1.0000 lambda:0.640D-06 cond.no:0.187D+03

 ----------------------------------------------------
 Iteration :  3
 Sigma:0.951837D-05  Sigma/OldSigma:0.419105  conv: 1

                Parameters     Change
 BJ             0.836004267 { 0.000000013}
 BK             0.887784932 { 0.000000141}
 B-             0.079158003 {-0.000000096}
 DJ             0.064374E-6 { 0.003394E-6}
 DJK            0.281351E-6 {-0.023788E-6}
 DK            -0.114670E-6 { 0.030079E-6}
 dj             0.015396E-6 {-0.003495E-6}
 dk            -0.520853E-6 { 0.072926E-6}
\F12            0.810821968 {     derived}
\F            161.464803994 {     derived}  161.299618777 {     derived}
 V1n          13888.272533 {   38.058178}   21000.425154 {  366.149435}
\rho           0.007126225 {     derived}    0.006458879 {     derived}
\beta          0.600687105 {     derived}    0.913397880 {     derived}
\gamma         1.215427121 {     derived}    0.426130888 {     derived}
 DK     (1)   -0.114670E-6   0.030079E-6  26.231% Max. Change

 indep.par: 10 stepw:1.0000 lambda:0.256D-06 cond.no:0.187D+03

 ----------------------------------------------------
 Iteration :  4
 Sigma:0.950264D-05  Sigma/OldSigma:0.998347  conv: 1

                Parameters     Change
 BJ             0.836004268 { 0.000000001}
 BK             0.887784934 { 0.000000002}
 B-             0.079158002 {-0.000000001}
 DJ             0.064434E-6 { 0.000059E-6}
 DJK            0.281027E-6 {-0.000324E-6}
 DK            -0.114165E-6 { 0.000505E-6}
 dj             0.015350E-6 {-0.000046E-6}
 dk            -0.519929E-6 { 0.000924E-6}
\F12            0.810821969 {     derived}
\F            161.464803996 {     derived}  161.299618777 {     derived}
 V1n          13888.727891 {    0.455358}   21027.054420 {   26.629266}
\rho           0.007126225 {     derived}    0.006458879 {     derived}
\beta          0.600687105 {     derived}    0.913397879 {     derived}
\gamma         1.215427122 {     derived}    0.426130889 {     derived}
```

```
 DK     (1)   -0.114165E-6   0.000505E-6   0.442% Max. Change

 indep.par: 10 stepw:1.0000 lambda:0.102D-06 cond.no:0.187D+03

 ----------------------------------------------------
 Iteration :  5
 Sigma:0.950264D-05  Sigma/OldSigma:1.000000  conv: 1

                Parameters     Change
 BJ              0.836004268 { 0.000000000}
 BK              0.887784934 {-0.000000000}
 B-              0.079158002 {-0.000000000}
 DJ              0.064434E-6 { 0.000000E-6}
 DJK             0.281027E-6 {-0.000000E-6}
 DK             -0.114164E-6 { 0.000001E-6}
 dj              0.015350E-6 { 0.000000E-6}
 dk             -0.519930E-6 {-0.000001E-6}
\F12             0.810821969 {     derived}
\F             161.464803996 {     derived}  161.299618777 {     derived}
 Vln           13888.726293 {   -0.001598}   21027.055937 {    0.001517}
\rho             0.007126225 {     derived}    0.006458879 {     derived}
\beta            0.600687105 {     derived}    0.913397879 {     derived}
\gamma           1.215427122 {     derived}    0.426130889 {     derived}
 DK     (1)   -0.114164E-6   0.000001E-6   0.001% Max. Change

 indep.par: 10 stepw:1.0000 lambda:0.410D-07 cond.no:0.187D+03

 ----------------------------------------------------
 Iteration :  6
 Sigma:0.950264D-05  Sigma/OldSigma:1.000000  conv: 1

                Parameters     Change
 BJ              0.836004268 { 0.000000000}
 BK              0.887784934 { 0.000000000}
 B-              0.079158002 {-0.000000000}
 DJ              0.064434E-6 { 0.000000E-6}
 DJK             0.281027E-6 {-0.000000E-6}
 DK             -0.114162E-6 { 0.000002E-6}
 dj              0.015350E-6 {-0.000000E-6}
 dk             -0.519931E-6 {-0.000001E-6}
\F12             0.810821969 {     derived}
\F             161.464803996 {     derived}  161.299618777 {     derived}
 Vln           13888.726647 {    0.000353}   21026.958725 {   -0.097212}
\rho             0.007126225 {     derived}    0.006458879 {     derived}
\beta            0.600687105 {     derived}    0.913397879 {     derived}
\gamma           1.215427122 {     derived}    0.426130889 {     derived}
 DK     (1)   -0.114162E-6   0.000002E-6   0.002% Max. Change

 indep.par: 10 stepw:1.0000 lambda:0.164D-07 cond.no:0.187D+03

 ----------------------------------------------------
 Iteration :  7
 Sigma:0.950264D-05  Sigma/OldSigma:1.000000  conv:-1
 DK     (1)   -0.114164E-6  -0.000002E-6   0.002% Max. Change

 indep.par: 10 stepw:1.0000 lambda:0.164D-06 cond.no:0.187D+03

 ----------------------------------------------------
 Iteration :  8
 Sigma:0.950264D-05  Sigma/OldSigma:1.000000  conv:-2
 DK     (1)   -0.114164E-6  -0.000002E-6   0.002% Max. Change

 indep.par: 10 stepw:1.0000 lambda:0.164D-05 cond.no:0.187D+03

 ----------------------------------------------------
 Iteration :  9
 Switching to better derivatives (takes more time)
 Sigma:0.950264D-05  Sigma/OldSigma:1.000000  conv:-3
 DK     (1)   -0.114164E-6  -0.000002E-6   0.002% Max. Change

 indep.par: 10 stepw:1.0000 lambda:0.164D-04 cond.no:0.186D+03

 ----------------------------------------------------
```

```
 Iteration : 10
 Sigma:0.950264D-05  Sigma/OldSigma:1.000000  conv:-4
 DK     (1)   -0.114164E-6  -0.000002E-6   0.002% Max. Change

 indep.par: 10 stepw:1.0000 lambda:0.164D-03 cond.no:0.185D+03

 ----------------------------------------------------
 Iteration : 11
 Sigma:0.950264D-05  Sigma/OldSigma:1.000000  conv:-5
 DK     (1)   -0.114164E-6  -0.000002E-6   0.002% Max. Change

 indep.par: 10 stepw:1.0000 lambda:0.164D-02 cond.no:0.170D+03

 ----------------------------------------------------
 Iteration : 12
 Sigma:0.950264D-05  Sigma/OldSigma:1.000000  conv:-6
 DK     (1)   -0.114164E-6  -0.000002E-6   0.002% Max. Change

 indep.par: 10 stepw:1.0000 lambda:0.164D-01 cond.no:0.950D+02

 ----------------------------------------------------
 Iteration : 13
 Sigma:0.950264D-05  Sigma/OldSigma:1.000000  conv:-7
 DK     (1)   -0.114164E-6  -0.000001E-6   0.001% Max. Change

 indep.par: 10 stepw:1.0000 lambda:0.164D+00 cond.no:0.183D+02

 ----------------------------------------------------
 Iteration : 14
 Sigma:0.950264D-05  Sigma/OldSigma:1.000000  conv:-8
 DK     (1)   -0.114163E-6  -0.000001E-6   0.001% Max. Change

 indep.par: 10 stepw:1.0000 lambda:0.164D+01 cond.no:0.289D+01

 ----------------------------------------------------
 Iteration : 15
 Sigma:0.950264D-05  Sigma/OldSigma:1.000000  conv:-9
 V1n_2  (1)   21026.995780      0.037055   0.000% Max. Change

 indep.par: 10 stepw:1.0000 lambda:0.164D+02 cond.no:0.119D+01

 ----------------------------------------------------
 Iteration : 16
 Sigma:0.950264D-05  Sigma/OldSigma:1.000000  conv:**
 V1n_2  (1)   21026.964346      0.005621   0.000% Max. Change

 indep.par: 10 stepw:1.0000 lambda:0.164D+03 cond.no:0.102D+01

 ----------------------------------------------------
 Iteration : 17
 Sigma:0.950264D-05  Sigma/OldSigma:1.000000  conv: 1

                Parameters     Change
 BJ              0.836004268 {-0.000000000}
 BK              0.887784934 {-0.000000000}
 B-              0.079158002 { 0.000000000}
 DJ              0.064434E-6 { 0.000000E-6}
 DJK             0.281027E-6 { 0.000000E-6}
 DK             -0.114162E-6 { 0.000000E-6}
 dj              0.015350E-6 {-0.000000E-6}
 dk             -0.519931E-6 {-0.000000E-6}
\F12             0.810821969 {     derived}
\F             161.464803996 {     derived}  161.299618777 {     derived}
 V1n           13888.726644 {   -0.000002}   21026.959318 {    0.000593}
\rho             0.007126225 {     derived}    0.006458879 {     derived}
\beta            0.600687105 {     derived}    0.913397879 {     derived}
\gamma           1.215427122 {     derived}    0.426130889 {     derived}
 V1n_2  (1)   21026.959318      0.000593   0.000% Max. Change

 indep.par: 10 stepw:1.0000 lambda:0.655D+02 cond.no:0.105D+01

 ----------------------------------------------------
 Iteration : 18
```

```
 Sigma:0.950264D-05  Sigma/OldSigma:1.000000  conv:-1
 V1n_2  (1)   21026.960057      0.000739   0.000% Max. Change

 indep.par: 10 stepw:1.0000 lambda:0.655D+03 cond.no:0.100D+01

 ---------------------------------------------------------
 Iteration : 19
 Sigma:0.950264D-05  Sigma/OldSigma:1.000000  conv:-2
 V1n_2  (1)   21026.959393      0.000075   0.000% Max. Change

 indep.par: 10 stepw:1.0000 lambda:0.655D+04 cond.no:0.100D+01

 ---------------------------------------------------------
 Iteration : 20
 Sigma:0.950264D-05  Sigma/OldSigma:1.000000  conv:-3
 V1n_2  (1)   21026.959325      0.000008   0.000% Max. Change

 indep.par: 10 stepw:1.0000 lambda:0.655D+04 cond.no:0.187D+03

 Recalculation of the spectrum
 ###########################################################
  End at Cycle  20

      J K- K+  J K- K+    Sym   calc/GHz  diff/MHz     obs/GHz
   1:  9  3  7  9  2  7   S 1   2.1928145    0.0239   2.1928384   Err
    0.1D-04             -
   2:  9  3  7  9  2  7   S 2   2.1928212    0.0173   2.1928384   Err
    0.1D-04             -
   3:  9 K   3  9 K   0   S 3   2.1927090   -0.0072   2.1927017   Err
    0.1D-04             -
   4:  9 K  -3  9 K   0   S 4   2.1927154   -0.0137   2.1927017   Err
    0.1D-04             -
   5:  9 K   3  9 K   0   S 5   2.1927158   -0.0140   2.1927017   Err
    0.1D-04             -
   6:  3  2  2  3  1  2   S 1   2.2025750   -0.0017   2.2025733   Err
    0.1D-04             -
   7:  3  2  2  3  1  2   S 2   2.2025760   -0.0027   2.2025733   Err
    0.1D-04             -
   8:  3 K   2  3 K  -1   S 3   2.2024952   -0.0000   2.2024952   Err
    0.1D-04             -
   9:  3 K  -2  3 K   1   S 4   2.2025008   -0.0056   2.2024952   Err
    0.1D-04             -
  10:  3 K   2  3 K  -1   S 5   2.2024915    0.0037   2.2024952   Err
    0.1D-04             -
  11:  4  2  2  4  1  3   S 1   2.2114022   -0.0118   2.2113904   Err
    0.1D-04             -
  12:  5  2  3  5  1  4   S 1   2.2205676   -0.0046   2.2205630   Err
    0.1D-04             -
  13:  3  2  1  3  1  2   S 1   2.3054637    0.0013   2.3054650   Err
    0.1D-04             -
  14:  2  0  2  1  1  0   S 1   2.3560262   -0.0127   2.3560135   Err
    0.1D-04             -
  15:  6  2  4  6  1  5   S 1   2.3764551   -0.0031   2.3764520   Err
    0.1D-04             -
  16:  2  2  1  2  1  1   S 1   2.4258975   -0.0093   2.4258882   Err
    0.1D-04             -
  17:  5  1  4  5  0  5   S 1   2.5373291   -0.0035   2.5373256   Err
    0.1D-04             -
  18:  1  1  0  0  0  0   S 1   2.6389598   -0.0056   2.6389542   Err
    0.1D-04             -
  19:  2  2  1  2  1  2   S 1   2.9008403   -0.0038   2.9008365   Err
    0.1D-04             -
  20:  2  2  0  2  1  2   S 1   2.9218895   -0.0001   2.9218894   Err
    0.1D-04             -
  21:  2  2  0  2  1  2   S 2   2.9218892    0.0003   2.9218894   Err
    0.1D-04             -
  22:  2 K  -2  2 K   1   S 3   2.9220815    0.0123   2.9220938   Err
    0.1D-04             -
  23:  2 K   2  2 K  -1   S 4   2.9220588   -0.0224   2.9220364   Err
    0.1D-04             -
  24:  2 K  -2  2 K   1   S 5   2.9221035    0.0220   2.9221255   Err
    0.1D-04             -
  25:  3  2  2  3  1  3   S 1   3.1506905    0.0055   3.1506960   Err
```

```
      0.1D-04             -
573   26:  3  2  2  3  1  3   S 2   3.1506888    0.0072   3.1506960   Err
      0.1D-04             -
574   27:  3 K   2  3 K   1   S 3   3.1506302   -0.0014   3.1506288   Err
      0.1D-04             -
575   28:  3 K  -2  3 K  -1   S 4   3.1506334   -0.0046   3.1506288   Err
      0.1D-04             -
576   29:  3 K   2  3 K   1   S 5   3.1506236    0.0052   3.1506288   Err
      0.1D-04             -
577   30:  4  1  4  3  2  2   S 1   3.1834015    0.0261   3.1834276   Err
      0.1D-04             -
578   31:  2  1  2  1  1  1   S 1   3.1857077   -0.0025   3.1857052   Err
      0.1D-04             -
579   32:  7  3  5  7  2  5   S 1   3.2043481    0.0026   3.2043507   Err
      0.1D-04             -
580   33:  7  3  5  7  2  5   S 2   3.2043525   -0.0018   3.2043507   Err
      0.1D-04             -
581   34:  7 K   3  7 K  -2   S 3   3.2042296   -0.0044   3.2042252   Err
      0.1D-04             -
582   35:  7 K  -3  7 K   2   S 4   3.2042370   -0.0118   3.2042252   Err
      0.1D-04             -
583   36:  7 K   3  7 K  -2   S 5   3.2042310   -0.0058   3.2042252   Err
      0.1D-04             -
584   37:  3  2  1  3  1  3   S 1   3.2535792   -0.0088   3.2535704   Err
      0.1D-04             -
585   38:  3  2  1  3  1  3   S 2   3.2535772   -0.0068   3.2535704   Err
      0.1D-04             -
586   39:  3 K  -2  3 K   1   S 3   3.2536095    0.0034   3.2536129   Err
      0.1D-04             -
587   40:  3 K   2  3 K  -1   S 4   3.2536033    0.0096   3.2536129   Err
      0.1D-04             -
588   41:  3 K  -2  3 K   1   S 5   3.2536116    0.0013   3.2536129   Err
      0.1D-04             -
589   42:  2  0  2  1  0  1   S 1   3.3229738   -0.0070   3.3229667   Err
      0.1D-04             -
590   43:  4  2  3  4  1  4   S 1   3.4881765   -0.0188   3.4881577   Err
      0.1D-04             -
591   44:  2  1  1  1  1  0   S 1   3.5023360   -0.0152   3.5023209   Err
      0.1D-04             -
592   45:  6  3  4  6  2  4   S 1   3.6323969    0.0037   3.6324006   Err
      0.1D-04             -
593   46:  6  3  4  6  2  4   S 2   3.6323999    0.0008   3.6324006   Err
      0.1D-04             -
594   47:  6 K   3  6 K  -2   S 3   3.6322472    0.0014   3.6322486   Err
      0.1D-04             -
595   48:  6 K  -3  6 K   2   S 4   3.6322578   -0.0092   3.6322486   Err
      0.1D-04             -
596   49:  6 K   3  6 K  -2   S 5   3.6322425    0.0061   3.6322486   Err
      0.1D-04             -
597   50:  4  2  2  4  1  4   S 1   3.7822855    0.0062   3.7822917   Err
      0.1D-04             -
598   51:  3  0  3  2  1  1   S 1   3.7878813    0.0139   3.7878952   Err
      0.1D-04             -
599   52:  3  0  3  2  1  1   S 2   3.7878806    0.0147   3.7878952   Err
      0.1D-04             -
600   53:  3 K   0  2 K  -1   S 3   3.7878529   -0.0071   3.7878458   Err
      0.1D-04             -
601   54:  3 K   0  2 K   1   S 4   3.7878531   -0.0073   3.7878458   Err
      0.1D-04             -
602   55:  3 K   0  2 K  -1   S 5   3.7878511   -0.0053   3.7878458   Err
      0.1D-04             -
603   56:  5  2  4  4  3  2   S 1   3.8082272   -0.0023   3.8082249   Err
      0.1D-04             -
604   57:  5  3  3  5  2  3   S 1   3.9730617    0.0011   3.9730628   Err
      0.1D-04             -
605   58:  5  3  3  5  2  3   S 2   3.9730629   -0.0002   3.9730628   Err
      0.1D-04             -
606   59:  5 K   3  5 K  -2   S 3   3.9727855   -0.0078   3.9727778   Err
      0.1D-04             -
607   60:  5 K  -3  5 K   2   S 4   3.9728092    0.0263   3.9728356   Err
      0.1D-04             -
608   61:  5 K   3  5 K  -2   S 5   3.9727645   -0.0190   3.9727455   Err
      0.1D-04             -
```

```
609     62:  2  1  2  1  0  1   S 1   3.9943407    0.0018   3.9943426   Err
        0.1D-04              -
610     63:  2  1  2  1  0  1   S 2   3.9943400    0.0026   3.9943426   Err
        0.1D-04              -
611     64:  2 K   1  1 K   0   S 3   3.9943158   -0.0165   3.9942993   Err
        0.1D-04              -
612     65:  2 K  -1  1 K   0   S 4   3.9943151   -0.0157   3.9942993   Err
        0.1D-04              -
613     66:  2 K   1  1 K   0   S 5   3.9943151   -0.0158   3.9942993   Err
        0.1D-04              -
614     67:  4  3  2  4  2  2   S 1   4.2109695   -0.0021   4.2109674   Err
        0.1D-04              -
615     68:  4  3  2  4  2  2   S 2   4.2109680   -0.0005   4.2109674   Err
        0.1D-04              -
616     69:  4 K   3  4 K  -2   S 3   4.2101315   -0.0040   4.2101275   Err
        0.1D-04              -
617     70:  4 K  -3  4 K   2   S 4   4.2102097    0.0014   4.2102111   Err
        0.1D-04              -
618     71:  4 K   3  4 K  -2   S 5   4.2100516   -0.0067   4.2100449   Err
        0.1D-04              -
619     72:  3  0  3  2  1  2   S 1   4.2628242   -0.0025   4.2628217   Err
        0.1D-04              -
620     73:  3  3  1  3  2  1   S 1   4.3484406    0.0206   4.3484612   Err
        0.1D-04              -
621     74:  3  3  1  3  2  1   S 2   4.3484245   -0.0013   4.3484232   Err
        0.1D-04              -
622     75:  3 K   3  3 K  -2   S 3   4.3459369    0.0062   4.3459431   Err
        0.1D-04              -
623     76:  3 K  -3  3 K   2   S 4   4.3461046    0.0060   4.3461107   Err
        0.1D-04              -
624     77:  3 K   3  3 K  -2   S 5   4.3457686   -0.0015   4.3457671   Err
        0.1D-04              -
625     78:  3 K  -3  3 K  -2   S 3   4.3525304   -0.0031   4.3525273   Err
        0.1D-04              -
626     79:  3 K   3  3 K   2   S 4   4.3523719    0.0007   4.3523726   Err
        0.1D-04              -
627     80:  3 K  -3  3 K  -2   S 5   4.3526897   -0.0132   4.3526765   Err
        0.1D-04              -
628     81:  5  1  5  4  2  3   S 1   4.4004794   -0.0052   4.4004742   Err
        0.1D-04              -
629     82:  3  3  0  3  2  2   S 1   4.4530972    0.0124   4.4531097   Err
        0.1D-04              -
630     83:  3  3  0  3  2  2   S 2   4.4531131   -0.0034   4.4531097   Err
        0.1D-04              -
631     84:  3 K  -3  3 K   2   S 3   4.4555097    0.0011   4.4555108   Err
        0.1D-04              -
632     85:  3 K   3  3 K  -2   S 4   4.4553418    0.0030   4.4553448   Err
        0.1D-04              -
633     86:  3 K  -3  3 K   2   S 5   4.4556777   -0.0028   4.4556749   Err
        0.1D-04              -
634     87:  2  1  1  1  0  1   S 1   4.4692836   -0.0037   4.4692799   Err
        0.1D-04              -
635     88:  4  3  2  4  2  3   S 1   4.5050785   -0.0032   4.5050753   Err
        0.1D-04              -
636     89:  4  3  2  4  2  3   S 2   4.5050756   -0.0003   4.5050753   Err
        0.1D-04              -
637     90:  4 K   3  4 K   2   S 3   4.5042843   -0.0021   4.5042822   Err
        0.1D-04              -
638     91:  4 K  -3  4 K  -2   S 4   4.5043581    0.0025   4.5043606   Err
        0.1D-04              -
639     92:  4 K   3  4 K   2   S 5   4.5042060    0.0007   4.5042067   Err
        0.1D-04              -
640     93:  4  3  1  4  2  3   S 1   4.5173295   -0.0015   4.5173279   Err
        0.1D-04              -
641     94:  4  3  1  4  2  3   S 2   4.5173310   -0.0031   4.5173279   Err
        0.1D-04              -
642     95:  4 K  -3  4 K   2   S 3   4.5180727   -0.0014   4.5180713   Err
        0.1D-04              -
643     96:  4 K   3  4 K  -2   S 4   4.5179947   -0.0078   4.5179869   Err
        0.1D-04              -
644     97:  4 K  -3  4 K   2   S 5   4.5181526   -0.0051   4.5181475   Err
        0.1D-04              -
645     98:  5  2  3  5  1  5   S 1   4.5472394    0.0096   4.5472490   Err
```

```
       0.1D-04                -
646     99:  4  1  3  3  2  1   S 1   4.6513962    0.0144   4.6514106   Err
       0.1D-04                -
647    100:  4  1  3  3  2  1   S 2   4.6513922    0.0184   4.6514106   Err
       0.1D-04                -
648    101:  4 K  -1  3 K  -2   S 3   4.6513678   -0.0093   4.6513585   Err
       0.1D-04                -
649    102:  4 K   1  3 K   2   S 4   4.6513685   -0.0100   4.6513585   Err
       0.1D-04                -
650    103:  4 K  -1  3 K  -2   S 5   4.6513592   -0.0007   4.6513585   Err
       0.1D-04                -
651    104:  5  3  2  5  2  4   S 1   4.6554875   -0.0009   4.6554866   Err
       0.1D-04                -
652    105:  5  3  2  5  2  4   S 2   4.6554865    0.0001   4.6554866   Err
       0.1D-04                -
653    106:  5 K  -3  5 K   2   S 3   4.6556661   -0.0007   4.6556654   Err
       0.1D-04                -
654    107:  5 K   3  5 K  -2   S 4   4.6556429   -0.0265   4.6556164   Err
       0.1D-04                -
655    108:  5 K  -3  5 K   2   S 5   4.6556873    0.0141   4.6557014   Err
       0.1D-04                -
656    109:  3  1  3  2  1  2   S 1   4.7661737    0.0119   4.7661856   Err
       0.1D-04                -
657    110:  3  1  3  2  1  2   S 2   4.7661724    0.0132   4.7661856   Err
       0.1D-04                -
658    111:  3 K   1  2 K   1   S 3   4.7661564   -0.0052   4.7661512   Err
       0.1D-04                -
659    112:  3 K  -1  2 K  -1   S 4   4.7661547   -0.0035   4.7661512   Err
       0.1D-04                -
660    113:  3 K   1  2 K   1   S 5   4.7661556   -0.0044   4.7661512   Err
       0.1D-04                -
661    114:  6  3  3  6  2  5   S 1   4.9129964    0.0003   4.9129967   Err
       0.1D-04                -
662    115:  3  0  3  2  0  2   S 1   4.9341912    0.0014   4.9341926   Err
       0.1D-04                -
663    116:  3  0  3  2  0  2   S 2   4.9341896    0.0030   4.9341926   Err
       0.1D-04                -
664    117:  3 K   0  2 K   0   S 3   4.9341660   -0.0093   4.9341567   Err
       0.1D-04                -
665    118:  3 K   0  2 K   0   S 4   4.9341648   -0.0081   4.9341567   Err
       0.1D-04                -
666    119:  3 K   0  2 K   0   S 5   4.9341640   -0.0073   4.9341567   Err
       0.1D-04                -
667    120:  4  0  4  3  1  2   S 1   5.0452254    0.0111   5.0452365   Err
       0.1D-04                -
668    121:  3  1  2  2  1  1   S 1   5.2393463   -0.0045   5.2393418   Err
       0.1D-04                -
669    122:  6  2  5  5  3  3   S 1   5.3309488   -0.0270   5.3309218   Err
       0.1D-04                -
670    123:  7  3  4  7  2  6   S 1   5.3444978   -0.0089   5.3444889   Err
       0.1D-04                -
671    124:  3  1  3  2  0  2   S 1   5.4375406    0.0118   5.4375524   Err
       0.1D-04                -
672    125:  3  1  3  2  0  2   S 2   5.4375398    0.0126   5.4375524   Err
       0.1D-04                -
673    126:  3 K   1  2 K   0   S 3   5.4375122   -0.0005   5.4375116   Err
       0.1D-04                -
674    127:  3 K  -1  2 K   0   S 4   5.4375107    0.0009   5.4375116   Err
       0.1D-04                -
675    128:  3 K   1  2 K   0   S 5   5.4375121   -0.0005   5.4375116   Err
       0.1D-04                -
676    129:  6  1  6  5  2  4   S 1   5.5180990   -0.0005   5.5180985   Err
       0.1D-04                -
677    130:  8  4  4  7  5  2   S 1   5.6601533    0.0089   5.6601621   Err
       0.1D-04                -
678    131:  7  4  4  7  3  4   S 1   5.8574417    0.0068   5.8574485   Err
       0.1D-04                -
679    132:  7  4  4  7  3  4   S 2   5.8574409    0.0075   5.8574485   Err
       0.1D-04                -
680    133:  7 K   4  7 K  -3   S 3   5.8564713    0.0086   5.8564799   Err
       0.1D-04                -
681    134:  2  2  1  1  1  0   S 1   5.9282335   -0.0035   5.9282300   Err
       0.1D-04                -
```

```
682    135:  2  2  0  1  1  0   S 1   5.9492827    0.0000   5.9492827   Err
       0.1D-04                 -
683    136:  2  2  0  1  1  0   S 2   5.9492819    0.0009   5.9492827   Err
       0.1D-04                 -
684    137:  2 K   2  1 K   1   S 4   5.9494262   -0.0147   5.9494115   Err
       0.1D-04                 -
685    138:  2 K  -2  1 K  -1   S 5   5.9494692    0.0098   5.9494790   Err
       0.1D-04                 -
686    139:  4  0  4  3  1  3   S 1   5.9933408   -0.0015   5.9933394   Err
       0.1D-04                 -
687    140:  6  4  3  6  3  3   S 1   6.0305099   -0.0022   6.0305077   Err
       0.1D-04                 -
688    141:  2  2  1  1  1  1   S 1   6.0865480   -0.0022   6.0865458   Err
       0.1D-04                 -
689    142:  2  2  1  1  1  1   S 2   6.0865457    0.0002   6.0865458   Err
       0.1D-04                 -
690    143:  2 K  -2  1 K  -1   S 4   6.0863292    0.0034   6.0863326   Err
       0.1D-04                 -
691    144:  2 K   2  1 K   1   S 5   6.0862862   -0.0212   6.0862650   Err
       0.1D-04                 -
692    145:  2  2  0  1  1  1   S 1   6.1075972   -0.0081   6.1075891   Err
       0.1D-04                 -
693    146:  5  4  2  5  3  2   S 1   6.1242478   -0.0170   6.1242308   Err
       0.1D-04                 -
694    147:  5  4  2  5  3  3   S 2   6.1722162   -0.0040   6.1722122   Err
       0.1D-04                 -
695    148:  4 K  -4  4 K  -3   S 3   6.1724787    0.0054   6.1724841   Err
       0.1D-04                 -
696    149:  4 K   4  4 K   3   S 4   6.1723280   -0.0098   6.1723183   Err
       0.1D-04                 -
697    150:  4 K  -4  4 K  -3   S 5   6.1726257    0.0093   6.1726350   Err
       0.1D-04                 -
698    151:  5  4  1  5  3  3   S 1   6.1733597    0.0128   6.1733726   Err
       0.1D-04                 -
699    152:  5  4  1  5  3  3   S 2   6.1734046   -0.0321   6.1733726   Err
       0.1D-04                 -
700    153:  5 K  -4  5 K   3   S 3   6.1772128   -0.0049   6.1772079   Err
       0.1D-04                 -
701    154:  5 K   4  5 K  -3   S 4   6.1769676    0.0042   6.1769719   Err
       0.1D-04                 -
702    155:  5 K  -4  5 K   3   S 5   6.1774588    0.0075   6.1774663   Err
       0.1D-04                 -
703    156:  6  4  2  6  3  4   S 1   6.1747837    0.0020   6.1747857   Err
       0.1D-04                 -
704    157:  6  4  2  6  3  4   S 2   6.1747928   -0.0070   6.1747857   Err
       0.1D-04                 -
705    158:  6 K  -4  6 K   3   S 3   6.1770835   -0.0012   6.1770824   Err
       0.1D-04                 -
706    159:  6 K   4  6 K  -3   S 4   6.1768887    0.0092   6.1768979   Err
       0.1D-04                 -
707    160:  6 K  -4  6 K   3   S 5   6.1772809   -0.0077   6.1772732   Err
       0.1D-04                 -
708    161:  4 K   4  4 K   3   S 3   6.1777944    0.0086   6.1778030   Err
       0.1D-04                 -
709    162:  4 K  -4  4 K  -3   S 4   6.1779447    0.0017   6.1779464   Err
       0.1D-04                 -
710    163:  4 K   4  4 K   3   S 5   6.1776470    0.0061   6.1776531   Err
       0.1D-04                 -
711    164:  4  4  0  4  3  2   S 1   6.1813865    0.0036   6.1813901   Err
       0.1D-04                 -
712    165:  4  4  0  4  3  2   S 2   6.1815616   -0.0033   6.1815584   Err
       0.1D-04                 -
713    166:  7  4  3  7  3  5   S 1   6.2037122   -0.0027   6.2037094   Err
       0.1D-04                 -
714    167:  7  4  3  7  3  5   S 2   6.2037140   -0.0046   6.2037094   Err
       0.1D-04                 -
715    168:  7 K  -4  7 K   3   S 3   6.2045394    0.0071   6.2045465   Err
       0.1D-04                 -
716    169:  8  4  4  8  3  6   S 1   6.2889790   -0.0041   6.2889749   Err
       0.1D-04                 -
717    170:  3  1  2  2  0  2   S 1   6.3856561   -0.0015   6.3856546   Err
       0.1D-04                 -
718    171:  5  1  4  4  2  2   S 1   6.4330423   -0.0052   6.4330371   Err
```

```
      0.1D-04                -
719   172:  9  4  5  9  3  7   S 1   6.4716689    0.0145   6.4716833   Err
      0.1D-04                -
720   173: 10  4  7 10  3  8   S 1   6.4941417   -0.0061   6.4941356   Err
      0.1D-04                -
721   174:  4  0  4  3  0  3   S 1   6.4966903   -0.0110   6.4966793   Err
      0.1D-04                -
722   175:  4  2  3  3  2  2   S 1   6.6715780    0.0080   6.6715860   Err
      0.1D-04                -
723   176:  4  1  4  3  0  3   S 1   6.8374415    0.0070   6.8374485   Err
      0.1D-04                -
724   177:  4  2  2  3  2  1   S 1   6.8627984   -0.0081   6.8627903   Err
      0.1D-04                -
725   178:  3  2  2  2  1  1   S 1   7.4419213    0.0020   7.4419234   Err
      0.1D-04                -
726   179:  3  2  2  2  1  1   S 2   7.4419196    0.0037   7.4419234   Err
      0.1D-04                -
727   180:  3 K   2  2 K  -1   S 3   7.4418292   -0.0013   7.4418278   Err
      0.1D-04                -
728   181:  3 K  -2  2 K   1   S 4   7.4418322   -0.0044   7.4418278   Err
      0.1D-04                -
729   182:  3 K   2  2 K  -1   S 5   7.4418227    0.0052   7.4418278   Err
      0.1D-04                -
730   183:  3  2  1  2  1  1   S 1   7.5448100   -0.0017   7.5448083   Err
      0.1D-04                -
731   184:  5  0  5  4  1  4   S 1   7.6779987    0.0029   7.6780016   Err
      0.1D-04                -
732   185:  5  0  5  4  1  4   S 2   7.6779958    0.0057   7.6780016   Err
      0.1D-04                -
733   186:  5 K   0  4 K   1   S 3   7.6779058   -0.0063   7.6778995   Err
      0.1D-04                -
734   187:  5 K   0  4 K  -1   S 4   7.6779088   -0.0093   7.6778995   Err
      0.1D-04                -
735   188:  5 K   0  4 K   1   S 5   7.6778972    0.0024   7.6778995   Err
      0.1D-04                -
736   189:  5  1  5  4  1  4   S 1   7.8886559   -0.0057   7.8886502   Err
      0.1D-04                -
737   190:  3  2  2  2  1  2   S 1   7.9168642    0.0014   7.9168655   Err
      0.1D-04                -
738   191:  3  2  2  2  1  2   S 2   7.9168612    0.0044   7.9168655   Err
      0.1D-04                -
739   192:  3 K   2  2 K   1   S 3   7.9167866    0.0072   7.9167938   Err
      0.1D-04                -
740   193:  3 K  -2  2 K  -1   S 4   7.9167880    0.0058   7.9167938   Err
      0.1D-04                -
741   194:  3 K   2  2 K   1   S 5   7.9167792    0.0146   7.9167938   Err
      0.1D-04                -
742   195:  4  1  3  3  0  3   S 1   8.4083248   -0.0034   8.4083214   Err
      0.1D-04                -
743   196:  4  2  2  3  1  2   S 1   9.1682621   -0.0137   9.1682484   Err
      0.1D-04                -
744   197:  3  3  0  2  2  0   S 1   9.4480719    0.0114   9.4480833   Err
      0.1D-04                -
745   198:  3  3  0  2  2  0   S 2   9.4480851   -0.0018   9.4480833   Err
      0.1D-04                -
746   199:  3 K  -3  2 K  -2   S 3   9.4502148    0.0083   9.4502232   Err
      0.1D-04                -
747   200:  3 K   3  2 K   2   S 4   9.4500710    0.0026   9.4500736   Err
      0.1D-04                -
748   201:  3 K  -3  2 K  -2   S 5   9.4503535    0.0173   9.4503708   Err
      0.1D-04                -
749   202:  3  3  1  2  2  1   S 1   9.4673531   -0.0018   9.4673513   Err
      0.1D-04                -
750   203:  3  3  1  2  2  1   S 2   9.4673351    0.0161   9.4673513   Err
      0.1D-04                -
751   204:  3 K   3  2 K   2   S 3   9.4650922    0.0037   9.4650958   Err
      0.1D-04                -
752   205:  3 K  -3  2 K  -2   S 4   9.4652312   -0.0013   9.4652299   Err
      0.1D-04                -
753   206:  3 K   3  2 K   2   S 5   9.4649487   -0.0009   9.4649478   Err
      0.1D-04                -
754   207:  5  2  4  4  1  4   S 1  11.8014822    0.0049  11.8014871   Err
      0.1D-04                -
```

```
 Maximum (obs-calc)/err in line 152   0.0000321

 RMS deviations (MHz), B and V sorted
  B  V   n splittings MHz
  B  V   n abs. freq. MHz
  1  1 207          0.009247          0.009693

            Parameters  and  Errors
 BJ            0.836004268 { 0.000000252}
 BK            0.887784934 { 0.000000408}
 B-            0.079158002 { 0.000000177}
 DJ            0.064434E-6 { 0.005931E-6}
 DJK           0.281027E-6 { 0.021625E-6}
 DK           -0.114162E-6 { 0.025837E-6}
 dj            0.015350E-6 { 0.002444E-6}
 dk           -0.519931E-6 { 0.056386E-6}
\F12           0.810821969 {     derived}
\F           161.464803996 {     derived}  161.299618777 {     derived}
 V1n          13888.726644 {    1.459075}   21026.959318 {   36.646963}
\rho           0.007126225 {     derived}    0.006458879 {     derived}
\beta          0.600687105 {     derived}    0.913397879 {     derived}
\gamma         1.215427122 {     derived}    0.426130889 {     derived}
 F0          160.398000000 {     fixed  }  160.297000000 {     fixed  }
 epsil         1.273000000 {     fixed  }    0.502000000 {     fixed  }
 delta         0.992000000 {     fixed  }    1.195000000 {     fixed  }

 Standard Deviation       0.009503 MHz

------------------------------------ B =  1
 Rotational Constants and Errors (in GHz)
      B_z       1.723789202       0.000000453
      B_x       0.915162270       0.000000317
      B_y       0.756846266       0.000000298
 Ray's kappa   -0.67254
 F0(calc)     160.398000000       0.000000000
 I_alpha        3.150781618       0.000000000
 <(i,x)  <(i,y)  <(i,z)       75.7813       36.8435       56.8374
d<(i,x) d<(i,y) d<(i,z)        0.0000        0.0000        0.0000

 F0(calc)     160.297000000       0.000000000
 I_alpha        3.152766864       0.000000000
 <(i,x)  <(i,y)  <(i,z)       35.3684       63.4101       68.4685
d<(i,x) d<(i,y) d<(i,z)        0.0000        0.0000        0.0000

 V1n 1      5.542037 kj +/- 0.000582 kj         1.323660 kcal +/- 0.000139 kcal
          463.277989 cm +/-   0.0487 cm    s=  38.229801
 V1n_2      8.390415 kj +/- 0.014623 kj         2.003967 kcal +/- 0.003493 kcal
          701.383768 cm +/-   1.2224 cm    s=  57.878343

F(calc)       161.464803996
F(calc)       161.299618777

 Errors of fitted linear combinations
    0.000000252    0.000000408    0.000000177    1.459074862    0.000000006
    0.000000022    0.000000026    0.000000002    0.000000056   36.646963076

 Correlation Matrix of fitted linear combinations
 BJ      1.000
 BK     -0.120  1.000
 B-      0.066 -0.221  1.000
 V1n_1  -0.064 -0.029  0.042  1.000
 DJ      0.756 -0.121  0.078 -0.110  1.000
 DJK    -0.083  0.374 -0.276  0.075 -0.418  1.000
 DK      0.100  0.427 -0.024 -0.188  0.385 -0.573  1.000
 dj     -0.000  0.174  0.194  0.071 -0.341  0.819 -0.531  1.000
 dk      0.014 -0.301  0.351 -0.052  0.362 -0.910  0.445 -0.807  1.000
 V1n_2  -0.014 -0.009  0.016  0.010 -0.027  0.014 -0.045  0.012 -0.002  1.000
 strongest correlation between  9 and  6 (-0.9102)

 Freedom Cofreedom Matrix of linear comb.
 BJ      0.573
 BK      0.996  0.377
 B-      0.996  0.976  0.421
```

```
 V1n_1   0.999  0.991  0.999  0.961
 DJ      0.629  0.992  0.997  0.997  0.516
 DJK     0.998  0.722  0.944  0.993  0.952  0.186
 DK      0.997  0.623  0.992  0.978  0.949  0.681  0.321
 dj      0.999  0.978  0.677  0.999  0.969  0.720  0.914  0.250
 dk      0.992  0.954  0.867  0.997  0.955  0.620  0.914  0.690  0.256
 V1n_2   1.000  1.000  1.000  1.000  1.000  1.000  0.999  1.000  1.000  0.998
 minimum cofreedom between  9 and  6 ( 0.6197)

 Eigenvalues and Eigenvector Matrix of SVD-FIT

0.168199D-01  -0.019 0.101-0.061 0.012-0.112 0.686-0.245 0.463-0.478 0.002
0.800339D-01  -0.012 0.638-0.348-0.039 0.049 0.138 0.614-0.217-0.158-0.010
0.127992D+00  -0.163-0.245-0.661 0.020-0.168 0.130-0.353-0.548-0.085 0.004
0.176188D+00   0.654-0.246-0.215-0.042 0.642 0.000 0.031-0.008-0.225-0.012
0.369122D+00   0.189 0.405 0.171 0.077 0.237 0.429-0.376-0.256 0.563 0.030
0.941030D+00  -0.069-0.086-0.013-0.972 0.019 0.130 0.032 0.020 0.148-0.048
0.998471D+00   0.020 0.007-0.002-0.059-0.015-0.024 0.004-0.001-0.030 0.997
0.166222D+01   0.652 0.133 0.199-0.121-0.623-0.088-0.088-0.242-0.196-0.038
0.248907D+01   0.279-0.100-0.505 0.092-0.296 0.034 0.157 0.488 0.544 0.012
0.313906D+01   0.023-0.513 0.261 0.138-0.109 0.532 0.513-0.276 0.120 0.025
```

```
 Rotational, Centrifugal Distortion, Internal Rotation Calculation (V2.5e)
                       Holger Hartwig 08-Nov-96 (hartwig@phc.uni-kiel.de)

 Please cite: H.Hartwig and H.Dreizler, Z.Naturforsch, 51a (1996) 923.

 Calculation date and time:
 Modified version to include Dc3- and Dc3K

 Type help now for the list of parameters :
sBT-Si Me5Me7

   nzyk       200   print        4   eval         0   dfreq        0
   orger        0   ints         0   maxm         8   woods       33
   ndata     5000   nfold        3   spin         0   ntop         2
   adjf        16   maxvm        0   aprint      10   xprint       4
   ncycl      200   svderr       0   fitscl       0   reduct       0
   rofit    .0000000D+00   eps      .1000000D-11   defer    .1000000D-04
   weigf    .0000000D+00   convg    .1000000D+01   lambda   .1000000D-04
   freq_l   .6000000D+01   freq_h   .4000000D+02   limit    .1000000D+00
   temp     .2730000D+03
 Using Watson A Reduction

 assumed sizeb           1
 \\ set (adj  or  8)
 \\ set (adj  or  1)
 \\ set (adj  or  2)
 \\ adj  1: adjust F according to rho, beta and gamma
 \\ adj  2: adjust F12 according to rho, beta and gamma
 \\ adj  8: adjust rho according to F0 = 1/(2 I_alpha)
 \\ adj 16: adjust beta and gamma  according delta + epsil
 new adj :  27
 BJ            0.836004161
 BK            0.887768168
 B-            0.079157659
 DJ            0.052360E-6
 DJK           0.292358E-6
 DK           -0.115072E-6
 dj            0.021923E-6
 dk           -0.581357E-6
\F12           0.213836417
\F           161.461008061                 160.498267958
 V1n          12395.069220                  15088.842770
\rho           0.007126178                   0.005326176
\beta          0.600691769                   1.613791130
\gamma         1.215427384                   2.512532580
 F0          160.398000000                 159.647000000
 epsil         1.273000000                   2.420000000
 delta         0.992000000                   1.592000000

 fit  0.1D+00 0.1D+01   BJ          1.00
 fit  0.1D+00 0.1D+01   BK          1.00
 fit  0.1D+00 0.1D+01   B-          1.00
 fit  0.1D+00 0.1D+01   V1n_1       1.00
 fit  0.1D+00 0.1D+01   DJ          1.00
 fit  0.1D+00 0.1D+01   DJK         1.00
 fit  0.1D+00 0.1D+01   DK          1.00
 fit  0.1D+00 0.1D+01   dj          1.00
 fit  0.1D+00 0.1D+01   dk          1.00
 fit  0.1D+00 0.1D+01   V1n_2       1.00

  S     0   0
  S     0   1
  S     1   0
  S    -1   1
  S     1   1

  V  0  0

   ndata  207   Data Points 207   Splittings   0
   Effective Data Points  92.0

  \\  Maximal K = J =  10
  \\ B=  1    adj= 27
```

```
 \\ (1) calculate torsional integrals
 \\ (32)use torsional integrals in rigid rotor H_rr
Sigma:0.103003D-02  Sigma/OldSigma:0.000000  conv: 1

      J K- K+  J K- K+    Sym   calc/GHz  diff/MHz     obs/GHz
  1:  9  3  7  9  2  7   S 1   2.1927982    0.0402   2.1928384   Err
  0.1D-04              -
  2:  9  3  7  9  2  7   S 2   2.1928429   -0.0045   2.1928384   Err
  0.1D-04              -
  3:  9 K   3  9 K   0   S 3   2.1926101    0.0916   2.1927017   Err
  0.1D-04              -
  4:  9 K  -3  9 K   0   S 4   2.1926394    0.0624   2.1927017   Err
  0.1D-04              -
  5:  9 K   3  9 K   0   S 5   2.1926704    0.0313   2.1927017   Err
  0.1D-04              -
  6:  3  2  2  3  1  2   S 1   2.2025388    0.0345   2.2025733   Err
  0.1D-04              -
  7:  3  2  2  3  1  2   S 2   2.2025519    0.0214   2.2025733   Err
  0.1D-04              -
  8:  3 K   2  3 K  -1   S 3   2.2023317    0.1635   2.2024952   Err
  0.1D-04              -
  9:  3 K  -2  3 K   1   S 4   2.2023388    0.1564   2.2024952   Err
  0.1D-04              -
 10:  3 K   2  3 K  -1   S 5   2.2023510    0.1442   2.2024952   Err
 0.1D-04               -
 11:  4  2  2  4  1  3   S 1   2.2113676    0.0228   2.2113904   Err
 0.1D-04               -
 12:  5  2  3  5  1  4   S 1   2.2205340    0.0290   2.2205630   Err
 0.1D-04               -
 13:  3  2  1  3  1  2   S 1   2.3054267    0.0383   2.3054650   Err
 0.1D-04               -
 14:  2  0  2  1  1  0   S 1   2.3560535   -0.0400   2.3560135   Err
 0.1D-04               -
 15:  6  2  4  6  1  5   S 1   2.3764190    0.0331   2.3764520   Err
 0.1D-04               -
 16:  2  2  1  2  1  1   S 1   2.4258580    0.0302   2.4258882   Err
 0.1D-04               -
 17:  5  1  4  5  0  5   S 1   2.5372885    0.0370   2.5373256   Err
 0.1D-04               -
 18:  1  1  0  0  0  0   S 1   2.6389509    0.0033   2.6389542   Err
 0.1D-04               -
 19:  2  2  1  2  1  2   S 1   2.9007953    0.0412   2.9008365   Err
 0.1D-04               -
 20:  2  2  0  2  1  2   S 1   2.9218444    0.0450   2.9218894   Err
 0.1D-04               -
 21:  2  2  0  2  1  2   S 2   2.9218495    0.0399   2.9218894   Err
 0.1D-04               -
 22:  2 K  -2  2 K   1   S 3   2.9224861   -0.3923   2.9220938   Err
 0.1D-04               -
 23:  2 K   2  2 K  -1   S 4   2.9225126   -0.3871   2.9221255   Err
 0.1D-04               -
 24:  2 K  -2  2 K   1   S 5   2.9224699   -0.4335   2.9220364   Err
 0.1D-04               -
 25:  3  2  2  3  1  3   S 1   3.1506420    0.0540   3.1506960   Err
 0.1D-04               -
 26:  3  2  2  3  1  3   S 2   3.1506436    0.0524   3.1506960   Err
 0.1D-04               -
 27:  3 K   2  3 K   1   S 3   3.1504638    0.1650   3.1506288   Err
 0.1D-04               -
 28:  3 K  -2  3 K  -1   S 4   3.1504733    0.1555   3.1506288   Err
 0.1D-04               -
 29:  3 K   2  3 K   1   S 5   3.1504575    0.1713   3.1506288   Err
 0.1D-04               -
 30:  4  1  4  3  2  2   S 1   3.1834820   -0.0543   3.1834276   Err
 0.1D-04               -
 31:  2  1  2  1  1  1   S 1   3.1857219   -0.0166   3.1857052   Err
 0.1D-04               -
 32:  7  3  5  7  2  5   S 1   3.2043018    0.0489   3.2043507   Err
 0.1D-04               -
 33:  7  3  5  7  2  5   S 2   3.2043363    0.0144   3.2043507   Err
 0.1D-04               -
 34:  7 K   3  7 K  -2   S 3   3.2040477    0.1775   3.2042252   Err
 0.1D-04               -
```

```
113     35:  7 K  -3  7 K   2   S 4   3.2040740    0.1512   3.2042252   Err
        0.1D-04              -
114     36:  7 K   3  7 K  -2   S 5   3.2040904    0.1348   3.2042252   Err
        0.1D-04              -
115     37:  3  2  1  3  1  3   S 1   3.2535300    0.0404   3.2535704   Err
        0.1D-04              -
116     38:  3  2  1  3  1  3   S 2   3.2535293    0.0411   3.2535704   Err
        0.1D-04              -
117     39:  3 K  -2  3 K   1   S 3   3.2536396   -0.0267   3.2536129   Err
        0.1D-04              -
118     40:  3 K   2  3 K  -1   S 4   3.2536550   -0.0421   3.2536129   Err
        0.1D-04              -
119     41:  3 K  -2  3 K   1   S 5   3.2536226   -0.0098   3.2536129   Err
        0.1D-04              -
120     42:  2  0  2  1  0  1   S 1   3.3229861   -0.0194   3.3229667   Err
        0.1D-04              -
121     43:  4  2  3  4  1  4   S 1   3.4881223    0.0354   3.4881577   Err
        0.1D-04              -
122     44:  2  1  1  1  1  0   S 1   3.5023463   -0.0255   3.5023209   Err
        0.1D-04              -
123     45:  6  3  4  6  2  4   S 1   3.6323401    0.0605   3.6324006   Err
        0.1D-04              -
124     46:  6  3  4  6  2  4   S 2   3.6323687    0.0319   3.6324006   Err
        0.1D-04              -
125     47:  6 K   3  6 K  -2   S 3   3.6319659    0.2828   3.6322486   Err
        0.1D-04              -
126     48:  6 K  -3  6 K   2   S 4   3.6319844    0.2643   3.6322486   Err
        0.1D-04              -
127     49:  6 K   3  6 K  -2   S 5   3.6320047    0.2439   3.6322486   Err
        0.1D-04              -
128     50:  4  2  2  4  1  4   S 1   3.7822285    0.0632   3.7822917   Err
        0.1D-04              -
129     51:  3  0  3  2  1  1   S 1   3.7879181   -0.0229   3.7878952   Err
        0.1D-04              -
130     52:  3  0  3  2  1  1   S 2   3.7879007   -0.0055   3.7878952   Err
        0.1D-04              -
131     53:  3 K   0  2 K  -1   S 3   3.7878531   -0.0073   3.7878458   Err
        0.1D-04              -
132     54:  3 K   0  2 K   1   S 4   3.7878451    0.0007   3.7878458   Err
        0.1D-04              -
133     55:  3 K   0  2 K  -1   S 5   3.7878264    0.0194   3.7878458   Err
        0.1D-04              -
134     56:  5  2  4  4  3  2   S 1   3.8083329   -0.1080   3.8082249   Err
        0.1D-04              -
135     57:  5  3  3  5  2  3   S 1   3.9729977    0.0651   3.9730628   Err
        0.1D-04              -
136     58:  5  3  3  5  2  3   S 2   3.9730208    0.0419   3.9730628   Err
        0.1D-04              -
137     59:  5 K   3  5 K  -2   S 3   3.9721969    0.5809   3.9727778   Err
        0.1D-04              -
138     60:  5 K  -3  5 K   2   S 4   3.9721998    0.5457   3.9727455   Err
        0.1D-04              -
139     61:  5 K   3  5 K  -2   S 5   3.9722403    0.5953   3.9728356   Err
        0.1D-04              -
140     62:  2  1  2  1  0  1   S 1   3.9943418    0.0008   3.9943426   Err
        0.1D-04              -
141     63:  2  1  2  1  0  1   S 2   3.9943367    0.0058   3.9943426   Err
        0.1D-04              -
142     64:  2 K   1  1 K   0   S 3   3.9942972    0.0021   3.9942993   Err
        0.1D-04              -
143     65:  2 K  -1  1 K   0   S 4   3.9942874    0.0119   3.9942993   Err
        0.1D-04              -
144     66:  2 K   1  1 K   0   S 5   3.9942971    0.0023   3.9942993   Err
        0.1D-04              -
145     67:  4  3  2  4  2  2   S 1   4.2109014    0.0660   4.2109674   Err
        0.1D-04              -
146     68:  4  3  2  4  2  2   S 2   4.2109199    0.0476   4.2109674   Err
        0.1D-04              -
147     69:  4 K   3  4 K  -2   S 3   4.2084960    1.6316   4.2101275   Err
        0.1D-04              -
148     70:  4 K  -3  4 K   2   S 4   4.2084609    1.5840   4.2100449   Err
        0.1D-04              -
149     71:  4 K   3  4 K  -2   S 5   4.2085683    1.6427   4.2102111   Err
```

```
      0.1D-04             -
150   72:  3  0  3  2  1  2   S 1   4.2628553   -0.0336   4.2628217   Err
      0.1D-04             -
151   73:  3  3  1  3  2  1   S 1   4.3483706    0.0906   4.3484612   Err
      0.1D-04             -
152   74:  3  3  1  3  2  1   S 2   4.3483835    0.0397   4.3484232   Err
      0.1D-04             -
153   75:  3 K   3  3 K  -2   S 3   4.3432111    2.7320   4.3459431   Err
      0.1D-04             -
154   76:  3 K  -3  3 K   2   S 4   4.3431487    2.6184   4.3457671   Err
      0.1D-04             -
155   77:  3 K   3  3 K  -2   S 5   4.3433056    2.8051   4.3461107   Err
      0.1D-04             -
156   78:  3 K  -3  3 K  -2   S 3   4.3548448   -2.3175   4.3525273   Err
      0.1D-04             -
157   79:  3 K   3  3 K   2   S 4   4.3549311   -2.2546   4.3526765   Err
      0.1D-04             -
158   80:  3 K  -3  3 K  -2   S 5   4.3547906   -2.4181   4.3523726   Err
      0.1D-04             -
159   81:  5  1  5  4  2  3   S 1   4.4005773   -0.1030   4.4004742   Err
      0.1D-04             -
160   82:  3  3  0  3  2  2   S 1   4.4530266    0.0831   4.4531097   Err
      0.1D-04             -
161   83:  3  3  0  3  2  2   S 2   4.4530434    0.0663   4.4531097   Err
      0.1D-04             -
162   84:  3 K  -3  3 K   2   S 3   4.4580206   -2.5098   4.4555108   Err
      0.1D-04             -
163   85:  3 K   3  3 K  -2   S 4   4.4581128   -2.4379   4.4556749   Err
      0.1D-04             -
164   86:  3 K  -3  3 K   2   S 5   4.4579558   -2.6111   4.4553448   Err
      0.1D-04             -
165   87:  2  1  1  1  0  1   S 1   4.4692790    0.0009   4.4692799   Err
      0.1D-04             -
166   88:  4  3  2  4  2  3   S 1   4.5050076    0.0677   4.5050753   Err
      0.1D-04             -
167   89:  4  3  2  4  2  3   S 2   4.5050195    0.0558   4.5050753   Err
      0.1D-04             -
168   90:  4 K   3  4 K   2   S 3   4.5027216    1.5606   4.5042822   Err
      0.1D-04             -
169   91:  4 K  -3  4 K  -2   S 4   4.5026853    1.5214   4.5042067   Err
      0.1D-04             -
170   92:  4 K   3  4 K   2   S 5   4.5027821    1.5785   4.5043606   Err
      0.1D-04             -
171   93:  4  3  1  4  2  3   S 1   4.5172586    0.0693   4.5173279   Err
      0.1D-04             -
172   94:  4  3  1  4  2  3   S 2   4.5172709    0.0570   4.5173279   Err
      0.1D-04             -
173   95:  4 K  -3  4 K   2   S 3   4.5194917   -1.4204   4.5180713   Err
      0.1D-04             -
174   96:  4 K   3  4 K  -2   S 4   4.5195577   -1.4103   4.5181475   Err
      0.1D-04             -
175   97:  4 K  -3  4 K   2   S 5   4.5194498   -1.4629   4.5179869   Err
      0.1D-04             -
176   98:  5  2  3  5  1  5   S 1   4.5471684    0.0806   4.5472490   Err
      0.1D-04             -
177   99:  4  1  3  3  2  1   S 1   4.6514549   -0.0443   4.6514106   Err
      0.1D-04             -
178  100:  4  1  3  3  2  1   S 2   4.6514188   -0.0082   4.6514106   Err
     0.1D-04              -
179  101:  4 K  -1  3 K  -2   S 3   4.6513404    0.0181   4.6513585   Err
     0.1D-04              -
180  102:  4 K   1  3 K   2   S 4   4.6513027    0.0558   4.6513585   Err
     0.1D-04              -
181  103:  4 K  -1  3 K  -2   S 5   4.6513060    0.0525   4.6513585   Err
     0.1D-04              -
182  104:  5  3  2  5  2  4   S 1   4.6554153    0.0713   4.6554866   Err
     0.1D-04              -
183  105:  5  3  2  5  2  4   S 2   4.6554234    0.0632   4.6554866   Err
     0.1D-04              -
184  106:  5 K  -3  5 K   2   S 3   4.6560375   -0.3720   4.6556654   Err
     0.1D-04              -
185  107:  5 K   3  5 K  -2   S 4   4.6560674   -0.3661   4.6557014   Err
     0.1D-04              -
```

```
186    108:  5 K  -3  5 K   2   S 5   4.6560237   -0.4072   4.6556164   Err
       0.1D-04                -
187    109:  3  1  3  2  1  2   S 1   4.7661960   -0.0104   4.7661856   Err
       0.1D-04                -
188    110:  3  1  3  2  1  2   S 2   4.7661830    0.0026   4.7661856   Err
       0.1D-04                -
189    111:  3 K   1  2 K   1   S 3   4.7661728   -0.0216   4.7661512   Err
       0.1D-04                -
190    112:  3 K  -1  2 K  -1   S 4   4.7661519   -0.0007   4.7661512   Err
       0.1D-04                -
191    113:  3 K   1  2 K   1   S 5   4.7661678   -0.0166   4.7661512   Err
       0.1D-04                -
192    114:  6  3  3  6  2  5   S 1   4.9129202    0.0765   4.9129967   Err
       0.1D-04                -
193    115:  3  0  3  2  0  2   S 1   4.9342109   -0.0183   4.9341926   Err
       0.1D-04                -
194    116:  3  0  3  2  0  2   S 2   4.9341947   -0.0021   4.9341926   Err
       0.1D-04                -
195    117:  3 K   0  2 K   0   S 3   4.9341589   -0.0022   4.9341567   Err
       0.1D-04                -
196    118:  3 K   0  2 K   0   S 4   4.9341503    0.0064   4.9341567   Err
       0.1D-04                -
197    119:  3 K   0  2 K   0   S 5   4.9341350    0.0217   4.9341567   Err
       0.1D-04                -
198    120:  4  0  4  3  1  2   S 1   5.0452753   -0.0388   5.0452365   Err
       0.1D-04                -
199    121:  3  1  2  2  1  1   S 1   5.2393620   -0.0202   5.2393418   Err
       0.1D-04                -
200    122:  6  2  5  5  3  3   S 1   5.3310658   -0.1441   5.3309218   Err
       0.1D-04                -
201    123:  7  3  4  7  2  6   S 1   5.3444118    0.0771   5.3444889   Err
       0.1D-04                -
202    124:  3  1  3  2  0  2   S 1   5.4375516    0.0008   5.4375524   Err
       0.1D-04                -
203    125:  3  1  3  2  0  2   S 2   5.4375451    0.0074   5.4375524   Err
       0.1D-04                -
204    126:  3 K   1  2 K   0   S 3   5.4375112    0.0004   5.4375116   Err
       0.1D-04                -
205    127:  3 K  -1  2 K   0   S 4   5.4374894    0.0222   5.4375116   Err
       0.1D-04                -
206    128:  3 K   1  2 K   0   S 5   5.4375199   -0.0083   5.4375116   Err
       0.1D-04                -
207    129:  6  1  6  5  2  4   S 1   5.5182201   -0.1216   5.5180985   Err
       0.1D-04                -
208    130:  8  4  4  7  5  2   S 1   5.6603414   -0.1793   5.6601621   Err
       0.1D-04                -
209    131:  7  4  4  7  3  4   S 1   5.8573501    0.0984   5.8574485   Err
       0.1D-04                -
210    132:  7  4  4  7  3  4   S 2   5.8573795    0.0689   5.8574485   Err
       0.1D-04                -
211    133:  7 K   4  7 K  -3   S 3   5.8545168    1.9631   5.8564799   Err
       0.1D-04                -
212    134:  2  2  1  1  1  0   S 1   5.9282044    0.0256   5.9282300   Err
       0.1D-04                -
213    135:  2  2  0  1  1  0   S 1   5.9492535    0.0292   5.9492827   Err
       0.1D-04                -
214    136:  2  2  0  1  1  0   S 2   5.9492510    0.0317   5.9492827   Err
       0.1D-04                -
215    137:  2 K   2  1 K   1   S 4   5.9498507   -0.3717   5.9494790   Err
       0.1D-04                -
216    138:  2 K  -2  1 K  -1   S 5   5.9498186   -0.4071   5.9494115   Err
       0.1D-04                -
217    139:  4  0  4  3  1  3   S 1   5.9933786   -0.0392   5.9933394   Err
       0.1D-04                -
218    140:  6  4  3  6  3  3   S 1   6.0304138    0.0940   6.0305077   Err
       0.1D-04                -
219    141:  2  2  1  1  1  1   S 1   6.0865172    0.0287   6.0865458   Err
       0.1D-04                -
220    142:  2  2  1  1  1  1   S 2   6.0865133    0.0325   6.0865458   Err
       0.1D-04                -
221    143:  2 K  -2  1 K  -1   S 4   6.0857831    0.4818   6.0862650   Err
       0.1D-04                -
222    144:  2 K   2  1 K   1   S 5   6.0858144    0.5182   6.0863326   Err
```

```
      0.1D-04                -
223   145:  2  2  0  1  1  1   S 1   6.1075663    0.0228   6.1075891   Err
      0.1D-04                -
224   146:  5  4  2  5  3  2   S 1   6.1241495    0.0813   6.1242308   Err
      0.1D-04                -
225   147:  5  4  2  5  3  3   S 2   6.1721734    0.0388   6.1722122   Err
      0.1D-04                -
226   148:  4 K  -4  4 K  -3   S 3   6.1742693   -1.7852   6.1724841   Err
      0.1D-04                -
227   149:  4 K   4  4 K   3   S 4   6.1743390   -1.7040   6.1726350   Err
      0.1D-04                -
228   150:  4 K  -4  4 K  -3   S 5   6.1742407   -1.9224   6.1723183   Err
      0.1D-04                -
229   151:  5  4  1  5  3  3   S 1   6.1732613    0.1112   6.1733726   Err
      0.1D-04                -
230   152:  5  4  1  5  3  3   S 2   6.1732907    0.0818   6.1733726   Err
      0.1D-04                -
231   153:  5 K  -4  5 K   3   S 3   6.1809293   -3.7214   6.1772079   Err
      0.1D-04                -
232   154:  5 K   4  5 K  -3   S 4   6.1810669   -3.6006   6.1774663   Err
      0.1D-04                -
233   155:  5 K  -4  5 K   3   S 5   6.1808327   -3.8608   6.1769719   Err
      0.1D-04                -
234   156:  6  4  2  6  3  4   S 1   6.1746863    0.0994   6.1747857   Err
      0.1D-04                -
235   157:  6  4  2  6  3  4   S 2   6.1747078    0.0779   6.1747857   Err
      0.1D-04                -
236   158:  6 K  -4  6 K   3   S 3   6.1801707   -3.0883   6.1770824   Err
      0.1D-04                -
237   159:  6 K   4  6 K  -3   S 4   6.1802908   -3.0176   6.1772732   Err
      0.1D-04                -
238   160:  6 K  -4  6 K   3   S 5   6.1800901   -3.1923   6.1768979   Err
      0.1D-04                -
239   161:  4 K   4  4 K   3   S 3   6.1757031    2.0999   6.1778030   Err
      0.1D-04                -
240   162:  4 K  -4  4 K  -3   S 4   6.1756746    1.9785   6.1776531   Err
      0.1D-04                -
241   163:  4 K   4  4 K   3   S 5   6.1757728    2.1735   6.1779464   Err
      0.1D-04                -
242   164:  4  4  0  4  3  2   S 1   6.1812873    0.1029   6.1813901   Err
      0.1D-04                -
243   165:  4  4  0  4  3  2   S 2   6.1813642    0.1941   6.1815584   Err
      0.1D-04                -
244   166:  7  4  3  7  3  5   S 1   6.2036156    0.0939   6.2037094   Err
      0.1D-04                -
245   167:  7  4  3  7  3  5   S 2   6.2036337    0.0757   6.2037094   Err
      0.1D-04                -
246   168:  7 K  -4  7 K   3   S 3   6.2061889   -1.6424   6.2045465   Err
      0.1D-04                -
247   169:  8  4  4  8  3  6   S 1   6.2888817    0.0932   6.2889749   Err
      0.1D-04                -
248   170:  3  1  2  2  0  2   S 1   6.3856549   -0.0003   6.3856546   Err
      0.1D-04                -
249   171:  5  1  4  4  2  2   S 1   6.4331055   -0.0684   6.4330371   Err
      0.1D-04                -
250   172:  9  4  5  9  3  7   S 1   6.4715665    0.1169   6.4716833   Err
      0.1D-04                -
251   173: 10  4  7 10  3  8   S 1   6.4940357    0.0998   6.4941356   Err
      0.1D-04                -
252   174:  4  0  4  3  0  3   S 1   6.4967193   -0.0400   6.4966793   Err
      0.1D-04                -
253   175:  4  2  3  3  2  2   S 1   6.6716043   -0.0183   6.6715860   Err
      0.1D-04                -
254   176:  4  1  4  3  0  3   S 1   6.8374647   -0.0161   6.8374485   Err
      0.1D-04                -
255   177:  4  2  2  3  2  1   S 1   6.8628225   -0.0323   6.8627903   Err
      0.1D-04                -
256   178:  3  2  2  2  1  1   S 1   7.4419008    0.0226   7.4419234   Err
      0.1D-04                -
257   179:  3  2  2  2  1  1   S 2   7.4418947    0.0286   7.4419234   Err
      0.1D-04                -
258   180:  3 K   2  2 K  -1   S 3   7.4416692    0.1587   7.4418278   Err
      0.1D-04                -
```

```
259    181:  3 K  -2  2 K   1   S 4   7.4416575    0.1703   7.4418278   Err
       0.1D-04             -
260    182:  3 K   2  2 K  -1   S 5   7.4416687    0.1591   7.4418278   Err
       0.1D-04             -
261    183:  3  2  1  2  1  1   S 1   7.5447888    0.0196   7.5448083   Err
       0.1D-04             -
262    184:  5  0  5  4  1  4   S 1   7.6780455   -0.0439   7.6780016   Err
       0.1D-04             -
263    185:  5  0  5  4  1  4   S 2   7.6780014    0.0002   7.6780016   Err
       0.1D-04             -
264    186:  5 K   0  4 K   1   S 3   7.6777823    0.1173   7.6778995   Err
       0.1D-04             -
265    187:  5 K   0  4 K  -1   S 4   7.6778567    0.0428   7.6778995   Err
       0.1D-04             -
266    188:  5 K   0  4 K   1   S 5   7.6776197    0.2798   7.6778995   Err
       0.1D-04             -
267    189:  5  1  5  4  1  4   S 1   7.8886996   -0.0493   7.8886502   Err
       0.1D-04             -
268    190:  3  2  2  2  1  2   S 1   7.9168380    0.0275   7.9168655   Err
       0.1D-04             -
269    191:  3  2  2  2  1  2   S 2   7.9168267    0.0389   7.9168655   Err
       0.1D-04             -
270    192:  3 K   2  2 K   1   S 3   7.9166366    0.1573   7.9167938   Err
       0.1D-04             -
271    193:  3 K  -2  2 K  -1   S 4   7.9166252    0.1686   7.9167938   Err
       0.1D-04             -
272    194:  3 K   2  2 K   1   S 5   7.9166252    0.1686   7.9167938   Err
       0.1D-04             -
273    195:  4  1  3  3  0  3   S 1   8.4083255   -0.0041   8.4083214   Err
       0.1D-04             -
274    196:  4  2  2  3  1  2   S 1   9.1682492   -0.0008   9.1682484   Err
       0.1D-04             -
275    197:  3  3  0  2  2  0   S 1   9.4480202    0.0631   9.4480833   Err
       0.1D-04             -
276    198:  3  3  0  2  2  0   S 2   9.4480206    0.0628   9.4480833   Err
       0.1D-04             -
277    199:  3 K  -3  2 K  -2   S 3   9.4521711   -1.9479   9.4502232   Err
       0.1D-04             -
278    200:  3 K   3  2 K   2   S 4   9.4522254   -1.8546   9.4503708   Err
       0.1D-04             -
279    201:  3 K  -3  2 K  -2   S 5   9.4521112   -2.0376   9.4500736   Err
       0.1D-04             -
280    202:  3  3  1  2  2  1   S 1   9.4673013    0.0499   9.4673513   Err
       0.1D-04             -
281    203:  3  3  1  2  2  1   S 2   9.4672951    0.0562   9.4673513   Err
       0.1D-04             -
282    204:  3 K   3  2 K   2   S 3   9.4629368    2.1590   9.4650958   Err
       0.1D-04             -
283    205:  3 K  -3  2 K  -2   S 4   9.4628766    2.0712   9.4649478   Err
       0.1D-04             -
284    206:  3 K   3  2 K   2   S 5   9.4629909    2.2390   9.4652299   Err
       0.1D-04             -
285    207:  5  2  4  4  1  4   S 1  11.8014628    0.0243  11.8014871   Err
       0.1D-04             -
286    Maximum (obs-calc)/err in line 155   0.0038608
287
288    indep.par: 10 stepw:1.0000 lambda:0.400D-05 cond.no:0.188D+03
289
290    ------------------------------------------------------
291    Iteration :  1
292    Sigma:0.234649D-03  Sigma/OldSigma:0.227808  conv: 1
293
294                   Parameters     Change
295    BJ              0.836003965 {-0.000000196}
296    BK              0.887783553 { 0.000015385}
297    B-              0.079159050 { 0.000001391}
298    DJ              0.041484E-6 {-0.010876E-6}
299    DJK             0.691667E-6 { 0.399309E-6}
300    DK             -0.584252E-6 {-0.469180E-6}
301    dj              0.071811E-6 { 0.049888E-6}
302    dk             -1.750481E-6 {-1.169124E-6}
303   \F12             0.213835172 {     derived}
304   \F             161.461011707 {     derived}  160.498267942 {     derived}
```

```
 V1n          13459.345855 { 1064.276635}   13337.123862 {-1751.718908}
\rho           0.007126217 {     derived}    0.005326177 {     derived}
\beta          0.600686875 {     derived}    1.613791502 {     derived}
\gamma         1.215426274 {     derived}    2.512534198 {     derived}
 DK     (1)   -0.584252E-6  -0.469180E-6  80.304% Max. Change

 indep.par: 10 stepw:1.0000 lambda:0.160D-05 cond.no:0.187D+03

 ----------------------------------------------------
 Iteration :  2
 Sigma:0.334318D-04  Sigma/OldSigma:0.142476  conv: 1

                Parameters     Change
 BJ             0.836003991 { 0.000000026}
 BK             0.887784960 { 0.000001406}
 B-             0.079157661 {-0.000001389}
 DJ             0.079943E-6 { 0.038459E-6}
 DJK            0.358655E-6 {-0.333012E-6}
 DK            -0.243755E-6 { 0.340497E-6}
 dj             0.022244E-6 {-0.049567E-6}
 dk            -0.725863E-6 { 1.024618E-6}
\F12            0.213836168 {     derived}
\F            161.461012978 {     derived}  160.498267793 {     derived}
 V1n          13852.484184 {  393.138329}   13662.980166 {  325.856304}
\rho           0.007126224 {     derived}    0.005326176 {     derived}
\beta          0.600687170 {     derived}    1.613791552 {     derived}
\gamma         1.215427370 {     derived}    2.512532601 {     derived}
 dj     (1)    0.022244E-6  -0.049567E-6 222.837% Max. Change

 indep.par: 10 stepw:1.0000 lambda:0.640D-06 cond.no:0.187D+03

 ----------------------------------------------------
 Iteration :  3
 Sigma:0.275042D-04  Sigma/OldSigma:0.822696  conv: 1

                Parameters     Change
 BJ             0.836004007 { 0.000000015}
 BK             0.887785093 { 0.000000133}
 B-             0.079157566 {-0.000000095}
 DJ             0.083165E-6 { 0.003221E-6}
 DJK            0.336765E-6 {-0.021890E-6}
 DK            -0.216242E-6 { 0.027512E-6}
 dj             0.018968E-6 {-0.003275E-6}
 dk            -0.658447E-6 { 0.067417E-6}
\F12            0.213836240 {     derived}
\F            161.461013090 {     derived}  160.498267797 {     derived}
 V1n          13890.632765 {   38.148581}   13668.054650 {    5.074484}
\rho           0.007126225 {     derived}    0.005326175 {     derived}
\beta          0.600687184 {     derived}    1.613791556 {     derived}
\gamma         1.215427446 {     derived}    2.512532490 {     derived}
 dj     (1)    0.018968E-6  -0.003275E-6  17.268% Max. Change

 indep.par: 10 stepw:1.0000 lambda:0.256D-06 cond.no:0.187D+03

 ----------------------------------------------------
 Iteration :  4
 Sigma:0.275033D-04  Sigma/OldSigma:0.999968  conv: 1

                Parameters     Change
 BJ             0.836004007 { 0.000000000}
 BK             0.887785095 { 0.000000002}
 B-             0.079157564 {-0.000000002}
 DJ             0.083226E-6 { 0.000061E-6}
 DJK            0.336563E-6 {-0.000202E-6}
 DK            -0.215935E-6 { 0.000307E-6}
 dj             0.018935E-6 {-0.000033E-6}
 dk            -0.657853E-6 { 0.000594E-6}
\F12            0.213836241 {     derived}
\F            161.461013092 {     derived}  160.498267797 {     derived}
 V1n          13891.077119 {    0.444354}   13665.789726 {   -2.264924}
\rho           0.007126225 {     derived}    0.005326175 {     derived}
\beta          0.600687185 {     derived}    1.613791556 {     derived}
\gamma         1.215427447 {     derived}    2.512532488 {     derived}
```

```
 dj     (1)    0.018935E-6  -0.000033E-6   0.175% Max. Change

 indep.par: 10 stepw:1.0000 lambda:0.102D-06 cond.no:0.187D+03

 ----------------------------------------------------
 Iteration :  5
 Sigma:0.275033D-04  Sigma/OldSigma:1.000000  conv:-1
 DJ     (1)    0.083217E-6  -0.000009E-6   0.010% Max. Change

 indep.par: 10 stepw:1.0000 lambda:0.102D-05 cond.no:0.187D+03

 ----------------------------------------------------
 Iteration :  6
 Sigma:0.275033D-04  Sigma/OldSigma:1.000000  conv:-2
 DJ     (1)    0.083217E-6  -0.000009E-6   0.010% Max. Change

 indep.par: 10 stepw:1.0000 lambda:0.102D-04 cond.no:0.187D+03

 ----------------------------------------------------
 Iteration :  7
 Switching to better derivatives (takes more time)
 Sigma:0.275033D-04  Sigma/OldSigma:1.000000  conv:-3
 DJ     (1)    0.083217E-6  -0.000009E-6   0.010% Max. Change

 indep.par: 10 stepw:1.0000 lambda:0.102D-03 cond.no:0.186D+03

 ----------------------------------------------------
 Iteration :  8
 Sigma:0.275033D-04  Sigma/OldSigma:1.000000  conv:-4
 DJ     (1)    0.083217E-6  -0.000009E-6   0.010% Max. Change

 indep.par: 10 stepw:1.0000 lambda:0.102D-02 cond.no:0.176D+03

 ----------------------------------------------------
 Iteration :  9
 Sigma:0.275033D-04  Sigma/OldSigma:1.000000  conv:-5
 DJ     (1)    0.083217E-6  -0.000009E-6   0.010% Max. Change

 indep.par: 10 stepw:1.0000 lambda:0.102D-01 cond.no:0.117D+03

 ----------------------------------------------------
 Iteration : 10
 Sigma:0.275033D-04  Sigma/OldSigma:1.000000  conv:-6
 DJ     (1)    0.083217E-6  -0.000009E-6   0.010% Max. Change

 indep.par: 10 stepw:1.0000 lambda:0.102D+00 cond.no:0.272D+02

 ----------------------------------------------------
 Iteration : 11
 Sigma:0.275033D-04  Sigma/OldSigma:1.000000  conv:-7
 DJ     (1)    0.083219E-6  -0.000007E-6   0.008% Max. Change

 indep.par: 10 stepw:1.0000 lambda:0.102D+01 cond.no:0.400D+01

 ----------------------------------------------------
 Iteration : 12
 Sigma:0.275033D-04  Sigma/OldSigma:1.000000  conv: 1

                 Parameters     Change
 BJ               0.836004007 { 0.000000000}
 BK               0.887785095 {-0.000000000}
 B-               0.079157564 { 0.000000000}
 DJ               0.083224E-6 {-0.000002E-6}
 DJK              0.336563E-6 { 0.000000E-6}
 DK              -0.215935E-6 { 0.000000E-6}
 dj               0.018935E-6 {-0.000000E-6}
 dk              -0.657851E-6 { 0.000002E-6}
\F12              0.213836241 {     derived}
\F              161.461013092 {     derived}  160.498267797 {     derived}
 V1n            13891.077713 {    0.000594}   13666.183636 {    0.393910}
\rho              0.007126225 {     derived}    0.005326175 {     derived}
\beta             0.600687185 {     derived}    1.613791556 {     derived}
\gamma            1.215427447 {     derived}    2.512532488 {     derived}
```

```
 V1n_2  (1)   13666.183636      0.393910   0.003% Max. Change

 indep.par: 10 stepw:1.0000 lambda:0.410D+00 cond.no:0.833D+01

 ----------------------------------------------------
 Iteration : 13
 Sigma:0.275033D-04  Sigma/OldSigma:1.000000  conv:-1
 DJ     (1)    0.083222E-6  -0.000001E-6   0.002% Max. Change

 indep.par: 10 stepw:1.0000 lambda:0.410D+01 cond.no:0.176D+01

 ----------------------------------------------------
 Iteration : 14
 Sigma:0.275033D-04  Sigma/OldSigma:1.000000  conv:-2
 DJ     (1)    0.083224E-6  -0.000000E-6   0.000% Max. Change

 indep.par: 10 stepw:1.0000 lambda:0.410D+02 cond.no:0.108D+01

 ----------------------------------------------------
 Iteration : 15
 Sigma:0.275033D-04  Sigma/OldSigma:1.000000  conv:-3
 DJ     (1)    0.083224E-6  -0.000000E-6   0.000% Max. Change

 indep.par: 10 stepw:1.0000 lambda:0.410D+03 cond.no:0.101D+01

 ----------------------------------------------------
 Iteration : 16
 Sigma:0.275033D-04  Sigma/OldSigma:1.000000  conv:-4
 DJ     (1)    0.083224E-6  -0.000000E-6   0.000% Max. Change

 indep.par: 10 stepw:1.0000 lambda:0.410D+04 cond.no:0.100D+01

 ----------------------------------------------------
 Iteration : 17
 Sigma:0.275033D-04  Sigma/OldSigma:1.000000  conv:-5
 DJ     (1)    0.083224E-6  -0.000000E-6   0.000% Max. Change

 indep.par: 10 stepw:1.0000 lambda:0.410D+04 cond.no:0.187D+03

 Recalculation of the spectrum
##########################################################
  End at Cycle  17

      J K- K+  J K- K+    Sym   calc/GHz  diff/MHz     obs/GHz
   1:  9  3  7  9  2  7   S 1   2.1927739    0.0645   2.1928384   Err
   0.1D-04             -
   2:  9  3  7  9  2  7   S 2   2.1928552   -0.0168   2.1928384   Err
   0.1D-04             -
   3:  9 K   3  9 K   0   S 3   2.1926685    0.0333   2.1927017   Err
   0.1D-04             -
   4:  9 K  -3  9 K   0   S 4   2.1927350   -0.0332   2.1927017   Err
   0.1D-04             -
   5:  9 K   3  9 K   0   S 5   2.1927645   -0.0628   2.1927017   Err
   0.1D-04             -
   6:  3  2  2  3  1  2   S 1   2.2025621    0.0112   2.2025733   Err
   0.1D-04             -
   7:  3  2  2  3  1  2   S 2   2.2025848   -0.0115   2.2025733   Err
   0.1D-04             -
   8:  3 K   2  3 K  -1   S 3   2.2024824    0.0128   2.2024952   Err
   0.1D-04             -
   9:  3 K  -2  3 K   1   S 4   2.2024993   -0.0041   2.2024952   Err
   0.1D-04             -
  10:  3 K   2  3 K  -1   S 5   2.2025109   -0.0157   2.2024952   Err
  0.1D-04              -
  11:  4  2  2  4  1  3   S 1   2.2113939   -0.0035   2.2113904   Err
  0.1D-04              -
  12:  5  2  3  5  1  4   S 1   2.2205638   -0.0008   2.2205630   Err
  0.1D-04              -
  13:  3  2  1  3  1  2   S 1   2.3054534    0.0116   2.3054650   Err
  0.1D-04              -
  14:  2  0  2  1  1  0   S 1   2.3560385   -0.0250   2.3560135   Err
  0.1D-04              -
  15:  6  2  4  6  1  5   S 1   2.3764581   -0.0061   2.3764520   Err
```

```
      0.1D-04             -
510   16:  2  2  1  2  1  1   S 1   2.4258866    0.0016   2.4258882   Err
      0.1D-04             -
511   17:  5  1  4  5  0  5   S 1   2.5373377   -0.0121   2.5373256   Err
      0.1D-04             -
512   18:  1  1  0  0  0  0   S 1   2.6389628   -0.0086   2.6389542   Err
      0.1D-04             -
513   19:  2  2  1  2  1  2   S 1   2.9008335    0.0030   2.9008365   Err
      0.1D-04             -
514   20:  2  2  0  2  1  2   S 1   2.9218833    0.0061   2.9218894   Err
      0.1D-04             -
515   21:  2  2  0  2  1  2   S 2   2.9218919   -0.0024   2.9218894   Err
      0.1D-04             -
516   22:  2 K  -2  2 K   1   S 3   2.9220749    0.0189   2.9220938   Err
      0.1D-04             -
517   23:  2 K   2  2 K  -1   S 4   2.9221044    0.0210   2.9221255   Err
      0.1D-04             -
518   24:  2 K  -2  2 K   1   S 5   2.9220624   -0.0260   2.9220364   Err
      0.1D-04             -
519   25:  3  2  2  3  1  3   S 1   3.1506851    0.0110   3.1506960   Err
      0.1D-04             -
520   26:  3  2  2  3  1  3   S 2   3.1506862    0.0098   3.1506960   Err
      0.1D-04             -
521   27:  3 K   2  3 K   1   S 3   3.1506248    0.0039   3.1506288   Err
      0.1D-04             -
522   28:  3 K  -2  3 K  -1   S 4   3.1506335   -0.0047   3.1506288   Err
      0.1D-04             -
523   29:  3 K   2  3 K   1   S 5   3.1506185    0.0103   3.1506288   Err
      0.1D-04             -
524   30:  4  1  4  3  2  2   S 1   3.1834201    0.0075   3.1834276   Err
      0.1D-04             -
525   31:  2  1  2  1  1  1   S 1   3.1857166   -0.0113   3.1857052   Err
      0.1D-04             -
526   32:  7  3  5  7  2  5   S 1   3.2043140    0.0367   3.2043507   Err
      0.1D-04             -
527   33:  7  3  5  7  2  5   S 2   3.2043740   -0.0233   3.2043507   Err
      0.1D-04             -
528   34:  7 K   3  7 K  -2   S 3   3.2041957    0.0295   3.2042252   Err
      0.1D-04             -
529   35:  7 K  -3  7 K   2   S 4   3.2042478   -0.0226   3.2042252   Err
      0.1D-04             -
530   36:  7 K   3  7 K  -2   S 5   3.2042634   -0.0382   3.2042252   Err
      0.1D-04             -
531   37:  3  2  1  3  1  3   S 1   3.2535764   -0.0060   3.2535704   Err
      0.1D-04             -
532   38:  3  2  1  3  1  3   S 2   3.2535737   -0.0033   3.2535704   Err
      0.1D-04             -
533   39:  3 K  -2  3 K   1   S 3   3.2536066    0.0063   3.2536129   Err
      0.1D-04             -
534   40:  3 K   2  3 K  -1   S 4   3.2536193   -0.0064   3.2536129   Err
      0.1D-04             -
535   41:  3 K  -2  3 K   1   S 5   3.2535884    0.0245   3.2536129   Err
      0.1D-04             -
536   42:  2  0  2  1  0  1   S 1   3.3229836   -0.0169   3.3229667   Err
      0.1D-04             -
537   43:  4  2  3  4  1  4   S 1   3.4881720   -0.0143   3.4881577   Err
      0.1D-04             -
538   44:  2  1  1  1  1  0   S 1   3.5023475   -0.0267   3.5023209   Err
      0.1D-04             -
539   45:  6  3  4  6  2  4   S 1   3.6323683    0.0324   3.6324006   Err
      0.1D-04             -
540   46:  6  3  4  6  2  4   S 2   3.6324173   -0.0166   3.6324006   Err
      0.1D-04             -
541   47:  6 K   3  6 K  -2   S 3   3.6322188    0.0298   3.6322486   Err
      0.1D-04             -
542   48:  6 K  -3  6 K   2   S 4   3.6322581   -0.0095   3.6322486   Err
      0.1D-04             -
543   49:  6 K   3  6 K  -2   S 5   3.6322775   -0.0289   3.6322486   Err
      0.1D-04             -
544   50:  4  2  2  4  1  4   S 1   3.7822880    0.0037   3.7822917   Err
      0.1D-04             -
545   51:  3  0  3  2  1  1   S 1   3.7878945    0.0008   3.7878952   Err
      0.1D-04             -
```

```
546     52:  3  0  3  2  1  1   S 2   3.7878610    0.0342   3.7878952   Err
        0.1D-04              -
547     53:  3 K   0  2 K  -1   S 3   3.7878660   -0.0203   3.7878458   Err
        0.1D-04              -
548     54:  3 K   0  2 K   1   S 4   3.7878415    0.0043   3.7878458   Err
        0.1D-04              -
549     55:  3 K   0  2 K  -1   S 5   3.7878237    0.0221   3.7878458   Err
        0.1D-04              -
550     56:  5  2  4  4  3  2   S 1   3.8082504   -0.0256   3.8082249   Err
        0.1D-04              -
551     57:  5  3  3  5  2  3   S 1   3.9730386    0.0241   3.9730628   Err
        0.1D-04              -
552     58:  5  3  3  5  2  3   S 2   3.9730779   -0.0151   3.9730628   Err
        0.1D-04              -
553     59:  5 K   3  5 K  -2   S 3   3.9727630    0.0147   3.9727778   Err
        0.1D-04              -
554     60:  5 K  -3  5 K   2   S 4   3.9727827   -0.0372   3.9727455   Err
        0.1D-04              -
555     61:  5 K   3  5 K  -2   S 5   3.9728219    0.0136   3.9728356   Err
        0.1D-04              -
556     62:  2  1  2  1  0  1   S 1   3.9943458   -0.0032   3.9943426   Err
        0.1D-04              -
557     63:  2  1  2  1  0  1   S 2   3.9943382    0.0044   3.9943426   Err
        0.1D-04              -
558     64:  2 K   1  1 K   0   S 3   3.9943209   -0.0216   3.9942993   Err
        0.1D-04              -
559     65:  2 K  -1  1 K   0   S 4   3.9943087   -0.0093   3.9942993   Err
        0.1D-04              -
560     66:  2 K   1  1 K   0   S 5   3.9943179   -0.0186   3.9942993   Err
        0.1D-04              -
561     67:  4  3  2  4  2  2   S 1   4.2109515    0.0160   4.2109674   Err
        0.1D-04              -
562     68:  4  3  2  4  2  2   S 2   4.2109824   -0.0149   4.2109674   Err
        0.1D-04              -
563     69:  4 K   3  4 K  -2   S 3   4.2101152    0.0124   4.2101275   Err
        0.1D-04              -
564     70:  4 K  -3  4 K   2   S 4   4.2100847   -0.0397   4.2100449   Err
        0.1D-04              -
565     71:  4 K   3  4 K  -2   S 5   4.2102081    0.0030   4.2102111   Err
        0.1D-04              -
566     72:  3  0  3  2  1  2   S 1   4.2628414   -0.0197   4.2628217   Err
        0.1D-04              -
567     73:  3  3  1  3  2  1   S 1   4.3484262    0.0350   4.3484612   Err
        0.1D-04              -
568     74:  3  3  1  3  2  1   S 2   4.3484441   -0.0210   4.3484232   Err
        0.1D-04              -
569     75:  3 K   3  3 K  -2   S 3   4.3459259    0.0172   4.3459431   Err
        0.1D-04              -
570     76:  3 K  -3  3 K   2   S 4   4.3458241   -0.0571   4.3457671   Err
        0.1D-04              -
571     77:  3 K   3  3 K  -2   S 5   4.3460822    0.0285   4.3461107   Err
        0.1D-04              -
572     78:  3 K  -3  3 K  -2   S 3   4.3525130    0.0143   4.3525273   Err
        0.1D-04              -
573     79:  3 K   3  3 K   2   S 4   4.3526617    0.0148   4.3526765   Err
        0.1D-04              -
574     80:  3 K  -3  3 K  -2   S 5   4.3524194   -0.0468   4.3523726   Err
        0.1D-04              -
575     81:  5  1  5  4  2  3   S 1   4.4004966   -0.0223   4.4004742   Err
        0.1D-04              -
576     82:  3  3  0  3  2  2   S 1   4.4530856    0.0241   4.4531097   Err
        0.1D-04              -
577     83:  3  3  0  3  2  2   S 2   4.4531185   -0.0089   4.4531097   Err
        0.1D-04              -
578     84:  3 K  -3  3 K   2   S 3   4.4554948    0.0161   4.4555108   Err
        0.1D-04              -
579     85:  3 K   3  3 K  -2   S 4   4.4556475    0.0274   4.4556749   Err
        0.1D-04              -
580     86:  3 K  -3  3 K   2   S 5   4.4553894   -0.0446   4.4553448   Err
        0.1D-04              -
581     87:  2  1  1  1  0  1   S 1   4.4692927   -0.0128   4.4692799   Err
        0.1D-04              -
582     88:  4  3  2  4  2  3   S 1   4.5050675    0.0078   4.5050753   Err
```

```
       0.1D-04             -
583     89:  4  3  2  4  2  3   S 2   4.5050873   -0.0120   4.5050753   Err
       0.1D-04             -
584     90:  4 K   3  4 K   2   S 3   4.5042749    0.0073   4.5042822   Err
       0.1D-04             -
585     91:  4 K  -3  4 K  -2   S 4   4.5042384   -0.0317   4.5042067   Err
       0.1D-04             -
586     92:  4 K   3  4 K   2   S 5   4.5043516    0.0090   4.5043606   Err
       0.1D-04             -
587     93:  4  3  1  4  2  3   S 1   4.5173191    0.0088   4.5173279   Err
       0.1D-04             -
588     94:  4  3  1  4  2  3   S 2   4.5173408   -0.0129   4.5173279   Err
       0.1D-04             -
589     95:  4 K  -3  4 K   2   S 3   4.5180609    0.0105   4.5180713   Err
       0.1D-04             -
590     96:  4 K   3  4 K  -2   S 4   4.5181441    0.0033   4.5181475   Err
       0.1D-04             -
591     97:  4 K  -3  4 K   2   S 5   4.5180202   -0.0333   4.5179869   Err
       0.1D-04             -
592     98:  5  2  3  5  1  5   S 1   4.5472481    0.0009   4.5472490   Err
       0.1D-04             -
593     99:  4  1  3  3  2  1   S 1   4.6514229   -0.0123   4.6514106   Err
       0.1D-04             -
594    100:  4  1  3  3  2  1   S 2   4.6513598    0.0508   4.6514106   Err
       0.1D-04             -
595    101:  4 K  -1  3 K  -2   S 3   4.6513946   -0.0361   4.6513585   Err
       0.1D-04             -
596    102:  4 K   1  3 K   2   S 4   4.6513300    0.0285   4.6513585   Err
       0.1D-04             -
597    103:  4 K  -1  3 K  -2   S 5   4.6513331    0.0254   4.6513585   Err
       0.1D-04             -
598    104:  5  3  2  5  2  4   S 1   4.6554805    0.0061   4.6554866   Err
       0.1D-04             -
599    105:  5  3  2  5  2  4   S 2   4.6554943   -0.0077   4.6554866   Err
       0.1D-04             -
600    106:  5 K  -3  5 K   2   S 3   4.6556588    0.0067   4.6556654   Err
       0.1D-04             -
601    107:  5 K   3  5 K  -2   S 4   4.6556937    0.0077   4.6557014   Err
       0.1D-04             -
602    108:  5 K  -3  5 K   2   S 5   4.6556514   -0.0349   4.6556164   Err
       0.1D-04             -
603    109:  3  1  3  2  1  2   S 1   4.7661855    0.0001   4.7661856   Err
       0.1D-04             -
604    110:  3  1  3  2  1  2   S 2   4.7661643    0.0213   4.7661856   Err
       0.1D-04             -
605    111:  3 K   1  2 K   1   S 3   4.7661683   -0.0171   4.7661512   Err
       0.1D-04             -
606    112:  3 K  -1  2 K  -1   S 4   4.7661396    0.0116   4.7661512   Err
       0.1D-04             -
607    113:  3 K   1  2 K   1   S 5   4.7661546   -0.0034   4.7661512   Err
       0.1D-04             -
608    114:  6  3  3  6  2  5   S 1   4.9129959    0.0008   4.9129967   Err
       0.1D-04             -
609    115:  3  0  3  2  0  2   S 1   4.9342036   -0.0110   4.9341926   Err
       0.1D-04             -
610    116:  3  0  3  2  0  2   S 2   4.9341739    0.0187   4.9341926   Err
       0.1D-04             -
611    117:  3 K   0  2 K   0   S 3   4.9341784   -0.0217   4.9341567   Err
       0.1D-04             -
612    118:  3 K   0  2 K   0   S 4   4.9341561    0.0006   4.9341567   Err
       0.1D-04             -
613    119:  3 K   0  2 K   0   S 5   4.9341415    0.0152   4.9341567   Err
       0.1D-04             -
614    120:  4  0  4  3  1  2   S 1   5.0452361    0.0004   5.0452365   Err
       0.1D-04             -
615    121:  3  1  2  2  1  1   S 1   5.2393615   -0.0197   5.2393418   Err
       0.1D-04             -
616    122:  6  2  5  5  3  3   S 1   5.3309654   -0.0436   5.3309218   Err
       0.1D-04             -
617    123:  7  3  4  7  2  6   S 1   5.3445074   -0.0184   5.3444889   Err
       0.1D-04             -
618    124:  3  1  3  2  0  2   S 1   5.4375476    0.0048   5.4375524   Err
       0.1D-04             -
```

```
619    125:  3  1  3  2  0  2   S 2   5.4375396    0.0129   5.4375524   Err
       0.1D-04                 -
620    126:  3 K   1  2 K   0   S 3   5.4375192   -0.0076   5.4375116   Err
       0.1D-04                 -
621    127:  3 K  -1  2 K   0   S 4   5.4374966    0.0150   5.4375116   Err
       0.1D-04                 -
622    128:  3 K   1  2 K   0   S 5   5.4375256   -0.0140   5.4375116   Err
       0.1D-04                 -
623    129:  6  1  6  5  2  4   S 1   5.5181138   -0.0154   5.5180985   Err
       0.1D-04                 -
624    130:  8  4  4  7  5  2   S 1   5.6601589    0.0033   5.6601621   Err
       0.1D-04                 -
625    131:  7  4  4  7  3  4   S 1   5.8574134    0.0350   5.8574485   Err
       0.1D-04                 -
626    132:  7  4  4  7  3  4   S 2   5.8574631   -0.0147   5.8574485   Err
       0.1D-04                 -
627    133:  7 K   4  7 K  -3   S 3   5.8564450    0.0349   5.8564799   Err
       0.1D-04                 -
628    134:  2  2  1  1  1  0   S 1   5.9282341   -0.0041   5.9282300   Err
       0.1D-04                 -
629    135:  2  2  0  1  1  0   S 1   5.9492839   -0.0012   5.9492827   Err
       0.1D-04                 -
630    136:  2  2  0  1  1  0   S 2   5.9492797    0.0030   5.9492827   Err
       0.1D-04                 -
631    137:  2 K   2  1 K   1   S 4   5.9494612    0.0178   5.9494790   Err
       0.1D-04                 -
632    138:  2 K  -2  1 K  -1   S 5   5.9494292   -0.0177   5.9494115   Err
       0.1D-04                 -
633    139:  4  0  4  3  1  3   S 1   5.9933590   -0.0197   5.9933394   Err
       0.1D-04                 -
634    140:  6  4  3  6  3  3   S 1   6.0304890    0.0187   6.0305077   Err
       0.1D-04                 -
635    141:  2  2  1  1  1  1   S 1   6.0865501   -0.0042   6.0865458   Err
       0.1D-04                 -
636    142:  2  2  1  1  1  1   S 2   6.0865433    0.0026   6.0865458   Err
       0.1D-04                 -
637    143:  2 K  -2  1 K  -1   S 4   6.0862901   -0.0251   6.0862650   Err
       0.1D-04                 -
638    144:  2 K   2  1 K   1   S 5   6.0863213    0.0113   6.0863326   Err
       0.1D-04                 -
639    145:  2  2  0  1  1  1   S 1   6.1075999   -0.0108   6.1075891   Err
       0.1D-04                 -
640    146:  5  4  2  5  3  2   S 1   6.1242316   -0.0008   6.1242308   Err
       0.1D-04                 -
641    147:  5  4  2  5  3  3   S 2   6.1722556   -0.0435   6.1722122   Err
       0.1D-04                 -
642    148:  4 K  -4  4 K  -3   S 3   6.1724625    0.0216   6.1724841   Err
       0.1D-04                 -
643    149:  4 K   4  4 K   3   S 4   6.1726112    0.0238   6.1726350   Err
       0.1D-04                 -
644    150:  4 K  -4  4 K  -3   S 5   6.1723834   -0.0651   6.1723183   Err
       0.1D-04                 -
645    151:  5  4  1  5  3  3   S 1   6.1733463    0.0263   6.1733726   Err
       0.1D-04                 -
646    152:  5  4  1  5  3  3   S 2   6.1734080   -0.0355   6.1733726   Err
       0.1D-04                 -
647    153:  5 K  -4  5 K   3   S 3   6.1771944    0.0135   6.1772079   Err
       0.1D-04                 -
648    154:  5 K   4  5 K  -3   S 4   6.1774179    0.0484   6.1774663   Err
       0.1D-04                 -
649    155:  5 K  -4  5 K   3   S 5   6.1770417   -0.0698   6.1769719   Err
       0.1D-04                 -
650    156:  6  4  2  6  3  4   S 1   6.1747703    0.0154   6.1747857   Err
       0.1D-04                 -
651    157:  6  4  2  6  3  4   S 2   6.1748096   -0.0239   6.1747857   Err
       0.1D-04                 -
652    158:  6 K  -4  6 K   3   S 3   6.1770662    0.0162   6.1770824   Err
       0.1D-04                 -
653    159:  6 K   4  6 K  -3   S 4   6.1772517    0.0215   6.1772732   Err
       0.1D-04                 -
654    160:  6 K  -4  6 K   3   S 5   6.1769503   -0.0525   6.1768979   Err
       0.1D-04                 -
655    161:  4 K   4  4 K   3   S 3   6.1777847    0.0183   6.1778030   Err
```

```
      0.1D-04                -
656   162:  4 K  -4  4 K  -3   S 4   6.1777069   -0.0538   6.1776531   Err
      0.1D-04                -
657   163:  4 K   4  4 K   3   S 5   6.1779347    0.0117   6.1779464   Err
      0.1D-04                -
658   164:  4  4  0  4  3  2   S 1   6.1813738    0.0163   6.1813901   Err
      0.1D-04                -
659   165:  4  4  0  4  3  2   S 2   6.1815325    0.0258   6.1815584   Err
      0.1D-04                -
660   166:  7  4  3  7  3  5   S 1   6.2037008    0.0086   6.2037094   Err
      0.1D-04                -
661   167:  7  4  3  7  3  5   S 2   6.2037327   -0.0232   6.2037094   Err
      0.1D-04                -
662   168:  7 K  -4  7 K   3   S 3   6.2045263    0.0202   6.2045465   Err
      0.1D-04                -
663   169:  8  4  4  8  3  6   S 1   6.2889731    0.0018   6.2889749   Err
      0.1D-04                -
664   170:  3  1  2  2  0  2   S 1   6.3856706   -0.0160   6.3856546   Err
      0.1D-04                -
665   171:  5  1  4  4  2  2   S 1   6.4330649   -0.0278   6.4330371   Err
      0.1D-04                -
666   172:  9  4  5  9  3  7   S 1   6.4716732    0.0101   6.4716833   Err
      0.1D-04                -
667   173: 10  4  7 10  3  8   S 1   6.4941381   -0.0026   6.4941356   Err
      0.1D-04                -
668   174:  4  0  4  3  0  3   S 1   6.4967031   -0.0239   6.4966793   Err
      0.1D-04                -
669   175:  4  2  3  3  2  2   S 1   6.6715921   -0.0061   6.6715860   Err
      0.1D-04                -
670   176:  4  1  4  3  0  3   S 1   6.8374493   -0.0007   6.8374485   Err
      0.1D-04                -
671   177:  4  2  2  3  2  1   S 1   6.8628168   -0.0266   6.8627903   Err
      0.1D-04                -
672   178:  3  2  2  2  1  1   S 1   7.4419236   -0.0003   7.4419234   Err
      0.1D-04                -
673   179:  3  2  2  2  1  1   S 2   7.4419129    0.0105   7.4419234   Err
      0.1D-04                -
674   180:  3 K   2  2 K  -1   S 3   7.4418316   -0.0038   7.4418278   Err
      0.1D-04                -
675   181:  3 K  -2  2 K   1   S 4   7.4418155    0.0123   7.4418278   Err
      0.1D-04                -
676   182:  3 K   2  2 K  -1   S 5   7.4418262    0.0016   7.4418278   Err
      0.1D-04                -
677   183:  3  2  1  2  1  1   S 1   7.5448150   -0.0066   7.5448083   Err
      0.1D-04                -
678   184:  5  0  5  4  1  4   S 1   7.6780152   -0.0137   7.6780016   Err
      0.1D-04                -
679   185:  5  0  5  4  1  4   S 2   7.6779177    0.0839   7.6780016   Err
      0.1D-04                -
680   186:  5 K   0  4 K   1   S 3   7.6779226   -0.0230   7.6778995   Err
      0.1D-04                -
681   187:  5 K   0  4 K  -1   S 4   7.6779380   -0.0385   7.6778995   Err
      0.1D-04                -
682   188:  5 K   0  4 K   1   S 5   7.6777121    0.1874   7.6778995   Err
      0.1D-04                -
683   189:  5  1  5  4  1  4   S 1   7.8886686   -0.0183   7.8886502   Err
      0.1D-04                -
684   190:  3  2  2  2  1  2   S 1   7.9168705   -0.0050   7.9168655   Err
      0.1D-04                -
685   191:  3  2  2  2  1  2   S 2   7.9168505    0.0150   7.9168655   Err
      0.1D-04                -
686   192:  3 K   2  2 K   1   S 3   7.9167931    0.0008   7.9167938   Err
      0.1D-04                -
687   193:  3 K  -2  2 K  -1   S 4   7.9167731    0.0208   7.9167938   Err
      0.1D-04                -
688   194:  3 K   2  2 K   1   S 5   7.9167731    0.0207   7.9167938   Err
      0.1D-04                -
689   195:  4  1  3  3  0  3   S 1   8.4083434   -0.0219   8.4083214   Err
      0.1D-04                -
690   196:  4  2  2  3  1  2   S 1   9.1682703   -0.0219   9.1682484   Err
      0.1D-04                -
691   197:  3  3  0  2  2  0   S 1   9.4480728    0.0105   9.4480833   Err
      0.1D-04                -
```

```
 198:  3  3  0  2  2  0   S 2   9.4480772    0.0061   9.4480833   Err
0.1D-04              -
 199:  3 K  -3  2 K  -2   S 3   9.4502130    0.0102   9.4502232   Err
0.1D-04              -
 200:  3 K   3  2 K   2   S 4   9.4503161    0.0547   9.4503708   Err
0.1D-04              -
 201:  3 K  -3  2 K  -2   S 5   9.4501001   -0.0265   9.4500736   Err
0.1D-04              -
 202:  3  3  1  2  2  1   S 1   9.4673545   -0.0033   9.4673513   Err
0.1D-04              -
 203:  3  3  1  2  2  1   S 2   9.4673399    0.0114   9.4673513   Err
0.1D-04              -
 204:  3 K   3  2 K   2   S 3   9.4650965   -0.0006   9.4650958   Err
0.1D-04              -
 205:  3 K  -3  2 K  -2   S 4   9.4649831   -0.0353   9.4649478   Err
0.1D-04              -
 206:  3 K   3  2 K   2   S 5   9.4651991    0.0308   9.4652299   Err
0.1D-04              -
 207:  5  2  4  4  1  4   S 1  11.8014899   -0.0028  11.8014871   Err
0.1D-04              -
 Maximum (obs-calc)/err in line 188   0.0001874

 RMS deviations (MHz), B and V sorted
  B  V   n splittings MHz
  B  V   n abs. freq. MHz
  1  1 207          0.026763          0.028055

             Parameters  and  Errors
 BJ            0.836004007 { 0.000000728}
 BK            0.887785095 { 0.000001180}
 B-            0.079157564 { 0.000000513}
 DJ            0.083224E-6 { 0.017181E-6}
 DJK           0.336563E-6 { 0.062612E-6}
 DK           -0.215935E-6 { 0.074721E-6}
 dj            0.018935E-6 { 0.007076E-6}
 dk           -0.657851E-6 { 0.163329E-6}
\F12           0.213836241 {     derived}
\F           161.461013092 {     derived}  160.498267797 {     derived}
 V1n          13891.077713 {    4.228224}   13666.183636 {   98.291004}
\rho           0.007126225 {     derived}    0.005326175 {     derived}
\beta          0.600687185 {     derived}    1.613791556 {     derived}
\gamma         1.215427447 {     derived}    2.512532488 {     derived}
 F0          160.398000000 {     fixed  }  159.647000000 {     fixed  }
 epsil         1.273000000 {     fixed  }    2.420000000 {     fixed  }
 delta         0.992000000 {     fixed  }    1.592000000 {     fixed  }

 Standard Deviation       0.027503 MHz

------------------------------------ B =  1
 Rotational Constants and Errors (in GHz)
      B_z       1.723789102       0.000001310
      B_x       0.915161571       0.000000918
      B_y       0.756846443       0.000000863
 Ray's kappa   -0.67254
 F0(calc)     160.398000000       0.000000000
 I_alpha        3.150781618       0.000000000
 <(i,x)  <(i,y)  <(i,z)       75.7813       36.8435       56.8374
d<(i,x) d<(i,y) d<(i,z)        0.0000        0.0000        0.0000

 F0(calc)     159.647000000       0.000000000
 I_alpha        3.165603300       0.000000000
 <(i,x)  <(i,y)  <(i,z)      138.6412       48.6671       91.2149
d<(i,x) d<(i,y) d<(i,z)        0.0000        0.0000        0.0000

 V1n_1      5.542975 kj +/- 0.001687 kj         1.323884 kcal +/- 0.000403 kcal
          463.356412 cm +/-   0.1410 cm    s=  38.237171
 V1n_2      5.453235 kj +/- 0.039221 kj         1.302451 kcal +/- 0.009368 kcal
          455.854754 cm +/-   3.2786 cm    s=  37.618118

F(calc)       161.461013092
F(calc)       160.498267797

 Errors of fitted linear combinations
```

```
      0.000000728    0.000001180    0.000000513    4.228223669    0.000000017
      0.000000063    0.000000075    0.000000007    0.000000163   98.291003931

 Correlation Matrix of fitted linear combinations
 BJ      1.000
 BK     -0.120  1.000
 B-      0.066 -0.221  1.000
 V1n_1  -0.064 -0.029  0.041  1.000
 DJ      0.755 -0.121  0.076 -0.109  1.000
 DJK    -0.082  0.375 -0.277  0.076 -0.415  1.000
 DK      0.099  0.427 -0.022 -0.188  0.382 -0.573  1.000
 dj      0.000  0.174  0.193  0.072 -0.339  0.819 -0.531  1.000
 dk      0.014 -0.301  0.353 -0.053  0.359 -0.910  0.446 -0.807  1.000
 V1n_2  -0.007 -0.009  0.045 -0.018 -0.050 -0.032  0.025 -0.017  0.040  1.000
 strongest correlation between  9 and  6 (-0.9104)

 Freedom Cofreedom Matrix of linear comb.
 BJ      0.573
 BK      0.996  0.377
 B-      0.996  0.976  0.421
 V1n_1   0.999  0.991  0.999  0.961
 DJ      0.629  0.992  0.997  0.997  0.516
 DJK     0.998  0.722  0.944  0.993  0.952  0.186
 DK      0.997  0.623  0.992  0.977  0.950  0.681  0.321
 dj      0.999  0.978  0.677  0.999  0.970  0.720  0.914  0.250
 dk      0.992  0.954  0.867  0.997  0.955  0.619  0.914  0.690  0.256
 V1n_2   0.998  0.999  0.999  1.000  0.996  0.999  0.999  1.000  0.999  0.991
 minimum cofreedom between  9 and  6 ( 0.6195)

 Eigenvalues and Eigenvector Matrix of SVD-FIT

0.168073D-01   0.019-0.102 0.061-0.012 0.111-0.686 0.244-0.462 0.478 0.004
0.801145D-01  -0.012 0.638-0.349-0.039 0.049 0.138 0.614-0.218-0.158-0.004
0.127909D+00   0.160 0.247 0.661-0.020 0.164-0.130 0.353 0.547 0.086 0.011
0.175766D+00   0.654-0.243-0.211-0.042 0.644-0.001 0.032-0.006-0.224-0.026
0.369627D+00   0.187 0.405 0.171 0.077 0.236 0.429-0.376-0.256 0.564-0.009
0.933231D+00   0.011 0.082 0.010 0.895 0.021-0.127-0.035-0.006-0.133-0.396
0.982462D+00  -0.152-0.025-0.011-0.383 0.102 0.033 0.002 0.039 0.061-0.902
0.167870D+01   0.638 0.139 0.202-0.127-0.611-0.095-0.095-0.242-0.203-0.157
0.249281D+01   0.283-0.095-0.505 0.089-0.300 0.029 0.152 0.488 0.541-0.051
0.314257D+01  -0.027 0.513-0.259-0.137 0.113-0.531-0.513 0.274-0.122 0.048
```

```
 Rotational, Centrifugal Distortion, Internal Rotation Calculation (V2.5e)
                       Holger Hartwig 08-Nov-96 (hartwig@phc.uni-kiel.de)

 Please cite: H.Hartwig and H.Dreizler, Z.Naturforsch, 51a (1996) 923.

 Calculation date and time:
 Modified version to include Dc3- and Dc3K

 Type help now for the list of parameters :
sBT-Si Me6Me7

   nzyk       200   print        4   eval         0   dfreq        0
   orger        0   ints         0   maxm         8   woods       33
   ndata     5000   nfold        3   spin         0   ntop         2
   adjf        16   maxvm        0   aprint      10   xprint       4
   ncycl      200   svderr       0   fitscl       0   reduct       0
   rofit    .0000000D+00   eps      .1000000D-11   defer    .1000000D-04
   weigf    .0000000D+00   convg    .1000000D+01   lambda   .1000000D-04
   freq_l   .6000000D+01   freq_h   .4000000D+02   limit    .1000000D+00
   temp     .2730000D+03
 Using Watson A Reduction

 assumed sizeb           1
 \\ set (adj  or  8)
 \\ set (adj  or  1)
 \\ set (adj  or  2)
 \\ adj  1: adjust F according to rho, beta and gamma
 \\ adj  2: adjust F12 according to rho, beta and gamma
 \\ adj  8: adjust rho according to F0 = 1/(2 I_alpha)
 \\ adj 16: adjust beta and gamma  according delta + epsil
 new adj :  27
 BJ            0.836004161
 BK            0.887768168
 B-            0.079157659
 DJ            0.052360E-6
 DJK           0.292358E-6
 DK           -0.115072E-6
 dj            0.021923E-6
 dk           -0.581357E-6
\F12          -0.353875962
\F           160.498761142                161.296324790
 V1n          15088.842770                 17621.743930
\rho           0.005326176                   0.006458854
\beta          1.613791130                   0.913402443
\gamma         2.512532580                   0.426131191
 F0          159.647000000                160.297000000
 epsil         2.420000000                   0.502000000
 delta         1.592000000                   1.195000000

 fit  0.1D+00 0.1D+01   BJ          1.00
 fit  0.1D+00 0.1D+01   BK          1.00
 fit  0.1D+00 0.1D+01   B-          1.00
 fit  0.1D+00 0.1D+01   V1n_1       1.00
 fit  0.1D+00 0.1D+01   DJ          1.00
 fit  0.1D+00 0.1D+01   DJK         1.00
 fit  0.1D+00 0.1D+01   DK          1.00
 fit  0.1D+00 0.1D+01   dj          1.00
 fit  0.1D+00 0.1D+01   dk          1.00
 fit  0.1D+00 0.1D+01   V1n_2       1.00

  S     0   0
  S     0   1
  S     1   0
  S    -1   1
  S     1   1

  V  0  0

   ndata  207   Data Points 207   Splittings   0
   Effective Data Points  92.0

  \\  Maximal K = J =  10
  \\ B=  1    adj= 27
```

```
 \\ (1) calculate torsional integrals
 \\ (32)use torsional integrals in rigid rotor H_rr
Sigma:0.970567D-03  Sigma/OldSigma:0.000000  conv: 1

      J K- K+  J K- K+    Sym   calc/GHz  diff/MHz     obs/GHz
  1:  9  3  7  9  2  7   S 1   2.1926511    0.1873   2.1928384   Err
  0.1D-04              -
  2:  9  3  7  9  2  7   S 2   2.1926721    0.0297   2.1927017   Err
  0.1D-04              -
  3:  9  3  7  9  2  7   S 3   2.1926959    0.1426   2.1928384   Err
  0.1D-04              -
  4:  9  3  7  9  2  7   S 4   2.1927163   -0.0146   2.1927017   Err
  0.1D-04              -
  5:  9  3  7  9  2  7   S 5   2.1927174   -0.0157   2.1927017   Err
  0.1D-04              -
  6:  3  2  2  3  1  2   S 1   2.2024911    0.0822   2.2025733   Err
  0.1D-04              -
  7:  3  2  2  3  1  2   S 2   2.2024932    0.0020   2.2024952   Err
  0.1D-04              -
  8:  3  2  2  3  1  2   S 3   2.2025043    0.0690   2.2025733   Err
  0.1D-04              -
  9:  3  2  2  3  1  2   S 4   2.2025057   -0.0105   2.2024952   Err
  0.1D-04              -
 10:  3  2  2  3  1  2   S 5   2.2025071   -0.0119   2.2024952   Err
 0.1D-04               -
 11:  4  2  2  4  1  3   S 1   2.2113347    0.0557   2.2113904   Err
 0.1D-04               -
 12:  5  2  3  5  1  4   S 1   2.2205165    0.0465   2.2205630   Err
 0.1D-04               -
 13:  3  2  1  3  1  2   S 1   2.3053878    0.0772   2.3054650   Err
 0.1D-04               -
 14:  2  0  2  1  1  0   S 1   2.3560490   -0.0355   2.3560135   Err
 0.1D-04               -
 15:  6  2  4  6  1  5   S 1   2.3764274    0.0246   2.3764520   Err
 0.1D-04               -
 16:  2  2  1  2  1  1   S 1   2.4258181    0.0701   2.4258882   Err
 0.1D-04               -
 17:  5  1  4  5  0  5   S 1   2.5373547   -0.0292   2.5373256   Err
 0.1D-04               -
 18:  1  1  0  0  0  0   S 1   2.6389386    0.0156   2.6389542   Err
 0.1D-04               -
 19:  2  2  1  2  1  2   S 1   2.9007734    0.0631   2.9008365   Err
 0.1D-04               -
 20:  2  2  0  2  1  2   S 1   2.9218244    0.0651   2.9218894   Err
 0.1D-04               -
 21:  2  2  0  2  1  2   S 2   2.9218273    0.2664   2.9220938   Err
 0.1D-04               -
 22:  2  2  0  2  1  2   S 3   2.9218295    0.0600   2.9218894   Err
 0.1D-04               -
 23:  2  2  0  2  1  2   S 4   2.9218346    0.2909   2.9221255   Err
 0.1D-04               -
 24:  2  2  0  2  1  2   S 5   2.9218303    0.2061   2.9220364   Err
 0.1D-04               -
 25:  3  2  2  3  1  3   S 1   3.1506302    0.0658   3.1506960   Err
 0.1D-04               -
 26:  3  2  2  3  1  3   S 2   3.1506239    0.0049   3.1506288   Err
 0.1D-04               -
 27:  3  2  2  3  1  3   S 3   3.1506318    0.0642   3.1506960   Err
 0.1D-04               -
 28:  3  2  2  3  1  3   S 4   3.1506263    0.0025   3.1506288   Err
 0.1D-04               -
 29:  3  2  2  3  1  3   S 5   3.1506248    0.0040   3.1506288   Err
 0.1D-04               -
 30:  4  1  4  3  2  2   S 1   3.1834588   -0.0312   3.1834276   Err
 0.1D-04               -
 31:  2  1  2  1  1  1   S 1   3.1857059   -0.0007   3.1857052   Err
 0.1D-04               -
 32:  7  3  5  7  2  5   S 1   3.2041797    0.1711   3.2043507   Err
 0.1D-04               -
 33:  7  3  5  7  2  5   S 2   3.2041930    0.0322   3.2042252   Err
 0.1D-04               -
 34:  7  3  5  7  2  5   S 3   3.2042142    0.1365   3.2043507   Err
 0.1D-04               -
```

```
113     35:  7  3  5  7  2  5   S 4   3.2042265   -0.0013   3.2042252   Err
        0.1D-04                 -
114     36:  7  3  5  7  2  5   S 5   3.2042285   -0.0033   3.2042252   Err
        0.1D-04                 -
115     37:  3  2  1  3  1  3   S 1   3.2535269    0.0435   3.2535704   Err
        0.1D-04                 -
116     38:  3  2  1  3  1  3   S 2   3.2535213    0.0916   3.2536129   Err
        0.1D-04                 -
117     39:  3  2  1  3  1  3   S 3   3.2535262    0.0442   3.2535704   Err
        0.1D-04                 -
118     40:  3  2  1  3  1  3   S 4   3.2535222    0.0907   3.2536129   Err
        0.1D-04                 -
119     41:  3  2  1  3  1  3   S 5   3.2535189    0.0940   3.2536129   Err
        0.1D-04                 -
120     42:  2  0  2  1  0  1   S 1   3.3229744   -0.0077   3.3229667   Err
        0.1D-04                 -
121     43:  4  2  3  4  1  4   S 1   3.4881241    0.0336   3.4881577   Err
        0.1D-04                 -
122     44:  2  1  1  1  1  0   S 1   3.5023424   -0.0215   3.5023209   Err
        0.1D-04                 -
123     45:  6  3  4  6  2  4   S 1   3.6322363    0.1644   3.6324006   Err
        0.1D-04                 -
124     46:  6  3  4  6  2  4   S 2   3.6322441    0.0046   3.6322486   Err
        0.1D-04                 -
125     47:  6  3  4  6  2  4   S 3   3.6322649    0.1357   3.6324006   Err
        0.1D-04                 -
126     48:  6  3  4  6  2  4   S 4   3.6322713   -0.0227   3.6322486   Err
        0.1D-04                 -
127     49:  6  3  4  6  2  4   S 5   3.6322742   -0.0255   3.6322486   Err
        0.1D-04                 -
128     50:  4  2  2  4  1  4   S 1   3.7822541    0.0377   3.7822917   Err
        0.1D-04                 -
129     51:  3  0  3  2  1  1   S 1   3.7878957   -0.0005   3.7878952   Err
        0.1D-04                 -
130     52:  3  0  3  2  1  1   S 2   3.7878930   -0.0472   3.7878458   Err
        0.1D-04                 -
131     53:  3  0  3  2  1  1   S 3   3.7878784    0.0169   3.7878952   Err
        0.1D-04                 -
132     54:  3  0  3  2  1  1   S 4   3.7878755   -0.0297   3.7878458   Err
        0.1D-04                 -
133     55:  3  0  3  2  1  1   S 5   3.7878757   -0.0300   3.7878458   Err
        0.1D-04                 -
134     56:  5  2  4  4  3  2   S 1   3.8083499   -0.1251   3.8082249   Err
        0.1D-04                 -
135     57:  5  3  3  5  2  3   S 1   3.9729123    0.1505   3.9730628   Err
        0.1D-04                 -
136     58:  5  3  3  5  2  3   S 2   3.9729122   -0.1344   3.9727778   Err
        0.1D-04                 -
137     59:  5  3  3  5  2  3   S 3   3.9729354    0.1273   3.9730628   Err
        0.1D-04                 -
138     60:  5  3  3  5  2  3   S 4   3.9729330   -0.1875   3.9727455   Err
        0.1D-04                 -
139     61:  5  3  3  5  2  3   S 5   3.9729377   -0.1021   3.9728356   Err
        0.1D-04                 -
140     62:  2  1  2  1  0  1   S 1   3.9943125    0.0301   3.9943426   Err
        0.1D-04                 -
141     63:  2  1  2  1  0  1   S 2   3.9943101   -0.0108   3.9942993   Err
        0.1D-04                 -
142     64:  2  1  2  1  0  1   S 3   3.9943075    0.0351   3.9943426   Err
        0.1D-04                 -
143     65:  2  1  2  1  0  1   S 4   3.9943049   -0.0055   3.9942993   Err
        0.1D-04                 -
144     66:  2  1  2  1  0  1   S 5   3.9943054   -0.0061   3.9942993   Err
        0.1D-04                 -
145     67:  4  3  2  4  2  2   S 1   4.2108317    0.1357   4.2109674   Err
        0.1D-04                 -
146     68:  4  3  2  4  2  2   S 2   4.2108108   -0.6833   4.2101275   Err
        0.1D-04                 -
147     69:  4  3  2  4  2  2   S 3   4.2108502    0.1173   4.2109674   Err
        0.1D-04                 -
148     70:  4  3  2  4  2  2   S 4   4.2108223   -0.7773   4.2100449   Err
        0.1D-04                 -
149     71:  4  3  2  4  2  2   S 5   4.2108363   -0.6252   4.2102111   Err
```

```
      0.1D-04                -
150   72:  3  0  3  2  1  2   S 1   4.2628510   -0.0293   4.2628217   Err
      0.1D-04                -
151   73:  3  3  1  3  2  1   S 1   4.3483114    0.1117   4.3484232   Err
      0.1D-04                -
152   74:  3  3  1  3  2  1   S 2   4.3481614   -2.2183   4.3459431   Err
      0.1D-04                -
153   75:  3  3  1  3  2  1   S 3   4.3483243    0.1369   4.3484612   Err
      0.1D-04                -
154   76:  3  3  1  3  2  1   S 4   4.3481361   -2.3690   4.3457671   Err
      0.1D-04                -
155   77:  3  3  1  3  2  1   S 5   4.3482149   -2.1042   4.3461107   Err
      0.1D-04                -
156   78:  3  3  0  3  2  1   S 2   4.3502282    2.2991   4.3525273   Err
      0.1D-04                -
157   79:  3  3  0  3  2  1   S 4   4.3502846    2.3919   4.3526765   Err
      0.1D-04                -
158   80:  3  3  0  3  2  1   S 5   4.3502079    2.1647   4.3523726   Err
      0.1D-04                -
159   81:  5  1  5  4  2  3   S 1   4.4005297   -0.0555   4.4004742   Err
      0.1D-04                -
160   82:  3  3  0  3  2  2   S 1   4.4529764    0.1333   4.4531097   Err
      0.1D-04                -
161   83:  3  3  0  3  2  2   S 2   4.4531256    2.3852   4.4555108   Err
      0.1D-04                -
162   84:  3  3  0  3  2  2   S 3   4.4529932    0.1165   4.4531097   Err
      0.1D-04                -
163   85:  3  3  0  3  2  2   S 4   4.4531805    2.4944   4.4556749   Err
      0.1D-04                -
164   86:  3  3  0  3  2  2   S 5   4.4531020    2.2428   4.4553448   Err
      0.1D-04                -
165   87:  2  1  1  1  0  1   S 1   4.4692678    0.0121   4.4692799   Err
      0.1D-04                -
166   88:  4  3  2  4  2  3   S 1   4.5049617    0.1136   4.5050753   Err
      0.1D-04                -
167   89:  4  3  2  4  2  3   S 2   4.5049368   -0.6546   4.5042822   Err
      0.1D-04                -
168   90:  4  3  2  4  2  3   S 3   4.5049735    0.1018   4.5050753   Err
      0.1D-04                -
169   91:  4  3  2  4  2  3   S 4   4.5049425   -0.7358   4.5042067   Err
      0.1D-04                -
170   92:  4  3  2  4  2  3   S 5   4.5049549   -0.5943   4.5043606   Err
      0.1D-04                -
171   93:  4  3  1  4  2  3   S 1   4.5172143    0.1136   4.5173279   Err
      0.1D-04                -
172   94:  4  3  1  4  2  3   S 2   4.5172353    0.8360   4.5180713   Err
      0.1D-04                -
173   95:  4  3  1  4  2  3   S 3   4.5172266    0.1013   4.5173279   Err
      0.1D-04                -
174   96:  4  3  1  4  2  3   S 4   4.5172544    0.8930   4.5181475   Err
      0.1D-04                -
175   97:  4  3  1  4  2  3   S 5   4.5172409    0.7461   4.5179869   Err
      0.1D-04                -
176   98:  5  2  3  5  1  5   S 1   4.5472346    0.0144   4.5472490   Err
      0.1D-04                -
177   99:  4  1  3  3  2  1   S 1   4.6514815   -0.0709   4.6514106   Err
      0.1D-04                -
178  100:  4  1  3  3  2  1   S 2   4.6514681   -0.1096   4.6513585   Err
     0.1D-04                 -
179  101:  4  1  3  3  2  1   S 3   4.6514454   -0.0348   4.6514106   Err
     0.1D-04                 -
180  102:  4  1  3  3  2  1   S 4   4.6514316   -0.0731   4.6513585   Err
     0.1D-04                 -
181  103:  4  1  3  3  2  1   S 5   4.6514324   -0.0739   4.6513585   Err
     0.1D-04                 -
182  104:  5  3  2  5  2  4   S 1   4.6553839    0.1027   4.6554866   Err
     0.1D-04                 -
183  105:  5  3  2  5  2  4   S 2   4.6553849    0.2806   4.6556654   Err
     0.1D-04                 -
184  106:  5  3  2  5  2  4   S 3   4.6553920    0.0946   4.6554866   Err
     0.1D-04                 -
185  107:  5  3  2  5  2  4   S 4   4.6553952    0.3062   4.6557014   Err
     0.1D-04                 -
```

```
186    108:  5  3  2  5  2  4   S 5   4.6553908    0.2257   4.6556164   Err
       0.1D-04                 -
187    109:  3  1  3  2  1  2   S 1   4.7661710    0.0146   4.7661856   Err
       0.1D-04                 -
188    110:  3  1  3  2  1  2   S 2   4.7661670   -0.0158   4.7661512   Err
       0.1D-04                 -
189    111:  3  1  3  2  1  2   S 3   4.7661580    0.0276   4.7661856   Err
       0.1D-04                 -
190    112:  3  1  3  2  1  2   S 4   4.7661534   -0.0022   4.7661512   Err
       0.1D-04                 -
191    113:  3  1  3  2  1  2   S 5   4.7661547   -0.0035   4.7661512   Err
       0.1D-04                 -
192    114:  6  3  3  6  2  5   S 1   4.9129131    0.0836   4.9129967   Err
       0.1D-04                 -
193    115:  3  0  3  2  0  2   S 1   4.9341891    0.0035   4.9341926   Err
       0.1D-04                 -
194    116:  3  0  3  2  0  2   S 2   4.9341840   -0.0273   4.9341567   Err
       0.1D-04                 -
195    117:  3  0  3  2  0  2   S 3   4.9341729    0.0197   4.9341926   Err
       0.1D-04                 -
196    118:  3  0  3  2  0  2   S 4   4.9341682   -0.0115   4.9341567   Err
       0.1D-04                 -
197    119:  3  0  3  2  0  2   S 5   4.9341673   -0.0106   4.9341567   Err
       0.1D-04                 -
198    120:  4  0  4  3  1  2   S 1   5.0452253    0.0112   5.0452365   Err
       0.1D-04                 -
199    121:  3  1  2  2  1  1   S 1   5.2393549   -0.0130   5.2393418   Err
       0.1D-04                 -
200    122:  6  2  5  5  3  3   S 1   5.3310658   -0.1440   5.3309218   Err
       0.1D-04                 -
201    123:  7  3  4  7  2  6   S 1   5.3444444    0.0445   5.3444889   Err
       0.1D-04                 -
202    124:  3  1  3  2  0  2   S 1   5.4375092    0.0433   5.4375524   Err
       0.1D-04                 -
203    125:  3  1  3  2  0  2   S 2   5.4375068    0.0048   5.4375116   Err
       0.1D-04                 -
204    126:  3  1  3  2  0  2   S 3   5.4375026    0.0498   5.4375524   Err
       0.1D-04                 -
205    127:  3  1  3  2  0  2   S 4   5.4374993    0.0123   5.4375116   Err
       0.1D-04                 -
206    128:  3  1  3  2  0  2   S 5   5.4375012    0.0104   5.4375116   Err
       0.1D-04                 -
207    129:  6  1  6  5  2  4   S 1   5.5181444   -0.0459   5.5180985   Err
       0.1D-04                 -
208    130:  8  4  4  7  5  2   S 1   5.6604214   -0.2592   5.6601621   Err
       0.1D-04                 -
209    131:  7  4  4  7  3  4   S 1   5.8572405    0.2079   5.8574485   Err
       0.1D-04                 -
210    132:  7  4  4  7  3  4   S 2   5.8572203   -0.7404   5.8564799   Err
       0.1D-04                 -
211    133:  7  4  4  7  3  4   S 3   5.8572700    0.1785   5.8574485   Err
       0.1D-04                 -
212    134:  2  2  1  1  1  0   S 1   5.9281606    0.0695   5.9282300   Err
       0.1D-04                 -
213    135:  2  2  0  1  1  0   S 1   5.9492115    0.0712   5.9492827   Err
       0.1D-04                 -
214    136:  2  2  0  1  1  0   S 3   5.9492090    0.0737   5.9492827   Err
       0.1D-04                 -
215    137:  2  2  0  1  1  0   S 4   5.9492118    0.2672   5.9494790   Err
       0.1D-04                 -
216    138:  2  2  0  1  1  0   S 5   5.9492088    0.2028   5.9494115   Err
       0.1D-04                 -
217    139:  4  0  4  3  1  3   S 1   5.9933644   -0.0250   5.9933394   Err
       0.1D-04                 -
218    140:  6  4  3  6  3  3   S 1   6.0303223    0.1854   6.0305077   Err
       0.1D-04                 -
219    141:  2  2  1  1  1  1   S 1   6.0864793    0.0665   6.0865458   Err
       0.1D-04                 -
220    142:  2  2  1  1  1  1   S 3   6.0864755    0.0704   6.0865458   Err
       0.1D-04                 -
221    143:  2  2  1  1  1  1   S 4   6.0864625   -0.1976   6.0862650   Err
       0.1D-04                 -
222    144:  2  2  1  1  1  1   S 5   6.0864656   -0.1329   6.0863326   Err
```

```
      0.1D-04                -
223   145:  2  2  0  1  1  1   S 1   6.1075303    0.0588   6.1075891   Err
      0.1D-04                -
224   146:  5  4  2  5  3  2   S 1   6.1240683    0.1625   6.1242308   Err
      0.1D-04                -
225   147:  5  4  2  5  3  3   S 3   6.1720986    0.1136   6.1722122   Err
      0.1D-04                -
226   148:  4  4  0  4  3  1   S 2   6.1695910    2.8930   6.1724841   Err
      0.1D-04                -
227   149:  4  4  0  4  3  1   S 4   6.1697048    2.9301   6.1726350   Err
      0.1D-04                -
228   150:  4  4  0  4  3  1   S 5   6.1695180    2.8002   6.1723183   Err
      0.1D-04                -
229   151:  5  4  1  5  3  3   S 1   6.1731868    0.1858   6.1733726   Err
      0.1D-04                -
230   152:  5  4  1  5  3  3   S 2   6.1735413    3.6667   6.1772079   Err
      0.1D-04                -
231   153:  5  4  1  5  3  3   S 3   6.1732162    0.1564   6.1733726   Err
      0.1D-04                -
232   154:  5  4  1  5  3  3   S 4   6.1736444    3.8219   6.1774663   Err
      0.1D-04                -
233   155:  5  4  1  5  3  3   S 5   6.1734832    3.4887   6.1769719   Err
      0.1D-04                -
234   156:  6  4  2  6  3  4   S 1   6.1746139    0.1718   6.1747857   Err
      0.1D-04                -
235   157:  6  4  2  6  3  4   S 2   6.1747055    2.3768   6.1770824   Err
      0.1D-04                -
236   158:  6  4  2  6  3  4   S 3   6.1746354    0.1503   6.1747857   Err
      0.1D-04                -
237   159:  6  4  2  6  3  4   S 4   6.1747526    2.5206   6.1772732   Err
      0.1D-04                -
238   160:  6  4  2  6  3  4   S 5   6.1747011    2.1968   6.1768979   Err
      0.1D-04                -
239   161:  4  4  1  4  3  2   S 2   6.1804570   -2.6540   6.1778030   Err
      0.1D-04                -
240   162:  4  4  1  4  3  2   S 4   6.1803843   -2.7312   6.1776531   Err
      0.1D-04                -
241   163:  4  4  1  4  3  2   S 5   6.1805712   -2.6248   6.1779464   Err
      0.1D-04                -
242   164:  4  4  0  4  3  2   S 1   6.1812126    0.1775   6.1813901   Err
      0.1D-04                -
243   165:  4  4  0  4  3  2   S 3   6.1812895    0.2688   6.1815584   Err
      0.1D-04                -
244   166:  7  4  3  7  3  5   S 1   6.2035499    0.1595   6.2037094   Err
      0.1D-04                -
245   167:  7  4  3  7  3  5   S 2   6.2035735    0.9730   6.2045465   Err
      0.1D-04                -
246   168:  7  4  3  7  3  5   S 3   6.2035681    0.1413   6.2037094   Err
      0.1D-04                -
247   169:  8  4  4  8  3  6   S 1   6.2888312    0.1437   6.2889749   Err
      0.1D-04                -
248   170:  3  1  2  2  0  2   S 1   6.3856482    0.0064   6.3856546   Err
      0.1D-04                -
249   171:  5  1  4  4  2  2   S 1   6.4331179   -0.0808   6.4330371   Err
      0.1D-04                -
250   172:  9  4  5  9  3  7   S 1   6.4715444    0.1389   6.4716833   Err
      0.1D-04                -
251   173: 10  4  7 10  3  8   S 1   6.4940089    0.1267   6.4941356   Err
      0.1D-04                -
252   174:  4  0  4  3  0  3   S 1   6.4966844   -0.0051   6.4966793   Err
      0.1D-04                -
253   175:  4  2  3  3  2  2   S 1   6.6715830    0.0030   6.6715860   Err
      0.1D-04                -
254   176:  4  1  4  3  0  3   S 1   6.8374091    0.0394   6.8374485   Err
      0.1D-04                -
255   177:  4  2  2  3  2  1   S 1   6.8628162   -0.0260   6.8627903   Err
      0.1D-04                -
256   178:  3  2  2  2  1  1   S 1   7.4418460    0.0774   7.4419234   Err
      0.1D-04                -
257   179:  3  2  2  2  1  1   S 2   7.4418397   -0.0119   7.4418278   Err
      0.1D-04                -
258   180:  3  2  2  2  1  1   S 3   7.4418399    0.0834   7.4419234   Err
      0.1D-04                -
```

```
259    181:  3  2  2  2  1  1   S 4   7.4418329   -0.0051   7.4418278   Err
       0.1D-04                -
260    182:  3  2  2  2  1  1   S 5   7.4418344   -0.0066   7.4418278   Err
       0.1D-04                -
261    183:  3  2  1  2  1  1   S 1   7.5447427    0.0656   7.5448083   Err
       0.1D-04                -
262    184:  5  0  5  4  1  4   S 1   7.6780172   -0.0157   7.6780016   Err
       0.1D-04                -
263    185:  5  0  5  4  1  4   S 2   7.6780073   -0.1078   7.6778995   Err
       0.1D-04                -
264    186:  5  0  5  4  1  4   S 3   7.6779731    0.0284   7.6780016   Err
       0.1D-04                -
265    187:  5  0  5  4  1  4   S 4   7.6779721   -0.0726   7.6778995   Err
       0.1D-04                -
266    188:  5  0  5  4  1  4   S 5   7.6779543   -0.0548   7.6778995   Err
       0.1D-04                -
267    189:  5  1  5  4  1  4   S 1   7.8886539   -0.0036   7.8886502   Err
       0.1D-04                -
268    190:  3  2  2  2  1  2   S 1   7.9168012    0.0643   7.9168655   Err
       0.1D-04                -
269    191:  3  2  2  2  1  2   S 2   7.9167909    0.0029   7.9167938   Err
       0.1D-04                -
270    192:  3  2  2  2  1  2   S 3   7.9167899    0.0757   7.9168655   Err
       0.1D-04                -
271    193:  3  2  2  2  1  2   S 4   7.9167796    0.0142   7.9167938   Err
       0.1D-04                -
272    194:  3  2  2  2  1  2   S 5   7.9167795    0.0143   7.9167938   Err
       0.1D-04                -
273    195:  4  1  3  3  0  3   S 1   8.4083284   -0.0070   8.4083214   Err
       0.1D-04                -
274    196:  4  2  2  3  1  2   S 1   9.1682040    0.0444   9.1682484   Err
       0.1D-04                -
275    197:  3  3  0  2  2  0   S 1   9.4479533    0.1300   9.4480833   Err
       0.1D-04                -
276    198:  3  3  0  2  2  0   S 2   9.4480892    2.1340   9.4502232   Err
       0.1D-04                -
277    199:  3  3  0  2  2  0   S 3   9.4479536    0.1297   9.4480833   Err
       0.1D-04                -
278    200:  3  3  0  2  2  0   S 4   9.4481255    2.2453   9.4503708   Err
       0.1D-04                -
279    201:  3  3  0  2  2  0   S 5   9.4480512    2.0224   9.4500736   Err
       0.1D-04                -
280    202:  3  3  1  2  2  1   S 1   9.4672360    0.1153   9.4673513   Err
       0.1D-04                -
281    203:  3  3  1  2  2  1   S 2   9.4670850   -1.9892   9.4650958   Err
       0.1D-04                -
282    204:  3  3  1  2  2  1   S 3   9.4672297    0.1215   9.4673513   Err
       0.1D-04                -
283    205:  3  3  1  2  2  1   S 4   9.4670428   -2.0950   9.4649478   Err
       0.1D-04                -
284    206:  3  3  1  2  2  1   S 5   9.4671171   -1.8873   9.4652299   Err
       0.1D-04                -
285    207:  5  2  4  4  1  4   S 1  11.8014357    0.0514  11.8014871   Err
       0.1D-04                -
286    Maximum (obs-calc)/err in line 154   0.0038219
287
288    indep.par: 10 stepw:1.0000 lambda:0.400D-05 cond.no:0.185D+03
289
290    ------------------------------------------------------
291    Iteration :  1
292    Sigma:0.876050D+00  Sigma/OldSigma:********  conv:-1
293    V1n_2  (1)    1723.439620  ************ 922.475% Max. Change
294
295    indep.par: 10 stepw:1.0000 lambda:0.400D-04 cond.no:0.184D+03
296
297    ------------------------------------------------------
298    Iteration :  2
299    Sigma:0.875689D+00  Sigma/OldSigma:********  conv:-2
300    V1n_2  (1)    1723.967147  ************ 922.162% Max. Change
301
302    indep.par: 10 stepw:1.0000 lambda:0.400D-03 cond.no:0.180D+03
303
304    ------------------------------------------------------
```

```
 Iteration :  3
 Switching to better derivatives (takes more time)
 Sigma:0.872082D+00  Sigma/OldSigma:********  conv:-3
 V1n_2  (1)    1729.250881  ************ 919.039% Max. Change

 indep.par: 10 stepw:1.0000 lambda:0.400D-02 cond.no:0.150D+03

 ------------------------------------------------------
 Iteration :  4
 Sigma:0.836286D+00  Sigma/OldSigma:********  conv:-4
 V1n_2  (1)    1782.733747  ************ 888.468% Max. Change

 indep.par: 10 stepw:1.0000 lambda:0.400D-01 cond.no:0.556D+02

 ------------------------------------------------------
 Iteration :  5
 Sigma:0.544543D+00  Sigma/OldSigma:********  conv:-5
 V1n_2  (1)    2322.531669  ************ 658.730% Max. Change

 indep.par: 10 stepw:1.0000 lambda:0.400D+00 cond.no:0.846D+01

 ------------------------------------------------------
 Iteration :  6
 Sigma:0.327561D-01  Sigma/OldSigma:********  conv:-6
 V1n_2  (1)    6264.749690  ************ 181.284% Max. Change

 indep.par: 10 stepw:1.0000 lambda:0.400D+01 cond.no:0.177D+01

 ------------------------------------------------------
 Iteration :  7
 Sigma:0.546575D-03  Sigma/OldSigma:0.563151  conv: 1

                Parameters     Change
 BJ              0.836003995 {-0.000000166}
 BK              0.887775563 { 0.000007395}
 B-              0.079157923 { 0.000000264}
 DJ              0.056500E-6 { 0.004140E-6}
 DJK             0.156281E-6 {-0.136077E-6}
 DK             -0.600744E-6 {-0.485672E-6}
 dj              0.020535E-6 {-0.001388E-6}
 dk             -0.837203E-6 {-0.255846E-6}
\F12            -0.353876210 {     derived}
\F             160.498761012 {     derived}  161.296325755 {     derived}
 V1n            14081.024224 {-1007.818546}   14407.296803 {-3214.447127}
\rho             0.005326176 {     derived}    0.006458864 {     derived}
\beta            1.613791315 {     derived}    0.913400410 {     derived}
\gamma           2.512532901 {     derived}    0.426130937 {     derived}
 DJK    (1)    0.156281E-6  -0.136077E-6  87.072% Max. Change

 indep.par: 10 stepw:1.0000 lambda:0.160D+01 cond.no:0.293D+01

 ------------------------------------------------------
 Iteration :  8
 Sigma:0.294863D-03  Sigma/OldSigma:0.539474  conv: 1

                Parameters     Change
 BJ              0.836003567 {-0.000000428}
 BK              0.887778621 { 0.000003058}
 B-              0.079158174 { 0.000000251}
 DJ              0.054132E-6 {-0.002368E-6}
 DJK             0.142187E-6 {-0.014094E-6}
 DK             -0.843203E-6 {-0.242459E-6}
 dj              0.018335E-6 {-0.002200E-6}
 dk             -0.805604E-6 { 0.031599E-6}
\F12            -0.353876323 {     derived}
\F             160.498760614 {     derived}  161.296325858 {     derived}
 V1n            13763.588965 { -317.435259}   13569.821891 { -837.474911}
\rho             0.005326174 {     derived}    0.006458866 {     derived}
\beta            1.613791399 {     derived}    0.913399520 {     derived}
\gamma           2.512533236 {     derived}    0.426130672 {     derived}
 DK     (1)   -0.843203E-6  -0.242459E-6  28.755% Max. Change

 indep.par: 10 stepw:1.0000 lambda:0.640D+00 cond.no:0.575D+01
```

```
 ------------------------------------------------------
 Iteration :  9
 Sigma:0.108047D-03  Sigma/OldSigma:0.366431  conv: 1

               Parameters     Change
 BJ             0.836002977 {-0.000000590}
 BK             0.887778230 {-0.000000391}
 B-             0.079157940 {-0.000000234}
 DJ             0.059028E-6 { 0.004897E-6}
 DJK            0.222549E-6 { 0.080361E-6}
 DK            -0.830408E-6 { 0.012795E-6}
 dj             0.017482E-6 {-0.000853E-6}
 dk            -0.567216E-6 { 0.238388E-6}
\F12           -0.353875909 {     derived}
\F            160.498759985 {     derived}  161.296325095 {     derived}
 Vln           13660.063203 { -103.525762}   13080.053221 { -489.768670}
\rho            0.005326170 {     derived}    0.006458861 {     derived}
\beta           1.613791407 {     derived}    0.913399396 {     derived}
\gamma          2.512533031 {     derived}    0.426130834 {     derived}
 dk     (1)   -0.567216E-6   0.238388E-6  42.028% Max. Change

 indep.par: 10 stepw:1.0000 lambda:0.256D+00 cond.no:0.124D+02

 ------------------------------------------------------
 Iteration : 10
 Sigma:0.510944D-04  Sigma/OldSigma:0.472890  conv: 1

               Parameters     Change
 BJ             0.836003003 { 0.000000026}
 BK             0.887778424 { 0.000000194}
 B-             0.079157094 {-0.000000846}
 DJ             0.079852E-6 { 0.020824E-6}
 DJK            0.297170E-6 { 0.074621E-6}
 DK            -0.693509E-6 { 0.136899E-6}
 dj             0.016920E-6 {-0.000562E-6}
 dk            -0.464978E-6 { 0.102237E-6}
\F12           -0.353875141 {     derived}
\F            160.498759900 {     derived}  161.296324746 {     derived}
 Vln           13695.955209 {   35.892006}   12909.994350 { -170.058871}
\rho            0.005326169 {     derived}    0.006458860 {     derived}
\beta           1.613791421 {     derived}    0.913399070 {     derived}
\gamma          2.512532057 {     derived}    0.426131605 {     derived}
 DJ     (1)    0.079852E-6   0.020824E-6  26.078% Max. Change

 indep.par: 10 stepw:1.0000 lambda:0.102D+00 cond.no:0.272D+02

 ------------------------------------------------------
 Iteration : 11
 Sigma:0.465065D-04  Sigma/OldSigma:0.910207  conv: 1

               Parameters     Change
 BJ             0.836003551 { 0.000000548}
 BK             0.887780666 { 0.000002242}
 B-             0.079156043 {-0.000001051}
 DJ             0.100384E-6 { 0.020531E-6}
 DJK            0.347351E-6 { 0.050181E-6}
 DK            -0.544041E-6 { 0.149468E-6}
 dj             0.015377E-6 {-0.001542E-6}
 dk            -0.568914E-6 {-0.103935E-6}
\F12           -0.353874375 {     derived}
\F            160.498760316 {     derived}  161.296325109 {     derived}
 Vln           13713.535625 {   17.580416}   12875.518082 {  -34.476268}
\rho            0.005326171 {     derived}    0.006458863 {     derived}
\beta           1.613791474 {     derived}    0.913398241 {     derived}
\gamma          2.512530790 {     derived}    0.426132607 {     derived}
 DK     (1)   -0.544041E-6   0.149468E-6  27.474% Max. Change

 indep.par: 10 stepw:1.0000 lambda:0.410D-01 cond.no:0.550D+02

 ------------------------------------------------------
 Iteration : 12
 Sigma:0.458416D-04  Sigma/OldSigma:0.985703  conv: 1
```

```
                Parameters     Change
 BJ              0.836003851 { 0.000000300}
 BK              0.887783124 { 0.000002458}
 B-              0.079155217 {-0.000000825}
 DJ              0.108144E-6 { 0.007760E-6}
 DJK             0.400095E-6 { 0.052744E-6}
 DK             -0.441474E-6 { 0.102567E-6}
 dj              0.015741E-6 { 0.000364E-6}
 dk             -0.731825E-6 {-0.162911E-6}
\F12            -0.353873736 {     derived}
\F             160.498760512 {     derived}  161.296325357 {     derived}
 V1n           13708.478559 {   -5.057065}   12871.021102 {   -4.496980}
\rho            0.005326171 {     derived}    0.006458866 {     derived}
\beta           1.613791537 {     derived}    0.913397360 {     derived}
\gamma          2.512529809 {     derived}    0.426133383 {     derived}
 DK     (1)   -0.441474E-6   0.102567E-6  23.233% Max. Change

 indep.par: 10 stepw:1.0000 lambda:0.164D-01 cond.no:0.951D+02

 ------------------------------------------------------
 Iteration : 13
 Sigma:0.457043D-04  Sigma/OldSigma:0.997006  conv: 1

                Parameters     Change
 BJ              0.836003872 { 0.000000021}
 BK              0.887784395 { 0.000001271}
 B-              0.079154837 {-0.000000380}
 DJ              0.105849E-6 {-0.002295E-6}
 DJK             0.452428E-6 { 0.052333E-6}
 DK             -0.420793E-6 { 0.020681E-6}
 dj              0.019007E-6 { 0.003265E-6}
 dk             -0.875443E-6 {-0.143618E-6}
\F12            -0.353873404 {     derived}
\F             160.498760483 {     derived}  161.296325372 {     derived}
 V1n           13704.269432 {   -4.209128}   12870.528161 {   -0.492941}
\rho            0.005326171 {     derived}    0.006458867 {     derived}
\beta           1.613791572 {     derived}    0.913396884 {     derived}
\gamma          2.512529370 {     derived}    0.426133730 {     derived}
 dj     (1)    0.019007E-6   0.003265E-6  17.179% Max. Change

 indep.par: 10 stepw:1.0000 lambda:0.655D-02 cond.no:0.135D+03

 ------------------------------------------------------
 Iteration : 14
 Sigma:0.456814D-04  Sigma/OldSigma:0.999499  conv: 1

                Parameters     Change
 BJ              0.836003844 {-0.000000028}
 BK              0.887784774 { 0.000000379}
 B-              0.079154728 {-0.000000109}
 DJ              0.102307E-6 {-0.003542E-6}
 DJK             0.486017E-6 { 0.033589E-6}
 DK             -0.434266E-6 {-0.013473E-6}
 dj              0.022058E-6 { 0.003052E-6}
 dk             -0.961650E-6 {-0.086207E-6}
\F12            -0.353873297 {     derived}
\F             160.498760441 {     derived}  161.296325343 {     derived}
 V1n           13702.462111 {   -1.807321}   12870.597605 {    0.069443}
\rho            0.005326171 {     derived}    0.006458867 {     derived}
\beta           1.613791584 {     derived}    0.913396734 {     derived}
\gamma          2.512529248 {     derived}    0.426133827 {     derived}
 dj     (1)    0.022058E-6   0.003052E-6  13.835% Max. Change

 indep.par: 10 stepw:1.0000 lambda:0.262D-02 cond.no:0.162D+03

 ------------------------------------------------------
 Iteration : 15
 Sigma:0.456797D-04  Sigma/OldSigma:0.999961  conv: 1

                Parameters     Change
 BJ              0.836003834 {-0.000000011}
 BK              0.887784851 { 0.000000078}
```

```
 B-            0.079154706 {-0.000000022}
 DJ            0.100985E-6 {-0.001322E-6}
 DJK           0.497055E-6 { 0.011038E-6}
 DK           -0.441471E-6 {-0.007205E-6}
 dj            0.023160E-6 { 0.001102E-6}
 dk           -0.989446E-6 {-0.027796E-6}
\F12          -0.353873274 {     derived}
\F           160.498760427 {     derived}  161.296325332 {     derived}
 V1n          13701.959627 {   -0.502484}   12870.651110 {    0.053506}
\rho           0.005326170 {     derived}    0.006458867 {     derived}
\beta          1.613791586 {     derived}    0.913396702 {     derived}
\gamma         2.512529224 {     derived}    0.426133846 {     derived}
 dj     (1)    0.023160E-6   0.001102E-6   4.756% Max. Change

 indep.par: 10 stepw:1.0000 lambda:0.105D-02 cond.no:0.176D+03

 ------------------------------------------------------
 Iteration : 16
 Sigma:0.456796D-04  Sigma/OldSigma:0.999999  conv: 1

                Parameters     Change
 BJ            0.836003832 {-0.000000002}
 BK            0.887784861 { 0.000000010}
 B-            0.079154703 {-0.000000003}
 DJ            0.100787E-6 {-0.000198E-6}
 DJK           0.498666E-6 { 0.001612E-6}
 DK           -0.442623E-6 {-0.001152E-6}
 dj            0.023324E-6 { 0.000164E-6}
 dk           -0.993488E-6 {-0.004041E-6}
\F12          -0.353873271 {     derived}
\F           160.498760425 {     derived}  161.296325331 {     derived}
 V1n          13701.901880 {   -0.057747}   12870.660008 {    0.008898}
\rho           0.005326170 {     derived}    0.006458867 {     derived}
\beta          1.613791586 {     derived}    0.913396698 {     derived}
\gamma         2.512529221 {     derived}    0.426133849 {     derived}
 dj     (1)    0.023324E-6   0.000164E-6   0.705% Max. Change

 indep.par: 10 stepw:1.0000 lambda:0.419D-03 cond.no:0.182D+03

 ------------------------------------------------------
 Iteration : 17
 Sigma:0.456796D-04  Sigma/OldSigma:1.000000  conv:-1
 dj     (1)    0.023334E-6   0.000010E-6   0.043% Max. Change

 indep.par: 10 stepw:1.0000 lambda:0.419D-02 cond.no:0.150D+03

 ------------------------------------------------------
 Iteration : 18
 Sigma:0.456796D-04  Sigma/OldSigma:1.000000  conv:-2
 dj     (1)    0.023333E-6   0.000008E-6   0.035% Max. Change

 indep.par: 10 stepw:1.0000 lambda:0.419D-01 cond.no:0.541D+02

 ------------------------------------------------------
 Iteration : 19
 Sigma:0.456796D-04  Sigma/OldSigma:1.000000  conv: 1

                Parameters     Change
 BJ            0.836003832 {-0.000000000}
 BK            0.887784861 { 0.000000000}
 B-            0.079154703 {-0.000000000}
 DJ            0.100783E-6 {-0.000004E-6}
 DJK           0.498695E-6 { 0.000029E-6}
 DK           -0.442644E-6 {-0.000021E-6}
 dj            0.023327E-6 { 0.000003E-6}
 dk           -0.993560E-6 {-0.000072E-6}
\F12          -0.353873271 {     derived}
\F           160.498760425 {     derived}  161.296325331 {     derived}
 V1n          13701.902084 {    0.000203}   12870.660190 {    0.000181}
\rho           0.005326170 {     derived}    0.006458867 {     derived}
\beta          1.613791586 {     derived}    0.913396698 {     derived}
\gamma         2.512529221 {     derived}    0.426133849 {     derived}
 dj     (1)    0.023327E-6   0.000003E-6   0.013% Max. Change
```

```
 indep.par: 10 stepw:1.0000 lambda:0.168D-01 cond.no:0.940D+02

 ------------------------------------------------------
 Iteration : 20
 Sigma:0.456796D-04  Sigma/OldSigma:1.000000  conv: 1

                Parameters     Change
 BJ              0.836003832 {-0.000000000}
 BK              0.887784861 { 0.000000000}
 B-              0.079154703 {-0.000000000}
 DJ              0.100779E-6 {-0.000004E-6}
 DJK             0.498731E-6 { 0.000036E-6}
 DK             -0.442670E-6 {-0.000026E-6}
 dj              0.023331E-6 { 0.000004E-6}
 dk             -0.993650E-6 {-0.000090E-6}
\F12            -0.353873271 {     derived}
\F             160.498760425 {     derived}  161.296325331 {     derived}
 V1n            13701.902726 {    0.000642}   12870.660395 {    0.000206}
\rho             0.005326170 {     derived}    0.006458867 {     derived}
\beta            1.613791586 {     derived}    0.913396698 {     derived}
\gamma           2.512529220 {     derived}    0.426133849 {     derived}
 dj     (1)     0.023331E-6   0.000004E-6   0.016% Max. Change

 indep.par: 10 stepw:1.0000 lambda:0.671D-02 cond.no:0.134D+03

 ------------------------------------------------------
 Iteration : 21
 Sigma:0.456796D-04  Sigma/OldSigma:1.000000  conv: 1

                Parameters     Change
 BJ              0.836003832 {-0.000000000}
 BK              0.887784862 { 0.000000000}
 B-              0.079154703 {-0.000000000}
 DJ              0.100775E-6 {-0.000003E-6}
 DJK             0.498757E-6 { 0.000026E-6}
 DK             -0.442689E-6 {-0.000019E-6}
 dj              0.023334E-6 { 0.000003E-6}
 dk             -0.993714E-6 {-0.000064E-6}
\F12            -0.353873271 {     derived}
\F             160.498760425 {     derived}  161.296325331 {     derived}
 V1n            13701.903325 {    0.000599}   12870.660536 {    0.000141}
\rho             0.005326170 {     derived}    0.006458867 {     derived}
\beta            1.613791586 {     derived}    0.913396698 {     derived}
\gamma           2.512529220 {     derived}    0.426133849 {     derived}
 dj     (1)     0.023334E-6   0.000003E-6   0.011% Max. Change

 indep.par: 10 stepw:1.0000 lambda:0.268D-02 cond.no:0.161D+03

 ------------------------------------------------------
 Iteration : 22
 Sigma:0.456796D-04  Sigma/OldSigma:1.000000  conv:-1
 dj     (1)     0.023334E-6   0.000001E-6   0.004% Max. Change

 indep.par: 10 stepw:1.0000 lambda:0.268D-01 cond.no:0.725D+02

 ------------------------------------------------------
 Iteration : 23
 Sigma:0.456796D-04  Sigma/OldSigma:1.000000  conv:-2
 dj     (1)     0.023334E-6   0.000000E-6   0.002% Max. Change

 indep.par: 10 stepw:1.0000 lambda:0.268D+00 cond.no:0.119D+02

 ------------------------------------------------------
 Iteration : 24
 Sigma:0.456796D-04  Sigma/OldSigma:1.000000  conv:-3
 dj     (1)     0.023334E-6   0.000000E-6   0.000% Max. Change

 indep.par: 10 stepw:1.0000 lambda:0.268D+01 cond.no:0.216D+01

 ------------------------------------------------------
 Iteration : 25
 Sigma:0.456796D-04  Sigma/OldSigma:1.000000  conv:-4
```

```
 dj     (1)    0.023334E-6   0.000000E-6   0.000% Max. Change

 indep.par: 10 stepw:1.0000 lambda:0.268D+02 cond.no:0.112D+01

 ------------------------------------------------------
 Iteration : 26
 Sigma:0.456796D-04  Sigma/OldSigma:1.000000  conv:-5
 dj     (1)    0.023334E-6   0.000000E-6   0.000% Max. Change

 indep.par: 10 stepw:1.0000 lambda:0.268D+03 cond.no:0.101D+01

 ------------------------------------------------------
 Iteration : 27
 Sigma:0.456796D-04  Sigma/OldSigma:1.000000  conv:-6
 dj     (1)    0.023334E-6   0.000000E-6   0.000% Max. Change

 indep.par: 10 stepw:1.0000 lambda:0.268D+04 cond.no:0.100D+01

 ------------------------------------------------------
 Iteration : 28
 Sigma:0.456796D-04  Sigma/OldSigma:1.000000  conv: 1

                Parameters     Change
 BJ             0.836003832 {-0.000000000}
 BK             0.887784862 { 0.000000000}
 B-             0.079154703 {-0.000000000}
 DJ             0.100775E-6 {-0.000000E-6}
 DJK            0.498757E-6 { 0.000000E-6}
 DK            -0.442689E-6 {-0.000000E-6}
 dj             0.023334E-6 { 0.000000E-6}
 dk            -0.993714E-6 {-0.000000E-6}
\F12           -0.353873271 {     derived}
\F            160.498760425 {     derived}  161.296325331 {     derived}
 V1n           13701.903326 {    0.000001}   12870.660536 {   -0.000000}
\rho            0.005326170 {     derived}    0.006458867 {     derived}
\beta           1.613791586 {     derived}    0.913396698 {     derived}
\gamma          2.512529220 {     derived}    0.426133849 {     derived}
 dj     (1)    0.023334E-6   0.000000E-6   0.000% Max. Change

 indep.par: 10 stepw:1.0000 lambda:0.268D+04 cond.no:0.187D+03
############################################################
  End at Cycle  28

       J K- K+  J K- K+    Sym   calc/GHz  diff/MHz     obs/GHz
   1:  9  3  7  9  2  7   S 1   2.1926429    0.1955   2.1928384   Err
   0.1D-04             -
   2:  9 K   3  9 K   0   S 2   2.1927714   -0.0696   2.1927017   Err
   0.1D-04             -
   3:  9  3  7  9  2  7   S 3   2.1927229    0.1155   2.1928384   Err
   0.1D-04             -
   4:  9 K   3  9 K   0   S 4   2.1928459   -0.1441   2.1927017   Err
   0.1D-04             -
   5:  9 K   3  9 K   0   S 5   2.1928570   -0.1552   2.1927017   Err
   0.1D-04             -
   6:  3  2  2  3  1  2   S 1   2.2025348    0.0385   2.2025733   Err
   0.1D-04             -
   7:  3 K   2  3 K  -1   S 2   2.2025101   -0.0149   2.2024952   Err
   0.1D-04             -
   8:  3  2  2  3  1  2   S 3   2.2025572    0.0161   2.2025733   Err
   0.1D-04             -
   9:  3 K   2  3 K  -1   S 4   2.2025250   -0.0298   2.2024952   Err
   0.1D-04             -
  10:  3 K   2  3 K  -1   S 5   2.2025399   -0.0447   2.2024952   Err
   0.1D-04             -
  11:  4  2  2  4  1  3   S 1   2.2113778    0.0126   2.2113904   Err
   0.1D-04             -
  12:  5  2  3  5  1  4   S 1   2.2205590    0.0040   2.2205630   Err
   0.1D-04             -
  13:  3  2  1  3  1  2   S 1   2.3054332    0.0318   2.3054650   Err
   0.1D-04             -
  14:  2  0  2  1  1  0   S 1   2.3560487   -0.0352   2.3560135   Err
   0.1D-04             -
  15:  6  2  4  6  1  5   S 1   2.3764729   -0.0208   2.3764520   Err
```

```
      0.1D-04             -
729   16:  2  2  1  2  1  1   S 1   2.4258659    0.0223   2.4258882   Err
      0.1D-04             -
730   17:  5  1  4  5  0  5   S 1   2.5373712   -0.0457   2.5373256   Err
      0.1D-04             -
731   18:  1  1  0  0  0  0   S 1   2.6389640   -0.0097   2.6389542   Err
      0.1D-04             -
732   19:  2  2  1  2  1  2   S 1   2.9008243    0.0122   2.9008365   Err
      0.1D-04             -
733   20:  2  2  0  2  1  2   S 1   2.9218756    0.0138   2.9218894   Err
      0.1D-04             -
734   21:  2 K  -2  2 K   1   S 2   2.9220684    0.0254   2.9220938   Err
      0.1D-04             -
735   22:  2  2  0  2  1  2   S 3   2.9218840    0.0054   2.9218894   Err
      0.1D-04             -
736   23:  2 K  -2  2 K   1   S 4   2.9220983    0.0271   2.9221255   Err
      0.1D-04             -
737   24:  2 K  -2  2 K   1   S 5   2.9220553   -0.0189   2.9220364   Err
      0.1D-04             -
738   25:  3  2  2  3  1  3   S 1   3.1506798    0.0162   3.1506960   Err
      0.1D-04             -
739   26:  3 K   2  3 K   1   S 2   3.1506065    0.0223   3.1506288   Err
      0.1D-04             -
740   27:  3  2  2  3  1  3   S 3   3.1506810    0.0150   3.1506960   Err
      0.1D-04             -
741   28:  3 K   2  3 K   1   S 4   3.1506152    0.0136   3.1506288   Err
      0.1D-04             -
742   29:  3 K   2  3 K   1   S 5   3.1506002    0.0286   3.1506288   Err
      0.1D-04             -
743   30:  4  1  4  3  2  2   S 1   3.1834265    0.0012   3.1834276   Err
      0.1D-04             -
744   31:  2  1  2  1  1  1   S 1   3.1857205   -0.0153   3.1857052   Err
      0.1D-04             -
745   32:  7  3  5  7  2  5   S 1   3.2042197    0.1311   3.2043507   Err
      0.1D-04             -
746   33:  7 K   3  7 K  -2   S 2   3.2042730   -0.0478   3.2042252   Err
      0.1D-04             -
747   34:  7  3  5  7  2  5   S 3   3.2042788    0.0719   3.2043507   Err
      0.1D-04             -
748   35:  7 K   3  7 K  -2   S 4   3.2043222   -0.0970   3.2042252   Err
      0.1D-04             -
749   36:  7 K   3  7 K  -2   S 5   3.2043420   -0.1168   3.2042252   Err
      0.1D-04             -
750   37:  3  2  1  3  1  3   S 1   3.2535782   -0.0078   3.2535704   Err
      0.1D-04             -
751   38:  3 K  -2  3 K   1   S 2   3.2535798    0.0331   3.2536129   Err
      0.1D-04             -
752   39:  3  2  1  3  1  3   S 3   3.2535756   -0.0052   3.2535704   Err
      0.1D-04             -
753   40:  3 K  -2  3 K   1   S 4   3.2535936    0.0193   3.2536129   Err
      0.1D-04             -
754   41:  3 K  -2  3 K   1   S 5   3.2535607    0.0522   3.2536129   Err
      0.1D-04             -
755   42:  2  0  2  1  0  1   S 1   3.3229905   -0.0238   3.3229667   Err
      0.1D-04             -
756   43:  4  2  3  4  1  4   S 1   3.4881706   -0.0129   3.4881577   Err
      0.1D-04             -
757   44:  2  1  1  1  1  0   S 1   3.5023591   -0.0382   3.5023209   Err
      0.1D-04             -
758   45:  6  3  4  6  2  4   S 1   3.6322952    0.1054   3.6324006   Err
      0.1D-04             -
759   46:  6 K   3  6 K  -2   S 2   3.6322790   -0.0304   3.6322486   Err
      0.1D-04             -
760   47:  6  3  4  6  2  4   S 3   3.6323435    0.0571   3.6324006   Err
      0.1D-04             -
761   48:  6 K   3  6 K  -2   S 4   3.6323131   -0.0645   3.6322486   Err
      0.1D-04             -
762   49:  6 K   3  6 K  -2   S 5   3.6323415   -0.0929   3.6322486   Err
      0.1D-04             -
763   50:  4  2  2  4  1  4   S 1   3.7823059   -0.0142   3.7822917   Err
      0.1D-04             -
764   51:  3  0  3  2  1  1   S 1   3.7878991   -0.0039   3.7878952   Err
      0.1D-04             -
```

```
765     52:  3 K   0  2 K  -1   S 2   3.7878705   -0.0248   3.7878458   Err
        0.1D-04              -
766     53:  3  0  3  2  1  1   S 3   3.7878662    0.0290   3.7878952   Err
        0.1D-04              -
767     54:  3 K   0  2 K  -1   S 4   3.7878365    0.0093   3.7878458   Err
        0.1D-04              -
768     55:  3 K   0  2 K  -1   S 5   3.7878388    0.0070   3.7878458   Err
        0.1D-04              -
769     56:  5  2  4  4  3  2   S 1   3.8082663   -0.0414   3.8082249   Err
        0.1D-04              -
770     57:  5  3  3  5  2  3   S 1   3.9729852    0.0775   3.9730628   Err
        0.1D-04              -
771     58:  5 K   3  5 K  -2   S 2   3.9728079   -0.0302   3.9727778   Err
        0.1D-04              -
772     59:  5  3  3  5  2  3   S 3   3.9730240    0.0388   3.9730628   Err
        0.1D-04              -
773     60:  5 K   3  5 K  -2   S 4   3.9728230   -0.0775   3.9727455   Err
        0.1D-04              -
774     61:  5 K   3  5 K  -2   S 5   3.9728704   -0.0349   3.9728356   Err
        0.1D-04              -
775     62:  2  1  2  1  0  1   S 1   3.9943426    0.0000   3.9943426   Err
        0.1D-04              -
776     63:  2 K   1  1 K   0   S 2   3.9943291   -0.0298   3.9942993   Err
        0.1D-04              -
777     64:  2  1  2  1  0  1   S 3   3.9943350    0.0076   3.9943426   Err
        0.1D-04              -
778     65:  2 K   1  1 K   0   S 4   3.9943189   -0.0196   3.9942993   Err
        0.1D-04              -
779     66:  2 K   1  1 K   0   S 5   3.9943242   -0.0249   3.9942993   Err
        0.1D-04              -
780     67:  4  3  2  4  2  2   S 1   4.2109142    0.0533   4.2109674   Err
        0.1D-04              -
781     68:  4 K   3  4 K  -2   S 2   4.2101500   -0.0225   4.2101275   Err
        0.1D-04              -
782     69:  4  3  2  4  2  2   S 3   4.2109446    0.0228   4.2109674   Err
        0.1D-04              -
783     70:  4 K   3  4 K  -2   S 4   4.2101173   -0.0723   4.2100449   Err
        0.1D-04              -
784     71:  4 K   3  4 K  -2   S 5   4.2102444   -0.0334   4.2102111   Err
        0.1D-04              -
785     72:  3  0  3  2  1  2   S 1   4.2628575   -0.0358   4.2628217   Err
        0.1D-04              -
786     73:  3  3  1  3  2  1   S 1   4.3483999    0.0233   4.3484232   Err
        0.1D-04              -
787     74:  3 K   3  3 K  -2   S 2   4.3459570   -0.0138   4.3459431   Err
        0.1D-04              -
788     75:  3  3  1  3  2  1   S 3   4.3484177    0.0435   4.3484612   Err
        0.1D-04              -
789     76:  3 K   3  3 K  -2   S 4   4.3458554   -0.0883   4.3457671   Err
        0.1D-04              -
790     77:  3 K   3  3 K  -2   S 5   4.3461123   -0.0016   4.3461107   Err
        0.1D-04              -
791     78:  3 K  -3  3 K  -2   S 2   4.3525303   -0.0030   4.3525273   Err
        0.1D-04              -
792     79:  3 K  -3  3 K  -2   S 4   4.3526757    0.0008   4.3526765   Err
        0.1D-04              -
793     80:  3 K  -3  3 K  -2   S 5   4.3524393   -0.0668   4.3523726   Err
        0.1D-04              -
794     81:  5  1  5  4  2  3   S 1   4.4004939   -0.0196   4.4004742   Err
        0.1D-04              -
795     82:  3  3  0  3  2  2   S 1   4.4530667    0.0430   4.4531097   Err
        0.1D-04              -
796     83:  3 K  -3  3 K   2   S 2   4.4555036    0.0072   4.4555108   Err
        0.1D-04              -
797     84:  3  3  0  3  2  2   S 3   4.4530990    0.0107   4.4531097   Err
        0.1D-04              -
798     85:  3 K  -3  3 K   2   S 4   4.4556541    0.0208   4.4556749   Err
        0.1D-04              -
799     86:  3 K  -3  3 K   2   S 5   4.4553998   -0.0551   4.4553448   Err
        0.1D-04              -
800     87:  2  1  1  1  0  1   S 1   4.4693009   -0.0211   4.4692799   Err
        0.1D-04              -
801     88:  4  3  2  4  2  3   S 1   4.5050495    0.0258   4.5050753   Err
```

```
      0.1D-04                -
802    89:  4 K   3  4 K   2   S 2   4.5042874   -0.0052   4.5042822   Err
      0.1D-04                -
803    90:  4  3  2  4  2  3   S 3   4.5050689    0.0064   4.5050753   Err
      0.1D-04                -
804    91:  4 K   3  4 K   2   S 4   4.5042517   -0.0450   4.5042067   Err
      0.1D-04                -
805    92:  4 K   3  4 K   2   S 5   4.5043628   -0.0022   4.5043606   Err
      0.1D-04                -
806    93:  4  3  1  4  2  3   S 1   4.5173029    0.0251   4.5173279   Err
      0.1D-04                -
807    94:  4 K  -3  4 K   2   S 2   4.5180656    0.0057   4.5180713   Err
      0.1D-04                -
808    95:  4  3  1  4  2  3   S 3   4.5173242    0.0037   4.5173279   Err
      0.1D-04                -
809    96:  4 K  -3  4 K   2   S 4   4.5181480   -0.0005   4.5181475   Err
      0.1D-04                -
810    97:  4 K  -3  4 K   2   S 5   4.5180253   -0.0384   4.5179869   Err
      0.1D-04                -
811    98:  5  2  3  5  1  5   S 1   4.5472874   -0.0384   4.5472490   Err
      0.1D-04                -
812    99:  4  1  3  3  2  1   S 1   4.6514561   -0.0455   4.6514106   Err
      0.1D-04                -
813   100:  4 K  -1  3 K  -2   S 2   4.6513403    0.0182   4.6513585   Err
      0.1D-04                -
814   101:  4  1  3  3  2  1   S 3   4.6513940    0.0166   4.6514106   Err
      0.1D-04                -
815   102:  4 K  -1  3 K  -2   S 4   4.6512743    0.0842   4.6513585   Err
      0.1D-04                -
816   103:  4 K  -1  3 K  -2   S 5   4.6512819    0.0766   4.6513585   Err
      0.1D-04                -
817   104:  5  3  2  5  2  4   S 1   4.6554728    0.0137   4.6554866   Err
      0.1D-04                -
818   105:  5 K  -3  5 K   2   S 2   4.6556518    0.0136   4.6556654   Err
      0.1D-04                -
819   106:  5  3  2  5  2  4   S 3   4.6554864    0.0002   4.6554866   Err
      0.1D-04                -
820   107:  5 K  -3  5 K   2   S 4   4.6556873    0.0141   4.6557014   Err
      0.1D-04                -
821   108:  5 K  -3  5 K   2   S 5   4.6556434   -0.0270   4.6556164   Err
      0.1D-04                -
822   109:  3  1  3  2  1  2   S 1   4.7661892   -0.0036   4.7661856   Err
      0.1D-04                -
823   110:  3 K   1  2 K   1   S 2   4.7661684   -0.0172   4.7661512   Err
      0.1D-04                -
824   111:  3  1  3  2  1  2   S 3   4.7661683    0.0173   4.7661856   Err
      0.1D-04                -
825   112:  3 K   1  2 K   1   S 4   4.7661407    0.0105   4.7661512   Err
      0.1D-04                -
826   113:  3 K   1  2 K   1   S 5   4.7661543   -0.0031   4.7661512   Err
      0.1D-04                -
827   114:  6  3  3  6  2  5   S 1   4.9130067   -0.0099   4.9129967   Err
      0.1D-04                -
828   115:  3  0  3  2  0  2   S 1   4.9342095   -0.0169   4.9341926   Err
      0.1D-04                -
829   116:  3 K   0  2 K   0   S 2   4.9341754   -0.0187   4.9341567   Err
      0.1D-04                -
830   117:  3  0  3  2  0  2   S 3   4.9341804    0.0122   4.9341926   Err
      0.1D-04                -
831   118:  3 K   0  2 K   0   S 4   4.9341508    0.0059   4.9341567   Err
      0.1D-04                -
832   119:  3 K   0  2 K   0   S 5   4.9341416    0.0151   4.9341567   Err
      0.1D-04                -
833   120:  4  0  4  3  1  2   S 1   5.0452277    0.0088   5.0452365   Err
      0.1D-04                -
834   121:  3  1  2  2  1  1   S 1   5.2393758   -0.0340   5.2393418   Err
      0.1D-04                -
835   122:  6  2  5  5  3  3   S 1   5.3309654   -0.0437   5.3309218   Err
      0.1D-04                -
836   123:  7  3  4  7  2  6   S 1   5.3445501   -0.0611   5.3444889   Err
      0.1D-04                -
837   124:  3  1  3  2  0  2   S 1   5.4375412    0.0112   5.4375524   Err
      0.1D-04                -
```

```
838    125:  3 K   1  2 K   0   S 2   5.4375334   -0.0218   5.4375116   Err
       0.1D-04                -
839    126:  3  1  3  2  0  2   S 3   5.4375331    0.0193   5.4375524   Err
       0.1D-04                -
840    127:  3 K   1  2 K   0   S 4   5.4375161   -0.0045   5.4375116   Err
       0.1D-04                -
841    128:  3 K   1  2 K   0   S 5   5.4375345   -0.0229   5.4375116   Err
       0.1D-04                -
842    129:  6  1  6  5  2  4   S 1   5.5181014   -0.0029   5.5180985   Err
       0.1D-04                -
843    130:  8  4  4  7  5  2   S 1   5.6601582    0.0039   5.6601621   Err
       0.1D-04                -
844    131:  7  4  4  7  3  4   S 1   5.8573406    0.1079   5.8574485   Err
       0.1D-04                -
845    132:  7 K   4  7 K  -3   S 2   5.8564947   -0.0149   5.8564799   Err
       0.1D-04                -
846    133:  7  4  4  7  3  4   S 3   5.8573896    0.0589   5.8574485   Err
       0.1D-04                -
847    134:  2  2  1  1  1  0   S 1   5.9282250    0.0050   5.9282300   Err
       0.1D-04                -
848    135:  2  2  0  1  1  0   S 1   5.9492763    0.0064   5.9492827   Err
       0.1D-04                -
849    136:  2  2  0  1  1  0   S 3   5.9492721    0.0106   5.9492827   Err
       0.1D-04                -
850    137:  2 K  -2  1 K  -1   S 4   5.9494611    0.0179   5.9494790   Err
       0.1D-04                -
851    138:  2 K  -2  1 K  -1   S 5   5.9494312   -0.0197   5.9494115   Err
       0.1D-04                -
852    139:  4  0  4  3  1  3   S 1   5.9933727   -0.0333   5.9933394   Err
       0.1D-04                -
853    140:  6  4  3  6  3  3   S 1   6.0304409    0.0668   6.0305077   Err
       0.1D-04                -
854    141:  2  2  1  1  1  1   S 1   6.0865449    0.0010   6.0865458   Err
       0.1D-04                -
855    142:  2  2  1  1  1  1   S 3   6.0865381    0.0077   6.0865458   Err
       0.1D-04                -
856    143:  2 K   2  1 K   1   S 4   6.0862885   -0.0236   6.0862650   Err
       0.1D-04                -
857    144:  2 K   2  1 K   1   S 5   6.0863184    0.0143   6.0863326   Err
       0.1D-04                -
858    145:  2  2  0  1  1  1   S 1   6.1075961   -0.0070   6.1075891   Err
       0.1D-04                -
859    146:  5  4  2  5  3  2   S 1   6.1241991    0.0318   6.1242308   Err
       0.1D-04                -
860    147:  5  4  2  5  3  3   S 3   6.1722302   -0.0180   6.1722122   Err
       0.1D-04                -
861    148:  4 K  -4  4 K  -3   S 2   6.1724931   -0.0090   6.1724841   Err
       0.1D-04                -
862    149:  4 K  -4  4 K  -3   S 4   6.1726394   -0.0044   6.1726350   Err
       0.1D-04                -
863    150:  4 K  -4  4 K  -3   S 5   6.1724154   -0.0971   6.1723183   Err
       0.1D-04                -
864    151:  5  4  1  5  3  3   S 1   6.1733209    0.0516   6.1733726   Err
       0.1D-04                -
865    152:  5 K  -4  5 K   3   S 2   6.1772152   -0.0072   6.1772079   Err
       0.1D-04                -
866    153:  5  4  1  5  3  3   S 3   6.1733815   -0.0089   6.1733726   Err
       0.1D-04                -
867    154:  5 K  -4  5 K   3   S 4   6.1774352    0.0311   6.1774663   Err
       0.1D-04                -
868    155:  5 K  -4  5 K   3   S 5   6.1770649   -0.0930   6.1769719   Err
       0.1D-04                -
869    156:  6  4  2  6  3  4   S 1   6.1747432    0.0426   6.1747857   Err
       0.1D-04                -
870    157:  6 K  -4  6 K   3   S 2   6.1770831   -0.0007   6.1770824   Err
       0.1D-04                -
871    158:  6  4  2  6  3  4   S 3   6.1747819    0.0039   6.1747857   Err
       0.1D-04                -
872    159:  6 K  -4  6 K   3   S 4   6.1772658    0.0074   6.1772732   Err
       0.1D-04                -
873    160:  6 K  -4  6 K   3   S 5   6.1769692   -0.0713   6.1768979   Err
       0.1D-04                -
874    161:  4 K   4  4 K   3   S 2   6.1778258   -0.0228   6.1778030   Err
```

```
      0.1D-04                -
875   162:  4 K   4  4 K   3   S 4   6.1777487   -0.0956   6.1776531   Err
      0.1D-04                -
876   163:  4 K   4  4 K   3   S 5   6.1779740   -0.0277   6.1779464   Err
      0.1D-04                -
877   164:  4  4  0  4  3  2   S 1   6.1813524    0.0377   6.1813901   Err
      0.1D-04                -
878   165:  4  4  0  4  3  2   S 3   6.1815084    0.0500   6.1815584   Err
      0.1D-04                -
879   166:  7  4  3  7  3  5   S 1   6.2036772    0.0322   6.2037094   Err
      0.1D-04                -
880   167:  7 K  -4  7 K   3   S 2   6.2045399    0.0067   6.2045465   Err
      0.1D-04                -
881   168:  7  4  3  7  3  5   S 3   6.2037086    0.0008   6.2037094   Err
      0.1D-04                -
882   169:  8  4  4  8  3  6   S 1   6.2889633    0.0116   6.2889749   Err
      0.1D-04                -
883   170:  3  1  2  2  0  2   S 1   6.3856862   -0.0316   6.3856546   Err
      0.1D-04                -
884   171:  5  1  4  4  2  2   S 1   6.4330870   -0.0499   6.4330371   Err
      0.1D-04                -
885   172:  9  4  5  9  3  7   S 1   6.4716942   -0.0109   6.4716833   Err
      0.1D-04                -
886   173: 10  4  7 10  3  8   S 1   6.4941373   -0.0017   6.4941356   Err
      0.1D-04                -
887   174:  4  0  4  3  0  3   S 1   6.4967043   -0.0251   6.4966793   Err
      0.1D-04                -
888   175:  4  2  3  3  2  2   S 1   6.6715971   -0.0111   6.6715860   Err
      0.1D-04                -
889   176:  4  1  4  3  0  3   S 1   6.8374380    0.0106   6.8374485   Err
      0.1D-04                -
890   177:  4  2  2  3  2  1   S 1   6.8628339   -0.0437   6.8627903   Err
      0.1D-04                -
891   178:  3  2  2  2  1  1   S 1   7.4419106    0.0127   7.4419234   Err
      0.1D-04                -
892   179:  3 K   2  2 K  -1   S 2   7.4418351   -0.0072   7.4418278   Err
      0.1D-04                -
893   180:  3  2  2  2  1  1   S 3   7.4419000    0.0233   7.4419234   Err
      0.1D-04                -
894   181:  3 K   2  2 K  -1   S 4   7.4418170    0.0108   7.4418278   Err
      0.1D-04                -
895   182:  3 K   2  2 K  -1   S 5   7.4418319   -0.0041   7.4418278   Err
      0.1D-04                -
896   183:  3  2  1  2  1  1   S 1   7.5448090   -0.0007   7.5448083   Err
      0.1D-04                -
897   184:  5  0  5  4  1  4   S 1   7.6780216   -0.0201   7.6780016   Err
      0.1D-04                -
898   185:  5 K   0  4 K   1   S 2   7.6779234   -0.0238   7.6778995   Err
      0.1D-04                -
899   186:  5  0  5  4  1  4   S 3   7.6779261    0.0754   7.6780016   Err
      0.1D-04                -
900   187:  5 K   0  4 K   1   S 4   7.6779175   -0.0179   7.6778995   Err
      0.1D-04                -
901   188:  5 K   0  4 K   1   S 5   7.6777384    0.1612   7.6778995   Err
      0.1D-04                -
902   189:  5  1  5  4  1  4   S 1   7.8886645   -0.0142   7.8886502   Err
      0.1D-04                -
903   190:  3  2  2  2  1  2   S 1   7.9168690   -0.0035   7.9168655   Err
      0.1D-04                -
904   191:  3 K   2  2 K   1   S 2   7.9167749    0.0189   7.9167938   Err
      0.1D-04                -
905   192:  3  2  2  2  1  2   S 3   7.9168493    0.0163   7.9168655   Err
      0.1D-04                -
906   193:  3 K   2  2 K   1   S 4   7.9167558    0.0380   7.9167938   Err
      0.1D-04                -
907   194:  3 K   2  2 K   1   S 5   7.9167545    0.0393   7.9167938   Err
      0.1D-04                -
908   195:  4  1  3  3  0  3   S 1   8.4083661   -0.0446   8.4083214   Err
      0.1D-04                -
909   196:  4  2  2  3  1  2   S 1   9.1682672   -0.0188   9.1682484   Err
      0.1D-04                -
910   197:  3  3  0  2  2  0   S 1   9.4480601    0.0233   9.4480833   Err
      0.1D-04                -
```

```
 198:  3 K  -3  2 K  -2   S 2   9.4502101    0.0130   9.4502232   Err
0.1D-04             -
 199:  3  3  0  2  2  0   S 3   9.4480643    0.0191   9.4480833   Err
0.1D-04             -
 200:  3 K  -3  2 K  -2   S 4   9.4503116    0.0592   9.4503708   Err
0.1D-04             -
 201:  3 K  -3  2 K  -2   S 5   9.4500991   -0.0255   9.4500736   Err
0.1D-04             -
 202:  3  3  1  2  2  1   S 1   9.4673430    0.0083   9.4673513   Err
0.1D-04             -
 203:  3 K   3  2 K   2   S 2   9.4651038   -0.0080   9.4650958   Err
0.1D-04             -
 204:  3  3  1  2  2  1   S 3   9.4673287    0.0226   9.4673513   Err
0.1D-04             -
 205:  3 K   3  2 K   2   S 4   9.4649921   -0.0442   9.4649478   Err
0.1D-04             -
 206:  3 K   3  2 K   2   S 5   9.4652050    0.0249   9.4652299   Err
0.1D-04             -
 207:  5  2  4  4  1  4   S 1  11.8014864    0.0008  11.8014871   Err
0.1D-04             -
 Maximum (obs-calc)/err in line   1   0.0001955

 RMS deviations (MHz), B and V sorted
  B  V   n splittings MHz
  B  V   n abs. freq. MHz
  1  1 207          0.044449          0.046597

            Parameters  and  Errors
 BJ            0.836003832 { 0.000001210}
 BK            0.887784862 { 0.000001959}
 B-            0.079154703 { 0.000000853}
 DJ            0.100775E-6 { 0.028545E-6}
 DJK           0.498757E-6 { 0.103956E-6}
 DK           -0.442689E-6 { 0.123991E-6}
 dj            0.023334E-6 { 0.011752E-6}
 dk           -0.993714E-6 { 0.271231E-6}
\F12          -0.353873271 {     derived}
\F           160.498760425 {     derived}  161.296325331 {     derived}
 V1n          13701.903325 {  167.434315}   12870.660536 {    6.801193}
\rho           0.005326170 {     derived}    0.006458867 {     derived}
\beta          1.613791586 {     derived}    0.913396698 {     derived}
\gamma         2.512529220 {     derived}    0.426133849 {     derived}
 F0          159.647000000 {     fixed  }  160.297000000 {     fixed  }
 epsil         2.420000000 {     fixed  }    0.502000000 {     fixed  }
 delta         1.592000000 {     fixed  }    1.195000000 {     fixed  }

 Standard Deviation       0.045680 MHz

------------------------------------ B =  1
 Rotational Constants and Errors (in GHz)
      B_z       1.723788693       0.000002176
      B_x       0.915158535       0.000001525
      B_y       0.756849129       0.000001434
 Ray's kappa   -0.67256
 F0(calc)     159.647000000       0.000000000
 I_alpha        3.165603300       0.000000000
 <(i,x)  <(i,y)  <(i,z)      138.6412       48.6671       91.2149
d<(i,x) d<(i,y) d<(i,z)        0.0000        0.0000        0.0000

 F0(calc)     160.297000000       0.000000000
 I_alpha        3.152766864       0.000000000
 <(i,x)  <(i,y)  <(i,z)       35.3684       63.4101       68.4685
d<(i,x) d<(i,y) d<(i,z)        0.0000        0.0000        0.0000

 V1n_1      5.467489 kj +/- 0.066812 kj         1.305855 kcal +/- 0.015957 kcal
          457.046234 cm +/-   5.5850 cm    s=  37.942566
 V1n_2      5.135797 kj +/- 0.002714 kj         1.226634 kcal +/- 0.000648 kcal
          429.318963 cm +/-   0.2269 cm    s=  35.640734

F(calc)       160.498760425
F(calc)       161.296325331

 Errors of fitted linear combinations
```

```
      0.000001210    0.000001959    0.000000853  167.434314698    0.000000029
      0.000000104    0.000000124    0.000000012    0.000000271    6.801193179

 Correlation Matrix of fitted linear combinations
 BJ      1.000
 BK     -0.120  1.000
 B-      0.065 -0.221  1.000
 V1n_1  -0.007 -0.009  0.045  1.000
 DJ      0.755 -0.120  0.075 -0.051  1.000
 DJK    -0.082  0.375 -0.277 -0.032 -0.415  1.000
 DK      0.099  0.427 -0.024  0.024  0.383 -0.572  1.000
 dj      0.000  0.174  0.193 -0.017 -0.339  0.819 -0.531  1.000
 dk      0.014 -0.301  0.352  0.040  0.359 -0.910  0.445 -0.807  1.000
 V1n_2  -0.063 -0.029  0.050 -0.018 -0.111  0.072 -0.184  0.071 -0.050  1.000
 strongest correlation between  9 and  6 (-0.9103)

 Freedom Cofreedom Matrix of linear comb.
 BJ      0.573
 BK      0.996  0.377
 B-      0.996  0.976  0.421
 V1n_1   0.998  0.999  0.999  0.991
 DJ      0.629  0.992  0.997  0.996  0.515
 DJK     0.998  0.722  0.944  0.999  0.952  0.186
 DK      0.997  0.623  0.992  0.999  0.950  0.681  0.321
 dj      0.999  0.978  0.677  1.000  0.970  0.720  0.914  0.250
 dk      0.992  0.954  0.867  0.999  0.955  0.620  0.914  0.690  0.256
 V1n_2   0.999  0.992  0.999  1.000  0.997  0.993  0.979  0.999  0.997  0.962
 minimum cofreedom between  9 and  6 ( 0.6195)

 Eigenvalues and Eigenvector Matrix of SVD-FIT

0.168169D-01   0.019-0.102 0.061 0.004 0.111-0.686 0.244-0.463 0.478-0.011
0.800918D-01   0.012-0.637 0.350 0.004-0.049-0.138-0.613 0.219 0.158 0.039
0.128011D+00  -0.159-0.249-0.661-0.011-0.163 0.130-0.354-0.546-0.086 0.016
0.175622D+00   0.654-0.243-0.210-0.026 0.645-0.001 0.032-0.004-0.223-0.044
0.369786D+00  -0.187-0.405-0.171 0.009-0.236-0.430 0.377 0.257-0.564-0.075
0.932342D+00   0.018 0.082 0.014-0.399 0.016-0.124-0.036-0.014-0.137 0.893
0.981523D+00   0.158 0.025 0.013 0.900-0.106-0.032-0.002-0.044-0.063 0.383
0.168214D+01   0.637 0.138 0.201-0.160-0.611-0.094-0.094-0.241-0.201-0.140
0.249427D+01   0.282-0.095-0.505-0.052-0.299 0.029 0.152 0.488 0.541 0.094
0.313939D+01   0.027-0.513 0.259-0.048-0.113 0.532 0.513-0.274 0.123 0.132
```
